# Design and rationale of the Botswana Smoking Abstinence Reinforcement Trial: a protocol for a stepped-wedge cluster randomized trial

**DOI:** 10.1186/s43058-024-00588-7

**Published:** 2024-05-08

**Authors:** Florence Bada, Megan E. Mansfield, Lillian Okui, Milton Montebatsi, Carlo DiClemente, Roy Tapera, Kaizer Ikgopoleng, Selebaleng Mokonopi, Jessica F. Magidson, Eberechukwu Onukwugha, Ndwapi Ndwapi, Seth Himelhoch, Bontle Mbongwe, Man Charurat

**Affiliations:** 1 Division of Epidemiology and Prevention, Institute of Human Virology, University of Maryland School of Medicine, Baltimore, MD USA; 2 Botswana University of Maryland Medicine Health Initiative, Gaborone, Botswana; 3 Department of Psychology, University of Maryland Baltimore County, Baltimore, MD USA; 4 School of Public Health, University of Botswana, Gaborone, Botswana; 5 Anti-Tobacco Network, University of Botswana, Gaborone, Botswana; 6 Department of Psychology and the Center for Substance Use, Addiction & Health Research (CESAR), University of Maryland, College Park, Maryland, USA; 7 Department of Practice, Sciences, and Health Outcomes Research, University of Maryland School of Pharmacy, Baltimore, MD USA; 8 Department of Psychiatry, University of Kentucky School of Medicine, Lexington, KY USA; 9 Department of Epidemiology and Public Health, University of Maryland School of Medicine, Baltimore, MD USA

**Keywords:** Smoking cessation, Brief intervention, Type 2 hybrid effectiveness-implementation, Varenicline

## Abstract

**Background:**

With expanded and sustained availability of HIV treatment resulting in substantial improvements in life expectancy, the need to address modifiable risk factors associated with leading causes of death among people living with HIV/AIDS (PLWH), such as tobacco smoking, has increased. Tobacco use is highly prevalent among PLWH, especially in southern Africa, where HIV is heavily concentrated, and many people who smoke would like to quit but are unable to do so without assistance. SBIRT (Screening, Brief Intervention and Referral to Treatment) is a well-established evidence-based approach successful at supporting smoking cessation in a variety of settings. Varenicline is efficacious in supporting smoking cessation. We intend to assess the effectiveness of SBIRT and varenicline on smoking cessation among PLWH in Botswana and the effectiveness of our implementation.

**Methods:**

BSMART (Botswana Smoking Abstinence Reinforcement Trial) is a stepped-wedge, cluster randomized, hybrid Type 2 effectiveness-implementation study guided by the RE-AIM framework, to evaluate the effectiveness and implementation of an SBIRT intervention consisting of the 5As compared to an enhanced standard of care. SBIRT will be delivered by trained lay health workers (LHWs), followed by referral to treatment with varenicline prescribed and monitored by trained nurse prescribers in a network of outpatient HIV care facilities. Seven hundred and fifty people living with HIV who smoke daily and have been receiving HIV care and treatment at one of 15 health facilities will be recruited if they are up to 18 years of age and willing to provide informed consent to participate in the study.

**Discussion:**

BSMART tests a scalable approach to achieve and sustain smoking abstinence implemented in a sustainable way. Integrating an evidence-based approach such as SBIRT, into an HIV care system presents an important opportunity to establish and evaluate a modifiable cancer prevention strategy in a middle-income country (MIC) setting where both LHW and non-physician clinicians are widely used. The findings, including the preliminary cost-effectiveness, will provide evidence to guide the Botswanan government and similar countries as they strive to provide affordable smoking cessation support at scale.

**Clinical trial registration:**

NCT05694637 Registered on 7 December 2022 on clinicaltrials.gov, https://clinicaltrials.gov/search?locStr=Botswana&country=Botswana&cond=Smoking%20Cessation&intr=SBIRT

**Supplementary Information:**

The online version contains supplementary material available at 10.1186/s43058-024-00588-7.

Contributions to the literature

- SBIRT (Screening, Brief Intervention and Referral to Treatment) is a well-established evidence-based approach endorsed by the US preventive services taskforce successful at supporting smoking cessation in a variety of settings.
- Varenicline is highly effective in supporting smoking cessation among people living with HIV (PLWH).
- BSMART (Botswana Smoking Abstinence Reinforcement Trial) is a hybrid type 2 implementation-effectiveness study guided by the RE-AIM framework with a stepped-wedge design.
- BSMART will evaluate the effectiveness of SBIRT and varenicline delivered to PLWH by lay health workers and nurse prescribers as compared to an enhanced standard of care in a middle-income country setting. It will also evaluate the effectiveness of our implementation.

## Background

Expanded and sustained access to HIV treatment, resulting in substantial improvements in life-expectancy [1], has led to the need to address modifiable risk factors associated with leading causes of death among people living with HIV (PLWH), including cardiovascular disease and cancer [2–4]. PLWH smoke at higher rates than the general population [5–10], and among PLWH, tobacco use has been consistently shown to impact both HIV-related [11, 12] and non-HIV related co-morbidities [13–18]. Tobacco smoking has also been identified as the leading cause of premature mortality [19–21]. In addition, many PLWH who smoke would like to quit [7, 22, 23].

Although many interventions are effective in helping people who smoke to quit [24], research examining outcomes of smoking treatments among PLWH is concentrated in high-income countries. There is a critical need to assess the effectiveness of smoking cessation interventions among PLWH in lower- and middle-income countries and assess the delivery of these interventions within the clinical infrastructure available in these settings.

One evidence-based approach endorsed by the US Preventive services taskforce [24], and successful at supporting smoking cessation in a variety of settings, is the Screening, Brief Intervention, and Referral to Treatment (SBIRT) approach [25–29]. SBIRT not only increases the likelihood of making a tobacco quit attempt among patients who receive a brief intervention, but it is also strongly and consistently associated with increased satisfaction with care provided [27]. Even low-intensity SBIRT may prompt quit attempts, decrease cigarette use, and support quitting, if offered routinely [30]. In addition, the reach of SBIRT interventions can be increased by delivering SBIRT using cadres of staff other than medical providers [31].

The likelihood of a successful quit attempt is increased if counseling is provided along with pharmacologic therapy [32], especially for individuals who smoke and are willing to quit. Several pharmacotherapies are available to assist with smoking cessation [24]. However, while the use of nicotine replacement therapy (NRT) has mixed results [33, 34], varenicline, a high-affinity partial agonist for the nicotinic acetylcholine receptor subtype, has been shown to be more efficacious than NRT or bupropion in a large pharmacotherapy smoking cessation trial [35] and when used among PLWH [36].

Botswana, an upper middle-income country with a high prevalence of HIV of 20.8% among adults (15‐64 years), has achieved epidemic control of HIV with 98% of PLWH in Botswana on ARVs and 98% with viral load suppression [37]. In addition, Botswana has a high prevalence of cigarette smoking of 14.2% among persons aged 15 years and above [38]. Botswana is particularly vulnerable to the sequelae of tobacco smoking because it is in a region expected to face the largest growth in tobacco consumption in the world [39]. Though a high proportion of Batswana contemplated quitting in 2017, only 7% of persons who attempted to quit in 2016 were able to successfully quit [38].

**Fig. 1:**
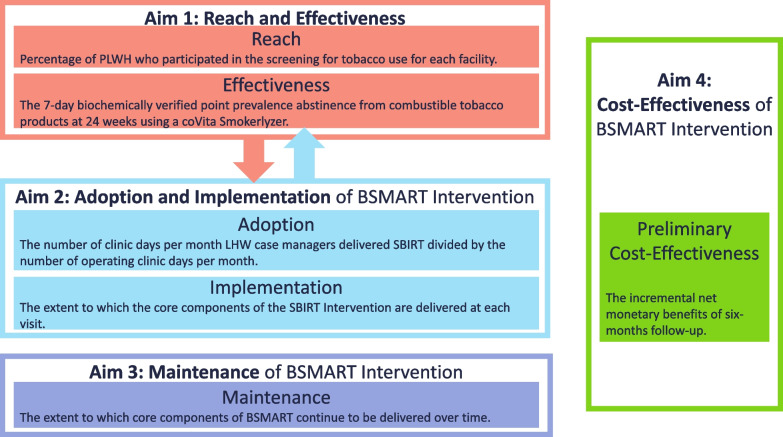
Integrated RE-AIM Framework with BSMART key outcomes

We intend to assess the effectiveness of the SBIRT and varenicline intervention on smoking cessation among PLWH in Botswana and the effectiveness of our implementation, guided by the implementation science RE-AIM framework [40] (Fig. 1). As we implement BSMART, the SBIRT intervention will continue to be adapted to fit the cultural and practical context of Botswana. We hypothesize that the BSMART Intervention (SBIRT and varenicline) will increase the proportion of smokers who quit, increase the number of quit attempts, and increase the length of abstinence in unsuccessful quit attempts as compared to enhanced standard of care. We also hypothesize that the BSMART Intervention will decrease the number of cigarettes smoked daily and decrease the number of days of smoking in a month as compared to enhanced standard of care. We believe we can build a collaborative network of key stakeholders to adapt the BSMART intervention to the local context and leverage existing infrastructure and staff to successfully implement it and integrate it into existing services.

## Methods

### Study design

**Fig. 2:**
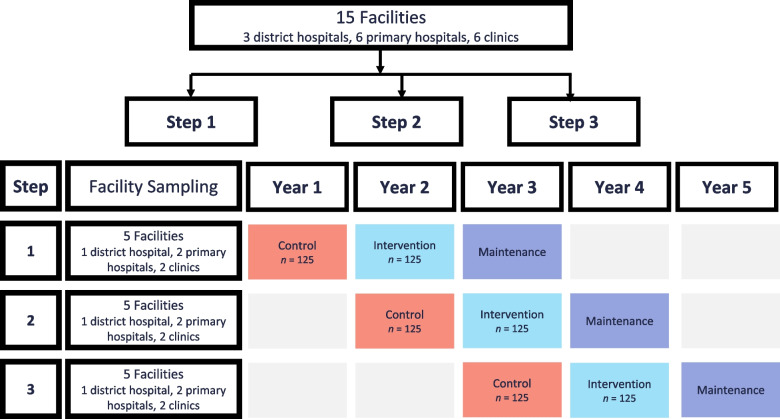
Time periods and sampling for stepped-wedge implementation

BSMART is a stepped-wedge cluster randomized trial. This design includes an initial period when no clusters are exposed to the intervention then at regular steps, five clusters are randomized to cross from the control to the intervention [41]. We will sequentially roll out the BSMART Intervention to 15 HIV care and treatment facilities. These facilities will be assigned in three steps, each providing data for a 12- month control/pre-intervention, a 12-month intervention, and a 12-month maintenance period (see Fig. 2). We will stratify Botswana’s HIV treatment and care facilities into three levels – district hospitals, primary hospitals, and primary clinics and randomly assign each of the three levels of facilities to one of three study steps. Each step will have five sites with representation from three levels of facilities: one district hospital, two primary hospitals and two primary clinics.

Our study employs a hybrid type 2 effectiveness-implementation design, which has equal attention to both effectiveness and implementation outcomes [42], to examine the effect of the BSMART Intervention (SBIRT and varenicline) on achieving abstinence from combustible tobacco products as compared to an enhanced standard of care among PLWH in Botswana who smoke and the effectiveness of our implementation.

### Conceptual framework

The BSMART study is guided by the RE-AIM framework which conceptualizes the effect of an intervention as the summation of five factors—the reach, effectiveness, adoption, implementation, and maintenance [40] (see Fig. 1). The use of this framework supports the cohesive evaluation of the adapted intervention and future sustainability.

### Study setting

We will sequentially roll out the BSMART Intervention to 15 HIV care and treatment facilities that are part of the ABLE (Accelerating Botswana through the Last Mile to Epidemic Control) project which is a five-year CDC-funded HIV care and treatment project. ABLE operates in 12 different PEPFAR health districts, 53 health facilities (13 hospitals and 40 clinics) and provides laboratory services that support all health districts. These 15 selected facilities are high volume facilities with over 1000 PLWH currently active and on antiretroviral therapy. LHWs and expert clients are in place in these facilities to ensure retention in treatment. Nurse prescribers are leveraged to manage the high volume of clients and to prescribe ARVs to stable clients on HIV treatment.

### Study participants

Participants will be drawn from the population of patients with HIV receiving care at 15 selected health facilities that are part of the ABLE project. Eligibility criteria are displayed in Table 1. Study participants will receive HIV care and treatment according to national standards.

**Table 1:** Eligibility criteria for the BSMART study

Inclusion Criteria	Exclusion Criteria
• Living with HIV • Engaged in HIV care as defined by being on ART for at least 6 months at one of 15 selected health facilities (or four reserve facilities), • Self-reported current daily smoker, • Aged 18 years or older, and • Willing/able to provide informed consent in English or Setswana	• Pregnant or • Nursing

### Study intervention

#### Enhanced standard of care

During the control phase, LHWs will provide an enhanced standard of care. This consists of providing participants with a brochure and a two- minute counseling session on the hazards of smoking and the benefits of quitting.

### BSMART intervention – SBIRT and varenicline

SBIRT is a comprehensive, integrated, public health approach to the delivery of early intervention and treatment services to persons with substance use disorders endorsed by the US Preventive Services Taskforce [24]. Trained LHWs will oversee the screening and brief intervention procedures using the 5As of SBIRT. The first “A” begins the intervention with LHWs “*Asking*” eligible clinic clients about smoking. Participants who report being daily smokers will be linked to a research assistant who will obtain informed consent and enroll the PLWH who smoke in the trial. The next 3 “A” s (*Advise, Assess, Assist*) constitute the brief intervention which will be delivered by LHWs using motivational enhancing conversations. Their efforts will focus the conversation on increasing insight and awareness regarding smoking, offering information and *Advice*, and *Assessing* motivation toward behavioral change. For participants who are motivated for treatment, a referral (*Assist*) will be made to a clinic nurse prescriber for evaluation for treatment with varenicline. For those not ready to make a quit attempt, the LHW will encourage consideration of quitting and *Arrange* for a follow-up conversation that will also encourage, advise, and assist in obtaining varenicline use.

Treatment with varenicline will be offered and provided to those motivated to quit. Smokers will initiate medication treatment with varenicline with a quit date scheduled for day 8 following the first study dose of the medication. They will meet with the nurse prescriber at baseline who will provide medical clearance and sign off on prescription orders. All medication will be provided to participants by the care team. Participants will receive a supply of medication for the first four weeks with subsequent weekly calls to ensure proper dosing and monitoring for adverse events. Participants will receive medication for the next eight weeks, at their Week four visit to the health facility. The dosage of varenicline will be in accordance with package labeling though dosage adjustments will be permitted to control adverse effects throughout the trial. This will allow us to balance internal validity with good clinical practice.

### Study procedures

All study procedures have been approved by the University of Maryland’s Institutional Review Board and the Botswana Health Research Development Committee.

### Participant recruitment (screening, informed consent, and enrollment)

Recruitment activities will begin at the HIV care services through the introduction of BSMART by LHWs. The LHWs will provide tobacco use screening within the context of HIV outpatient care. They will identify individuals who initially screen positive for tobacco use and link them with the research assistant stationed in the HIV clinic. Research assistants will obtain informed consent in the participant’s choice of English or Setswana language from eligible individuals. We plan to screen approximately 6,900 persons living with HIV (230 PLWH per facility per year) for tobacco smoking and subsequently identify 750 patients who are interested in and agree to participate in a smoking cessation trial; a total of 375 in the control period and 375 in the intervention period across three waves of implementation at five facilities each.

### Follow-up visits

Follow up visits will occur at Weeks 4,12, and 24 during both the control and intervention phases supplemented with phone calls at Weeks 1,2,3, and 8 for participants on varenicline during the intervention phase. The procedures completed for visits in the control phase are depicted in Table 2 while those completed in the intervention phase are depicted in Table 3.

**Table 2:** Control procedures

	**Baseline**	**Week 4**	**Week 12**	**Week 24**
Intake smoking questionnaire	X			
Receipt of SOC brochure	X			
Follow-up smoking questionnaire		X	X	X
Stages of change	X	X	X	X
Breath CO	X		X	X
Semi−structured interview^a^				^X^

^a^only for selected participants

**Table 3:** Intervention procedures

	**Baseline**	**Week 1**	**Week 2**	**Week 3**	**Week 4**	**Week 8**	**Week 12**	**Week 24**
Intake smoking questionnaire	X							
Follow-up smoking questionnaire					X		X	X
Stages of change	X				X		X	X
SBIRT intervention	X							
Breath CO	X						X	X
Adherence to varenicline and Adverse events monitoring		X	X	X	X	X	X	
Semi-structured interview^a^								X

^a^only for selected participants

### Planning for sustainability

#### Active stakeholder engagement

**Fig. 3:**
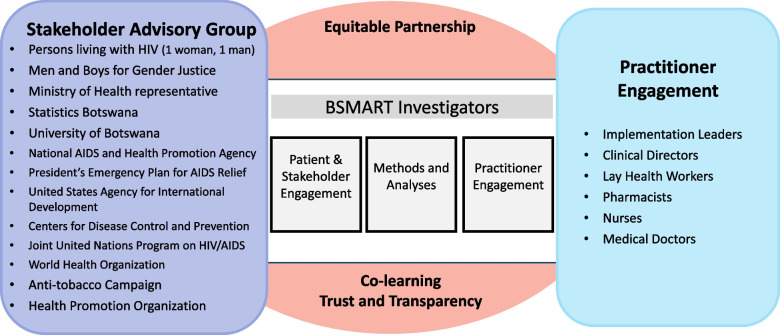
BSMART Implementation Governance Structure

We consider our stakeholders integral partners in all our efforts related to the development, implementation, and dissemination of our BSMART study. Guided by the Consortium for Cancer Implementation Science’s Tools for Stakeholder and Community Engagement, we will establish an implementation governance structure that integrates the voices of stakeholders, practitioners, and researchers (Fig. 3).

The membership of the Stakeholder Advisory Group reflects the diverse constituents who are invested in the successful implementation of the smoking cessation program within the context of HIV service delivery and frontrunners in health policy development for Botswana. The practitioner group consists of four implementation leaders who are health care professionals at country level, medical doctors, clinical directors, pharmacists, nurses, and LHWs. The stakeholders have worked together with the BSMART research team to select the intervention, develop the implementation design, and craft the dissemination plan. The Stakeholder Advisory Group and Practitioner Engagement Core will monitor target enrollment milestones, assist with developing lay descriptions of the study, and inform outreach efforts to encourage diverse patient participation. They will contribute to the data analysis process, specifically reviewing and interpreting results.

#### Adaptation of SBIRT

The BSMART Intervention will be adapted to suit the Botswana context through discussions with our stakeholders, and through a series of focus group discussions prior to and during the implementation of the BSMART intervention. Pre-implementation, the focus will be their initial thoughts on SBIRT and how SBIRT can be modified to make it more culturally appropriate and feasible. Post-introduction of the BSMART intervention, focus group discussions will elicit challenges the LHWs and nurse prescribers are experiencing with implementing the BSMART intervention and its integration with HIV care.

#### Capacity building

We will train four implementation leaders who are key health care professionals in the Botswana Ministry of Health to be trainers on SBIRT who can continue to train LHWs and nurse prescribers on the BSMART intervention if it is adopted for implementation at national level. Before each implementation period, the implementation leaders will facilitate a 3-day kick-off orientation for the BSMART study at a central location and train LHWs and nurse prescribers working in randomly selected sites. Nurse prescribers will attend a three-day didactic and hands-on centralized training on the SBIRT intervention and how to integrate it into their current work at sites. They will also receive in-service training at their facilities prior to the introduction of the BSMART intervention and as needed during the trial.

Research Assistants will undergo a two-week training before they commence work on the BSMART study. They will be trained in research ethics, good clinical practice, and the study protocol. They will also be trained in data collection using mobile REDCap and how to collect and manage qualitative data. They will attend the training sessions for LHWs and nurse prescribers to reinforce their understanding of the BSMART study.

#### Integration into existing systems/ structures

The sites selected for the BSMART study are part of the ABLE project which has been supported by some of the investigators for the last eight years to provide care and treatment to over 110,000 PLWH through 53 facilities in 12 districts. This ensures accessibility to the research team and willingness to collaborate. We will review how to integrate SBIRT into the care team and develop and pilot the new clinical workflow between LHWs and nurse prescribers.

### Study outcomes

 Reach—the percentage of PLWH who participated in the screening for tobacco use for each facility.

Effectiveness measures—The clinical effectiveness endpoint at 24 weeks is the 7-day point prevalence abstinence from combustible tobacco products validated primarily by breath CO < 6 ppm. The failure for this measure is any smoking (even a puff) during a 7-day window or a CO level ≥ 6 ppm.

Secondary Outcomes include quit attempts, length of quitting during each attempt, number of cigarettes smoked, and number of days of using combustible tobacco products for at least 24 h within the past month and the last 3 months.

Adoption—the number of clinic days per month LHWs deliver SBIRT divided by the number of operating clinic days per month will be analyzed monthly and for the 12 months of the maintenance phase.

Implementation effectiveness will be assessed by measuring the extent of integration of BSMART into the clinical setting within participating facilities. *Implementation with fidelity*, the extent to which the core components of the SBIRT Intervention are delivered at each visit will be measured using a Fidelity checklist applied to a randomly selected subset of recorded brief interventions by two implementation leaders.

Maintenance is the extent to which core components of BSMART continue to be delivered over time. The Preliminary cost effectiveness is the difference in cost between the control and intervention phases.

### Measuring sustainability

To identify constructs that are particularly important for sustainability, we will use the 40-item Program Sustainability Assessment Tool [43] to capture measures of coalition, funding stability, partnerships, organizational capacity, program evaluation, program adaptation, communications, and strategic planning in a variety of public health programs during the implementation and maintenance period. Total sustainability score and domain-specific scores will be determined and compared between periods.

### Data collection

Data will be collected on forms listed in Appendix A using mobile REDCap.

### Statistical analysis

We will compare *Reach*—the percentage of PLWH who were screened for tobacco use for each facility between the control and implementation group using chi-square test.

*Effectiveness* – We will compare the clinical effectiveness endpoint between the control group and implementation group at the individual level with a generalized linear mixed model with a binary distribution using the jack-knife method to estimate standard errors to account for grouping within clusters and by incorporating a log-link function to estimate the relative risk as a measure of effect [41, 44]. We will include random effects to account for the clustering within facilities and periods and fixed effects for types of facilities. The model with unique covariance structure that produces the lowest Bayesian Information Criterion (BIC) value will be selected as the best model. The covariance structures that will be considered in the model are the first order of autocorrelation covariance structure, unstructured covariance structure, and Toeplitz covariance structure. We will use the Satterthwaite method to adjust for denominator degree of freedom for the test for fixed effects. The random coefficients will be modeled using G-side random effects, and we will obtain the subject-specific estimates by defining the appropriate variance–covariance structures.

We will perform sex-stratified and age-stratified analyses and models as secondary analyses. Given the high quality of data and experience in conducting studies in Botswana, we do not expect to have missing observations in the variables required for the primary analysis.

As analysis of secondary outcomes at 24 weeks, we will compare 30-day point prevalence abstinence between the implementation and the control groups in a similar model to the one used in the analysis of the primary outcome. We will compare longitudinal differences in the Stages of Change Algorithm between the implementation and the control groups using random-mixed effects regression. We will use generalized estimating equation models for binary repeated measures to assess factors associated with completion of the varenicline medication [45].

Implementation – We will assess implementation monthly for the intervention period. We will use Pearson’s correlation to assess the strength of correlation between qualitatively derived construct ratings from qualitative interviews and implementation effectiveness across facilities.

*Maintenance* – We will reassess RE-AIM measures 12—24 months after BSMART implementation to provide a standardized evaluation approach to foster understanding of whether the impact and implementation delivery are maintained and to highlight where sustainability issues arise.

**Fig. 4:**
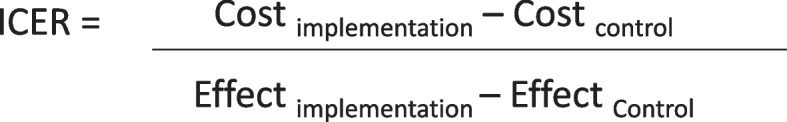
Incremental cost-effectiveness ratio

*Preliminary cost-effectiveness*—The incremental cost-effectiveness ratio (ICER) is calculated as the ratio of the difference in mean costs and the difference in mean 7-day point prevalence abstinence at 6 months. The ICER (Fig. 4) quantifies the additional cost associated with a unit change in the 7-day point prevalence abstinence at six months, comparing between the implementation and control groups. Net benefit (NB) regression [46, 47] provides an appropriate approach to quantify the ICER using the incremental net monetary benefit (INMB) and produces a confidence interval around the ICER.

**Fig. 5:**
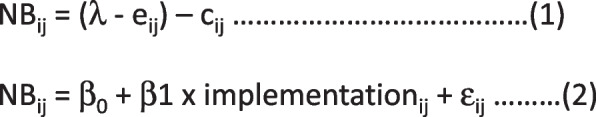
Net Benefit

In Eq. 1 of Fig. 5 we define NB as the difference between the monetized total effect measure (the product of lambda, the willingness to pay per unit of effect, and the total effect units available, e) and the associated costs. The NB regression model is specified in Eq. 2 of Fig. 5 as the difference between effects and costs, estimated via a parametric regression framework where errors are represented by the final term in Eq. 2, epsilon. We will identify the initial value of lambda following a targeted literature review and consultations with onsite study collaborators. In Eq. 2 of Fig. 5, the NMB, represented by the $\beta$﻿ coefficient on the indicator variable for receipt of intervention, will be estimated using the NB regression model.

### Sensitivity analysis

We will represent uncertainty in the estimated incremental cost-effectiveness ratios (ICERs) using the cost-effectiveness acceptability curve [46, 48]. In addition, we will represent parameter uncertainty in the choice of the economically preferred intervention (between control and intervention periods) by the conditional net benefit (cNB) curve. The cNB curve plots the cNB (i.e., the net benefit value given a particular value of the parameter of interest) across centiles of the distribution of the parameter of interest.

Sensitivity analyses will include probabilistic one-way sensitivity analyses (POSA) [49] of cost input parameters (such as drug costs). Uncertainty in cost inputs will be investigated via designed simulations that use alternative values of cost inputs as determined by draws from assigned distributions (for example gamma, and lognormal), with distributional parameters estimated from data collected from ongoing studies in Botswana. The POSA varies a specific input parameter value across its full distribution while accounting for concurrent variation in all other parameter values using Monte Carlo simulation. The cNB curve developed from the planned POSA will allow decision makers to identify the impact of the value taken by a specific cost parameter on the value of the BSMAART intervention.

### Power and sample size

**Fig. 6:**
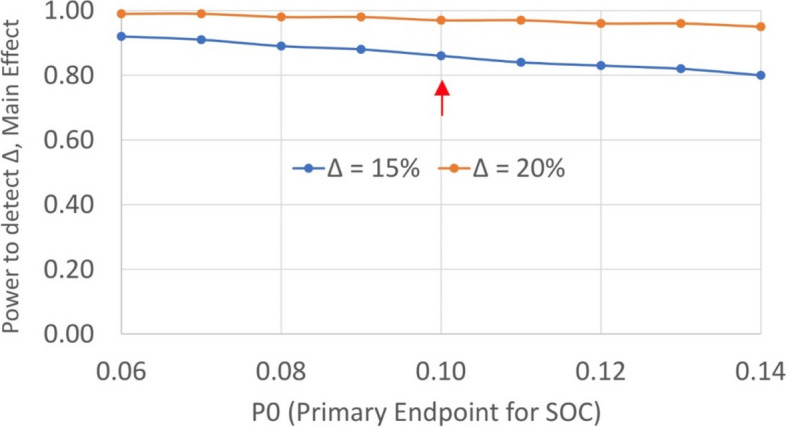
Power to Detect the Primary Endpoint, Main Effect versus Enhanced Standard of Care

Our sample size is based on the hypothesis that our intervention will have a substantial impact on primary endpoints compared to the control phase (Fig. 6).

### Main effect

Based on a meta-analysis of 24 randomized controlled trials in LMIC [50], smoking abstinence at 6 months follow-up (P_control_) for usual care averages 10% (range, 8% to 14%). Assuming withdrawal from trial follow-up or loss to follow-up of 5%, our sample of 25 PLWH per cluster per period has power of 87% to detect at least 10% increase (main effect, ∆ = (P_intervention_ -P_control_) in the primary endpoint at 24 weeks with the assumption that the event rate for those in the control phase is 10% and two-sided alpha at intra-cluster correlation of 0.02.

## Discussion

Botswana, with its HIV prevalence of 20.8%, yet with 98% on ARVs and 98% with undetectable viral loads [37], is a prime example of a country that may need to increase its focus on modifiable risk factors that are on the increase due to improvements in life expectancy. Botswana is particularly vulnerable because it is in a region expected to face the largest growth in tobacco consumption in the world [39]. The Botswana Government recognizing that smokers, especially those who are living with HIV, need additional assistance beyond standard smoking cessation services has passed a tobacco control law which aims to discourage smoking initiation, encourage quitting, and reduce tobacco use overall. This study is responsive to the Botswana Government’s multi-sectoral strategy for the prevention and control of non- communicable diseases (NCDs) which seeks to reduce the burden of NCDs and their modifiable risk factors including tobacco use through evidence-based cost-effective approaches. The study further aligns well with the recognition by the Botswanan government of the high smoking rates in the country and the need for an implementation science study that uses proven evidence-based interventions for smoking cessation that can be deployed at high volume in HIV care facilities at an acceptable cost.

We intend to deliver an evidence-based intervention, adapted to the Botswana context to assist PLWH who smoke to quit smoking and remain abstinent. SBIRT has been proven effective in a variety of settings [25–29] and this study will add evidence to the utility of SBIRT in a middle-income country in sub-Saharan Africa.

Using a stepped wedge design will minimize the practical, logistical, and financial constraints associated with large scale project implementation, control for the effect of time, and ensure that all the treatment facilities involved in the trial eventually offer the BSMART intervention [41, 44]. Delivering SBIRT using cadres of staff other than medical providers often results in increased reach of the SBIRT intervention [31]. Our delivery of SBIRT through LHWs and nurse prescribers considered critical in filling the human resource constraints in Botswana, is an appropriate and efficient route for delivering counseling-based smoking cessation within the context of sustainability and scalability. These LHWs are generally recruited from the local catchment area of the health facilities and are already being deployed in HIV, sexual and reproductive health, and child welfare clinics. LHWs provide basic psychological counselling for testing, prevention, and medication adherence and serve as peer navigators. Once trained, they can deliver brief interventions as needed within the HIV clinics they are already stationed in.

Given the critical need for information about the up-front expenses required to implement smoking cessation services in Botswana, we plan to conduct a preliminary cost effectiveness analysis to evaluate the incremental net monetary benefit at 24 weeks of follow-up to compare the value of BSMART to an enhanced standard of care. We have also planned for the sustainability of the BSMART intervention. We have actively engaged diverse constituents who have worked together with the research team to select the intervention, develop the implementation design, and craft the dissemination plan. Pre-engagement activities have prepared the stakeholders and research team for meaningful engagement throughout the study. The strategic engagement of stakeholders in this study is intended also to develop their capacities to continually improve the implementation of all study activities and eventually promote the continuity of these beyond the project life. The Stakeholder Advisory and Practitioner Engagement groups will monitor target enrollment milestones, assist with developing lay descriptions of the study, and inform outreach efforts of the study to encourage diverse patient participation. Patient and stakeholder engagement helps to assure that intervention effects of treatment will have face validity with patients. By bringing patient, stakeholder, and practitioner’ perspectives into the dialogue, patient partners will help to enlighten researchers regarding potential alternative explanations or interpretations. This process will lead to appropriate and culturally sensitive translation and dissemination of results. The interpretation and translation processes help to document which results are easy or difficult to understand and guide how best to bring the results to patients and other decision makers.

We, therefore, have developed a simple, culturally adaptable intervention, based on an existing evidenced-based approach which will be delivered by lay health workers, followed by referral to treatment with varenicline prescribed and monitored by nurse prescribers in a network of outpatient HIV care. This study will not only assess the use of a brief intervention with a pharmacological component integrated into a well-funded HIV care system in a MIC but will also assess smoking cessation among PLWH and the capacities and coalitions of facilities required to maintain the integrated intervention. In addition, we have planned for the sustainability of the BSMART intervention by actively engaging key stakeholders, building the capacity for delivery and scale up of the intervention, and integrating this into a well-funded HIV care network.

### Supplementary Information

**Supplementary Material 1.**

## Data Availability

Not applicable.

## Abbreviations

ABLE: Accelerating Botswana through the Last Mile to Epidemic Control
BIC: Bayesian information criterion
BSMART: Botswana smoking abstinence reinforcement trial
ICER: Incremental cost-effectiveness ratio
INMB: Incremental net monitoring benefit
LHW: Lay health worker
MIC: Middle-income country
MOHW: Ministry of Health and Wellness
NB: Net benefit
NCD: Non-communicable diseases
PLWH: People living with human immunodeficiency virus
POSA: Probabilistic one-way sensitivity analysis
PSAT: Program sustainability assessment tool
RE-AIM: Reach effectiveness adoption implementation and maintenance framework
SBIRT: Screening, brief intervention, and referral to treatment
SOC: Standard of care
