# Supplementary material for: Design and rationale of the Botswana Smoking Abstinence Reinforcement Trial: a protocol for a stepped-wedge cluster randomized trial
Source: Implement Sci Commun. 2024 May 8;5:53. doi: 10.1186/s43058-024-00588-7 (PMC11077839; doi:10.1186/s43058-024-00588-7)
Supplement: Supplementary file 1 — Supplementary Material 1. [file 43058_2024_588_MOESM1_ESM.zip › Appendix A_BSMART Data Collection ToolsR0.docx]

Appendix A: BSMART Data Collection Tools

1. Research Consent Form – Control

2. Research Consent Form – Intervention Phase

3. Research Consent Form – Focus Group Discussion with LHWs

4. Research Consent Form – Focus Group Discussion with Nurse Prescribers

5. Research Consent Form – Collection of cost data from LHWs and Nurse Prescribers

6. BSMART Screening tool

7. BSMART Demographics form

8. Intake Smoking History Questionnaire

9. Follow-up smoking Questionnaire

10. Stages of Change Algorithm

11. Readiness Ruler

12. Varenicline Indications and Uses

13. Exclusion Criteria for Varenicline Use

14. BSMART Medication Adherence Form

15. BSMART side-effects checklist

16. BSMART Contraception consent form

17. BSMART Prescription form

18. BSMART Quit day preparation

19. FGD Guide for LHWs

20. FGD Guide for NPDs

21. Semi-structured Interview (SSI) for participants who did not quit smoking

22. SSI for participants who quit and then resumed smoking

23. SSI for participants who have quit smoking

24. Standard of Care brochure

25. BSMART Fidelity Checklist
