# Supplementary material for: Design and rationale of the Botswana Smoking Abstinence Reinforcement Trial: a protocol for a stepped-wedge cluster randomized trial
Source: Implement Sci Commun. 2024 May 8;5:53. doi: 10.1186/s43058-024-00588-7 (PMC11077839; doi:10.1186/s43058-024-00588-7)
Supplement: Supplementary file 1 — Supplementary Material 1. [file 43058_2024_588_MOESM1_ESM.zip › BSMART DSMB Charter V2.0 26APR2023R0.pdf]

DSMB Charter

Study Title: Botswana Smoking Abstinence Reinforcement Trial (BSMART)
Clinical Trials: NCT05694637

Principal Investigators: Dr Manhattan Charurat, Dr Bontle Mbongwe, and Dr Seth Himelhoch

Co-PI's: Dr. Jessica Magidson, Dr. Carlo DiClemente, Dr. Ndwapi Ndwapi, Dr. Eberechukwu Onukwugha, Dr. Bornapate Nokomo, Dr. Dinah Ramaabya, Dr. Robert Selato, Dr. Roy Tapera, Dr. Florence Bada, Dr. Lillian Okui, Dr. Milton Montebatsi

Institutions: University of Maryland, Baltimore, University of Botswana, University of Kentucky, University of Maryland Baltimore County, University of Maryland, College Park, Botswana University of Maryland Health Initiative, and Maryland Global Initiatives Corporation Botswana,

Study Location: Botswana

I. Role of the DSMB

The Data and Safety Monitoring Board (DSMB) will act in a supporting capacity to the University of Maryland, Baltimore and Ministry of Health and Research Division in Botswana, and study PIs to monitor participant safety and study progress. The DSMB reviews the study protocol, progress reports, and unanticipated problems and makes recommendations based on their reviews.

II. DSMB Responsibilities

The DSMB responsibilities are to:

- Review and sign this charter signifying understanding of responsibilities.
- Meet prior to study commencement to review the protocol and study timelines (ie reporting, etc). To establish channels of communication within the study team and the DSMB.
- Following study commencement
    - Review monthly reports on operational performance that include updates and any adverse events (review and communication via email).

- Convene <u>every 6 months (semiannually)</u> to monitor protocol progress, which may include some or all of the following:
    - Monitor protocol compliance to include participant recruitment, accrual, and retention
    - Monitor adverse events, participant withdrawals, and compliance concerns/issues
    - Review risks vs. benefits
    - Review any protocol modifications that occurred between DSMB review periods
- <u>During the Intervention period, the DSMB will convene every 3 months</u> to review progress, updates, and any adverse events.

- Review study performance, make recommendations and assist in the resolution of problems reported by the Principal Investigator

- Make recommendations to the PI's concerning regular continuation or modification of the trial procedures based on review of the semiannual or during intervention every 3 months reports.

- Make recommendation for study pause or stop for reasons of patient safety, if necessary

- The DSMB will discharge itself from its responsibilities when the last participant completes the study.

III. Membership of the DSMB

The DSMB will consist of three members. Members will have no financial, scientific, or other conflict of interest with the study.

<u>DSMB Members include:</u>

Billy Tsima, MD, MSCE
Associate Professor, Department of Family Medicine and Public Health
Faculty of Medicine at the University of Botswana.

Christopher Welsh, MD
Associate Professor, Department of Psychiatry
University of Maryland School of Medicine
And

Medical Director – UMMC Substance Abuse Consultation services
Medical Director – UMMC Outpatient Addiction Treatment Services and
Medical Director – Maryland Center of Excellence on Problem Gambling

Damon Vidrine, DrPH, MS
Vice Chair, Department of Health Outcomes and Behavior
Interim Chair, Department of Health Outcomes and Behavior
Department Chair: Department of Health Outcomes and Behavior
Moffitt Cancer Center

The DSMB includes experts in or representatives of the fields of:

- relevant clinical expertise (behavioral medicine, substance abuse, biostatistics, HIV care, clinical research, and clinical trials),
- research methodology (including clinical trials)
- biostatistics, and
- human subjects protection

DSMB Chair Responsibilities:

1. Responsible for overseeing the meeting and developing the agenda in consultation with the Principal Investigators
2. Serves as the contact person for the DSMB
3. Ensures that those involved in the day-to-day management of the study are excluded from DSMB voting procedures
4. Discusses the DSMB recommendations with the funder and appropriate members of the project. This responsibility may be delegated to the co-chair.
5. Takes and maintains minutes from closed sessions of the DSMB teleconferences.

DSMB membership is for the duration of the study. If any members leave during the trial, the PI will appoint their replacement.

By signing this charter, members attest to the absence of conflict of interest. Should this status change, the member of the DSMB will disclose to fellow DSMB members any real or perceived conflicts of interest. The DSMB will then determine the appropriateness of the member continuing to serve on the board.

IV. Operations

- PIs will provide all DSMB members with a copy of the IRB approvals from the reviewing IRBs (UMB and Local Botswana) and approved study protocol for review. DSMB members may provide any written questions, recommendations on the protocol and procedures prior to study commencement.
- Any changes made to the protocol must be approved by both IRBs, after which the DSMB members will be provided with the updated protocol for their information.
- PIs will provide all DSMB members with a progress reporting template prior to study commencement. DSMB members may provide any written recommendations on modifying the reporting template.
- PIs will provide a completed report (using the agreed upon template) to the DSMB semiannually following study commencement. During intervention period, the reports will be provided every 3 months.

- Severe adverse events that are deemed probably or definitely related to study participation will be reported to the DSMB (relevant IRB/s) within one week of awareness of the SAE at the study site. SAEs not related to study participation will be reported in the semiannual progress reports or every 3 months during the intervention period.
- DSMB members may make recommendations on modifying study procedures following review of semiannual reports or SAEs. Changes to the procedures based on DSMB feedback will be submitted to the IRB for approval.
- Ad hoc conference calls to discuss the study protocol, study progress, DSMB feedback and recommendations, or any other study-related issues may be requested by any of the DSMB members or PIs at any time during the study. Communication will otherwise occur via email.
- The DSMB will not be responsible for conducting interim analysis.

Estimated time commitment: DSMB members acknowledge approximately 1-2 hours, which may be greater or less, depending on the enrollment numbers and SAEs.

Open Report: the study team should prepare and submit a report that includes:
1. Accrual/recruitment information- including the number of patients screened, enrolled, completed, withdrawn, and reasons for withdrawal, if any. Multi-site studies include distribution by site.
2. Characteristics of subjects enrolled: include gender, age, and ethnicity.
3. Study information- table with age, gender, smoking status, HIV status, etc.
4. Adverse events- include description, grade, expectedness, relatedness.
5. Compliance with protocol- include any protocol deviations.
6. Any other pertinent study related information.

Closed Report: Closed reports are available only to those attending the closed sessions of the DSMB meeting, should be provided by the study statistician, and should include:
1. Analyses of primary and secondary endpoints
2. Subgroup and adjusted analyses
3. Adverse events analyses
4. Analyses of lab data.

V. Meetings and Quorum

1. Due to the size of the DSMB all members must be present for meetings.
2. Unscheduled meetings may be requested by any party involved in the study.

VI. Confidentiality

- All materials, discussions and proceedings of the DSMB are completely confidential. Members and PIs are expected to maintain confidentiality.

Appendix 1: Example of information to include in presentation for Internal OPEN (PIs and Co-investigators in attendance) meeting DSMB review.
PI/designee will provide presentation of the below information to the data safety and monitoring board members

1. Study/Protocol Information (number, title, PI and Co-PIs, location, start date, ect)
2. Study Risk classification
3. Brief overview of your study (Outcomes)
4. Study timeline review
5. Eligibility, Current Enrollment, and Sample size
6. Data acquisition and analysis plans (no outcome measures discussed)
7. Safety Monitoring
    a. Anticipated or Unanticipated adverse events
    b. Reporting actions
    c. Steps to minimize risks/discomforts to subjects
    d. Study risk classification evaluation and any modifications (if needed)

PI and or study staff will be asked to leave the meeting. At this time, the DSMB members will open discussion. The members will then provide recommendation for continued accrual or follow up, report results, terminate or stop trial, and or close the trial.

Protocol: Title (HP-XXXXX)

Data and Safety Monitoring Board Recommendations
Internal DSMB Members:
Dr.
Dr.
Dr.

Date of Internal Review:

The report has been reviewed in detail by all internal DSMB members:

Recommendation:
Pause Study activities
Terminate Study due to patient safety
Continue enrollment and or Follow up

Name of protocol: NAME and Brief Description (Lay Summary)

PI: Contact information (signature block)

A. Project Synopsis

A.1 Project Objectives

A.2. Brief Statement of Purpose

A.3. Projected Timetable and Schedule

B. Protocol Updates since last DSMB or IRB approval

C. Enrollment numbers

  a. Enrollment/Retention
  b. Participants withdrawn and reason
  c. Unanticipated events or SAE
  d. Patient safety concerns

D. Any change to Risk level?

E. If International, any changes to the local regulations or laws that impact this study? Any changes to the BOMRA waiver for the drug used?

F. Data Collection, Review, Analysis

B.2. Summary of Protocol Modifications and Regulatory Updates

1. List any modifications to protocol submitted to IRB and define what the modifications were
2. Any events that occurred (adverse events, unanticipated events, note to file actions) and define when or if submitted to the IRB as a reportable new incident (RNI)

DSMB Certification Regarding Conflict of Interest, Confidentiality, and Non-Disclosure Form

I agree to be a part of the Data Safety Monitoring Board for the BSMART study. I understand and agree to all the terms and conditions outlined in the DSMB charter for the above-named study. I am aware of my responsibilities for maintaining the confidentiality of any non-public information that I receive or become aware of through this activity, and for avoiding using such information for my personal benefit, the benefit of my associates, or the benefit of organizations with which I am connected, or with which I have a financial involvement. I have no conflicts of interest to disclose that make me ineligible to sit on this committee. I agree that in the event that the above may change during my tenure as a member of the DSMB, I will disclose and discuss the risk with the Sponsor/ Principal Investigators. Upon discovery of a risk, I will sign a new Conflict of Interest and Disclosure Statement form, and will include a description of the conflict. This includes the discovery that an organization with which I am affiliated meets the criteria for a conflict of interest.

I agree with the above statement

Name and Signature Date
