# Supplementary material for: Design and rationale of the Botswana Smoking Abstinence Reinforcement Trial: a protocol for a stepped-wedge cluster randomized trial
Source: Implement Sci Commun. 2024 May 8;5:53. doi: 10.1186/s43058-024-00588-7 (PMC11077839; doi:10.1186/s43058-024-00588-7)
Supplement: Supplementary file 1 — Supplementary Material 1. [file 43058_2024_588_MOESM1_ESM.zip › BSMART Initial UMB IRB approvalR0.pdf]

University of Maryland, Baltimore
Institutional Review Board (IRB)
Phone: (410) 706-5037
Fax: (410) 706-4189
Email: hrpo@umaryland.edu

APPROVAL OF RESEARCH NOTIFICATION

OF NOTE: The Principal Investigator should review the University of Maryland Baltimore criteria for performing research during the current COVID-19 pandemic emergency. Understand that IRB approval of this research does not suggest that performance of this research under current guidelines is allowed. Failure to comply with the UMB President's directives would be considered non-compliance. The UMB Research directives can be found at https://www.umaryland.edu/coronavirus/ . If you need clarification or guidance please call the Human Research Protections Office at 410-706-5037.

Date: January 19, 2023

To: Manhattan Charurat
RE: HP-00102995
Type of Submission: Initial Review
Type of IRB Review: Full Board

Approval for this project is valid from 12/19/2022 to 12/18/2023

This is to certify that the University of Maryland, Baltimore (UMB) Institutional Review Board (IRB) approved the above referenced protocol entitled, "*Botswana Smoking and Abstinence Reinforcement Trial*".

The IRB made the following determinations regarding this submission:
- Written informed consent is required. Only the valid IRB-approved informed consent form(s) in CICERO can be used.

This study is approved to enroll 0 local participants.

This study is approved to enroll 750 worldwide participants.

Below is a list of the documents attached to your application that have been approved:
Eligibility Checklist for HP-00102995 v9-26-2022-1664204697769
Botswana Smoking Abstinence Reinforcement Trial: A Stepped Wedge Cluster Randomized Trial
BSMART Protocol v4_Clean version
NOA_BSMART study
Screen shot of data fields from Botswana EMR
Varenicline Package Insert
Telephone Interview Script
Focus Group Interview Guides
Follow-up smoking use questionnaire
Intake smoking questionnaire
DSMB Charter_BSMART
Informed Consent Forms

English FGD with LHW ICF _ Tracked Changes
Responses to UMB IRB Committee
English FGD with NPD _Tracked Changes
English Control Phase ICF _Clean
Appendix IX_Exclusion Criteria for Varenicline Use
English FGD with Participants ICF _ Tracked Changes
English Intervention Phase ICF _Clean
English SSI with LHW ICF _Tracked Changes
Setswana Control Phase ICF
English FGD for LHW ICF _Clean
English FGD with NPD ICF _Clean
Setswana Intervention Phase ICF
English FGD with Participants ICF _Clean
Setswana FGD with LHW ICF
Setswana FGD with NPD ICF
English SSI with LHW ICF _Clean
Setswana SSI with LHW ICF
English Control Phase ICF_ Tracked Changes
English Intervention Phase ICF_ Tracked Changes

In conducting this research you are required to follow the requirements listed in the INVESTIGATOR MANUAL. Investigators are reminded that the IRB must be notified of any changes in the study. In addition, the PI is responsible for ensuring prompt reporting to the IRB of proposed changes in a research activity, and for ensuring that such changes in approved research, during the period for which IRB approval has already been given, may not be initiated without IRB review and approval except when necessary to eliminate apparent immediate hazards to the subject (45 CFR 46.103(4)(iii)). The PI must also inform the IRB of any new and significant information that may impact a research participants' safety or willingness to continue in the study and any unanticipated problems involving risks to participants or others.

DHHS regulations at 45 CFR 46.109 (e) require that **continuing review** of research be conducted by the IRB at intervals appropriate to the degree of risk and **not less than once per year.** The regulations make **no provision for any grace period extending the conduct of the research beyond 12/18/2023.** You will receive continuing review email reminder notices prior to this date; however, it is your responsibility to submit your continuing review report in a timely manner to allow adequate time for substantive and meaningful IRB review and assure that this study is not conducted beyond **12/18/2023**. Investigators should submit continuing review reports in the electronic system at least six weeks prior to this date.

Research activity in which the VA Maryland Healthcare System (VAMHCS) is a recruitment site or in which VA resources (i.e., space, equipment, personnel, funding, data) are otherwise involved, must also be approved by the VAMHCS Research and Development Committee prior to initiation at the VAMHCS. Contact the VA Research Office at 410-605-7000 ext. 6568 for assistance.

The UMB IRB is organized and operated according to guidelines of the International Council on Harmonization, the United States Office for Human Research Protections and the United States Code of Federal Regulations and operates under Federal Wide Assurance No. FWA00007145.

If you have any questions about this review or questions, concerns, and/or suggestions regarding the Human Research Protection Program (HRPP), please do not hesitate to contact the Human Research Protections Office (HRPO) at (410) 706-5037 or HRPO@umaryland.edu.
