# Supplementary material for: Design and rationale of the Botswana Smoking Abstinence Reinforcement Trial: a protocol for a stepped-wedge cluster randomized trial
Source: Implement Sci Commun. 2024 May 8;5:53. doi: 10.1186/s43058-024-00588-7 (PMC11077839; doi:10.1186/s43058-024-00588-7)
Supplement: Supplementary file 1 — Supplementary Material 1. [file 43058_2024_588_MOESM1_ESM.zip › BSMART Study Protocol_v5.1_June 6 2023R0.pdf]

Title: Botswana Smoking Abstinence Reinforcement Trial (BSMART): A stepped wedge cluster randomized trial

Multiple Principal Investigators:

Dr. Manhattan Charurat, MHS, PhD
Institute of Human Virology
University of Maryland, Baltimore
Baltimore, Maryland 21201-1508

Dr. Bontle Mbongwe, MSc, PhD
University of Botswana
Plot 4775 Notwane Rd
Gaborone, Botswana

Dr. Seth Himelhoch MD, MPH
University of Kentucky
245 Fountain Court
Lexington, Kentucky 40509

Co-Investigators

**Name**	**Role**	**Institutional Affiliation**
Dr. Jessica Magidson	Co-Investigator	University of Maryland, College Park
Dr. Carlo DiClemente	Co-Investigator	University of Maryland, Baltimore County
Dr. Ndwapi Ndwapi	Co-Investigator	Botswana University of Maryland Health Initiative
Dr. Eberechukwu Onukwugha	Co-Investigator	University of Maryland, Baltimore
Dr. Bornapate Nkomo	Co-investigator	Ministry of Health and Wellness, Botswana
Dr. Dinah Ramaabya	Co-investigator	Ministry of Health and Wellness, Botswana
Dr. Robert Selato	Co-investigator	National AIDS and Health Promotion Agency
Dr. Roy Tapera	Co-investigator	University of Botswana, Gaborone, Botswana
Dr. Florence Bada	Co-Investigator	University of Maryland, Baltimore
Dr. Lilian Okui	Co-investigator	Maryland Global Initiatives Corporation, BW
Dr. Milton Montebatsi	Co-Investigator	Botswana University of Maryland Health Initiative

Version number: 5.1

Version date: June 06, 2023

Table of Contents

List of Abbreviations 6

Protocol Summary 8

Introduction 9

Background 9

Rationale 11

Approach 14

Innovation 14

Methods 15

Study Design 15

Study Population 16

Study Treatment/ Intervention 17

Retention 18

Criteria for Stopping 18

Study Procedures 19

1. Adaptation of SBIRT 19

2. Site Integration and training for SBIRT 19

3. Establishing an Implementation Governance Structure for BSMART 20

4. **Qualitative Research** 22

5. **Training of LHWs, Nurse dispensers, and research assistants** 22

6. **Constituting consultation groups** 24

7. **Participant sampling and recruitment**: 24

Randomization methods 24

Participant Timelines - Control Phase 26

Informed consent 26

Enrollment in BSMART study 26

Follow-up 26

Participant Timeline - Intervention Phase 27

Informed consent 27

Enrollment in BSMART study 28

Provision of SBIRT by LHW case managers 29

Evaluation by nurse practitioners prior to varenicline use 29

Provision of varenicline 29

Monitoring for adverse events 29
Retention 30
Process and Outcome Measures 31
1. Clinical Outcomes........ 31
2. Measures to assess the Adoption and Implementation of SBIRT 31
3. Measures to assess if the BSMART intervention is maintained as part of routine practice over time across HIV care facilities 32
4. Measures to determine the preliminary cost-effectiveness of BSMART 32
Estimating process and outcome measures 33
Data Collection 34
Clinical Data 34
Power and Sample size 36
Statistical analysis 37
Statistical Analysis for Study Objective 1. 37
Statistical analysis for Study Objective 2 38
Statistical Analysis for Study Objective 3 39
Statistical analysis for study Objective 4. 39
Study timeline 41
Ethics and Protection of Human Subjects 43
Risks to Human Subjects 43
Adequacy of Protection Against Risks 47
Potential Benefits of the Proposed Research to Research Participants and Others 49
Importance of Knowledge to be Gained 50
Data and safety monitoring plan 51
Confidentiality / Data Management and Security 51
Protocol Compliance 51
Reporting Procedures........ 52
Data Safety and Monitoring Board (DSMB) 53
Data Collection and Storage 54
Data Safety and Security 54
Ensuring Data Quality 54
Dissemination Plan........ 56
References 58

Appendix A: Informed Consent Forms 62
Appendix A1: Research Consent Form – Control Phase 62
Appendix A2: Research Consent Form – Intervention Phase 68
Appendix A3: Research Consent Form- Focus Group Discussion with Lay Health Workers 77
Appendix A4: Research Consent Form - Focus Group Discussion with Nurse Prescribers/ Dispensers. 84
Appendix A5: Research Consent Form - Collection of cost data from LHWs and NPDs 91
Appendix B: Baseline, Smoking Questionnaires and Readiness Tools 97
Appendix BI: BSMART Screening Tool 97
Appendix B2: BSMART Demographics Form 98
Appendix B3: Intake Smoking Questionnaire 99
Appendix B4: Follow-Up Smoking Questionnaire 110
Appendix B5: Stages of Change Algorithm 114
Appendix B6: Readiness Ruler 116
Appendix C: Tools for Varenicline Use 117
Appendix C1: Varenicline Indications and Uses 117
Appendix C2: Common Terminology Criteria for adverse Events and Common Toxicity Criteria 119
Appendix C3: Exclusion Criteria for Varenicline Use 120
Appendix C4: BSMART Medication Adherence Form 121
Appendix C5: BSMART Side-effects Checklist 122
Appendix C6: BSMART Contraception Consent Form 125
Appendix C7: BSMART Prescription Form 126
Appendix C8: BSMART quit day preparation 127
Appendix D: Focus Group Guides 129
Appendix D1: FGD Guide for Lay Health Workers 129
Appendix D2: Focus Group Guide for Nurse Prescribers 137
Appendix E: Semi-structured Interview Questionnaires 144
Appendix E1: SSI for participants who did not quit smoking 144
Appendix E2: SSIs for participants who quit and then resumed smoking 151
Appendix E3: SSIs for participants who have quit smoking 157
Appendix F: Activity Log: 163
Appendix G: Program Sustainability Tool 164
Appendix H: Tools for Collecting Fixed and Variable Costs 165
Appendix I: Standard of Care Brochure 166

Appendix J: BoMRA Waiver 168

List of Abbreviations

ABLE	Accelerating Botswana through the Last Mile to Epidemic Control
BIC	Bayesian information criterion
BSMART	Botswana smoking abstinence and reinforcement trial
BUMMHI	Botswana University of Maryland School of Medicine Health Initiative
DSMB	Data safety and monitoring board
EMR	Electronic medical records
ERIC	Expert recommendation for implementing change
FWA	Federal-wide assurance
HRDC	Health research development committee
ICER	Incremental cost-effectiveness ratio
INMB	Incremental net monitoring benefit
IPMS	Integrated patient management system
IRB	Institutional review board
LHW	Lay health workers
LMIC	Lower middle-income countries
MOHW	Ministry of Health and Wellness
NADM	Non-AIDS-defining malignancy
NAHPA	National AIDS and health promotion agency
NB	Net benefit
NPD	Nurse prescriber/ dispenser
OHRP	Office for human research protections
PIMS	Patient information management system
PLWH	People living with human immunodeficiency virus
POSA	Probabilistic one-way sensitivity analysis
PSAT	Program sustainability assessment tool
RE-AIM	Reach effectiveness adoption implementation and maintenance framework
SAE	Serious adverse events

SBIRT	Screening, brief intervention, and referral to treatment
SOC	Standard of care
SPP	Suicide prevention plan
SSA	sub-Saharan Africa
UMB	University of Maryland Baltimore

Protocol Summary

Tobacco use is highly prevalent among people living with HIV/AIDS (PLWH), especially in southern Africa where HIV is most heavily concentrated. Among PLWH, tobacco use impacts HIV-related co-morbidities and is also the leading cause of premature mortality from non-HIV related malignancies such as lung cancer which account for 20% of the cancer burden. Integrating an evidence-based intervention, such as Screening, Brief Intervention and Referral to Treatment (SBIRT) into a HIV care system presents an important opportunity to establish and evaluate a modifiable cancer prevention strategy into a low-middle-income country (LMIC) setting where both lay health workers (LHW) and non-physician clinicians are widely used. Botswana, where the University of Maryland Baltimore (UMB) has worked since 2015, oversees a wide network of HIV care clinics for its citizens. Demographic Health Surveys from sub-Saharan Africa show that smoking prevalence among PLWH ranges between 12.5-44.3%. Yet, based on our pilot data, the system of care is highly unprepared to meet the challenge of integrating evidence-based smoking cessation treatment into routine HIV care.

The Government of Botswana wants more to be done to assist its citizens in smoking cessation. To meet this challenge, the **B**otswana **Sm**oking **A**bstinence **R**einforcement **T**rial (**BSMART**) proposes to use a Type 2 hybrid effectiveness-implementation study design to evaluate the effectiveness and implementation of a well-established Screening, brief intervention, and referral to treatment (SBIRT) intervention consisting of the 5”A”s, (Ask, Advise, Assess, Assist, Arrange) delivered by trained LHW case managers, followed by referral to treatment with varenicline (a medication demonstrated to be efficacious for smoking cessation among PLWH[1,2] prescribed and monitored by trained nurse prescribers/dispensers, in the network of outpatient HIV care facilities in Botswana.

The study objectives guided by the RE-AIM Framework and informed by an Implementation Governance Structure are to:

1) Assess ***Reach*** and ***Effectiveness*** of BSMART;

2) Assess the ***Adoption*** and ***Implementation*** indexed by quality and consistency of intervention delivery;

3) Assess whether the intervention becomes ***Maintained*** as part of routine practices; and

4) Determine the preliminary cost-effectiveness of BSMART.

Introduction

Background

Cancer epidemiology among people living with HIV (PLWH) has shifted since the introduction of antiretroviral therapy (ART). Over the last 10 years, rates of AIDS-defining malignancies, including Kaposi sarcoma, non-Hodgkin's lymphoma, and cervical cancer have decreased, while rates of many non-AIDS-defining malignancies (NADMs) substantially increased[3–5]. Lung cancer is the most common NADM among PLWH, accounting for 20% of the cancer burden[6,7]. Lung cancer incidence has been consistently reported as greater in PLWH than persons living without HIV[4] with an excess risk of 52% among PLWH compared with the general population[8–10]. Lung cancer control and prevention is an area of major clinical and public health interest for PLWH.

Among PLWH, tobacco use has been consistently shown to impact HIV-related co-morbidities and has also been identified as the leading cause of premature mortality from non-HIV related cancers. Smoking continues to be a major risk factor for lung cancer in the HIV infected population[7]. The incidence of lung cancer is almost 3-4-fold among PLWH tobacco smokers than nonsmokers and develops at a younger age in PLWH compared to the general population. Most lung cancers in HIV-infected patients occur in current or former heavy smokers[11]. A modeling analysis looking at the risk of lung cancer death due to smoking among PLWH showed that those who continued to smoke were six to 13 times more likely to die from lung cancer than from AIDS-related causes[12]. In our view, shared by others in the field,[13–15] the single greatest health behavior change that could improve mortality is to assist smokers living with HIV/AIDS to quit smoking.

Tobacco consumption in SSA is rising as tobacco companies increasingly target this region. Evidence indicates that the highest proportion of tobacco smokers are clustered in Southern African countries – Botswana, Lesotho, and South Africa (Figure 1.). This is particularly noteworthy given that Southern Africa is also the region with the highest prevalence and incidence of HIV globally (Figure 2).

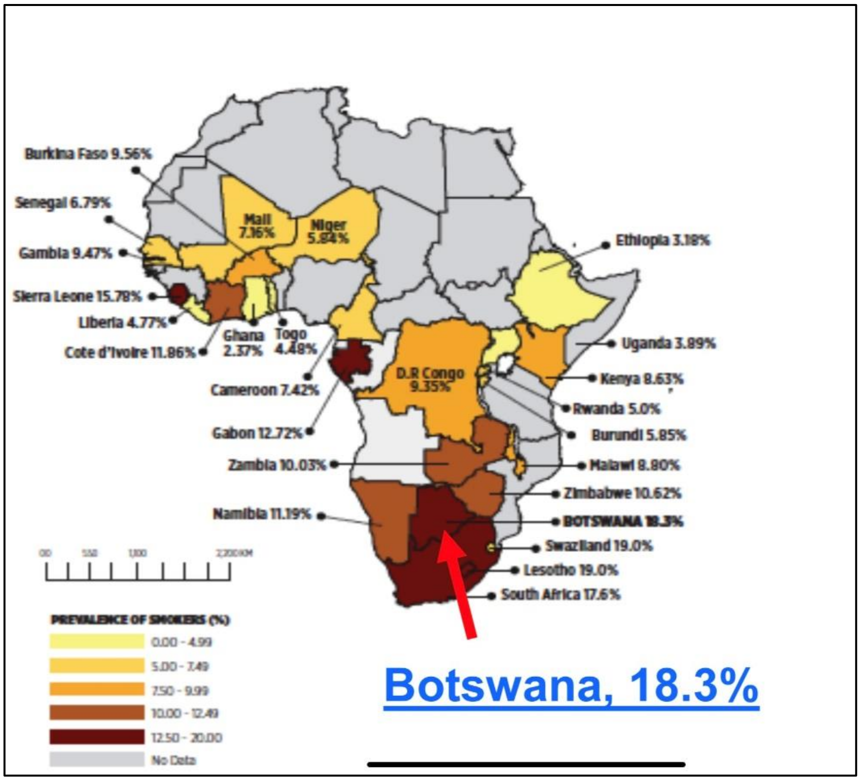

Figure 1: Prevalence of smoking in Botswana

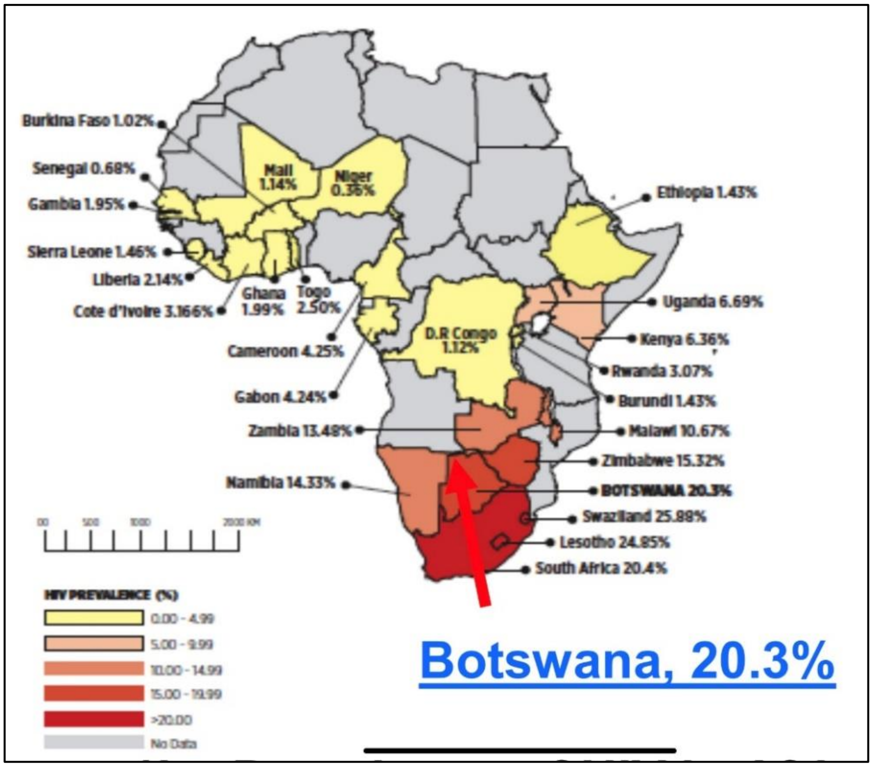

Figure 2: Prevalence of HIV in Africa

The Parliament of Botswana had in August 2021, passed a Tobacco Control Bill, which is currently awaiting signature by the President of Botswana. This Bill, which is sponsored by the Minister of Health and Wellness aims to discourage smoking initiation, encourage quitting, and reduce tobacco consumption overall to address prevention and control of non-communicable diseases such as lung cancer. The Global Adult Tobacco Survey conducted in 2017 shows that while the majority of smokers in Botswana made an attempt to quit in the past 12 months, only 7% of smokers were able to successfully quit. The Government of Botswana wants more to be done to assist its citizens in smoking cessation.

The average years of life lost by PLWH smokers compared with HIV-positive non-smokers have been estimated as 12·3 years, which is more than twice the number of years lost by HIV infection alone[16]. Botswana is particularly vulnerable because of its near transition from a Stage 1 tobacco smoking epidemic (prevalence of cigarette smoking of cigarette smoking <20%) to a Stage 2 (rapid increase in male smoking and prevalence beyond 20%). It is expected to face the largest growth in tobacco consumption in the world.[17] If interventions for PLWH can be designed and implemented at scale, a significant impact on lung cancer control can be achieved.

Rationale

The Botswanan Government has recognized that smokers, especially those who are living with HIV, need additional assistance beyond the standard smoking cessation services. The Ministry of Health and Wellness is therefore calling for implementation science that uses proven evidence-based interventions for smoking cessation that can be deployed at scale for HIV care facilities at an acceptable cost.

HIV care settings provide opportunities for early intervention with patients who smoke. The Ministry of Health and Wellness has expressed enthusiasm for leveraging the use of lay health workers in smoking cessation programs. Lay health workers are considered critical in filling the human resource constraints in Botswana, and thus the most appropriate and efficient route of delivery of counseling-based smoking cessation within the context of sustainability and scalability. In addition, there is a growing body of evidence supporting the effectiveness of the SBIRT (Screening and brief intervention, and Referral to treatment) intervention in a variety of settings[18–20]. The use of SBIRT has been found to not only increase the likelihood of making a tobacco quit attempt among patients who received a brief intervention, but it has also been strongly and consistently associated with increased satisfaction with care provided[20]. In a

review of literature examining the use of SBIRT for tobacco cessation, studies suggest that even low-intensity SBIRT may prompt quit attempts, decrease cigarette use, and facilitate quitting, if offered routinely[21]. We have adapted the SBIRT approach by creating trainings that would allow lay health workers to deliver the 5 "A"s tailored to the unique needs of PLWH. The likelihood of a successful quit attempt is increased if counseling is provided along with pharmacologic therapy[22,23] especially for individuals who smoke and are willing to quit.

We plan to use varenicline, a high-affinity partial agonist for the nicotinic acetylcholine receptor subtype, which has been shown to be more efficacious than nicotine replacement therapy or bupropion in a large pharmacotherapy smoking cessation trial[24]. The rationale for choosing varenicline is based on many factors including availability, cost, and efficacy. Currently, smoking cessation pharmacological interventions available in Botswana are limited to nicotine replacement therapy (NRT) which can be purchased over the counter (cost: $28/month). and varenicline which requires a prescription (cost: $50/month). Of note, a pack of cigarettes costs about $7 (or about $210/month [one pack per day]) which makes smoking almost eight times as expensive as NRT. Additionally, some participants may prefer not to use NRT given past experience of side effects, lack of effect or strong preference not to use it which may limit their interest in participating in the study or following through with study procedures. Finally, the effects of NRT have been mixed; though two randomized control trials demonstrated efficacy of NRT both alone and combined with behavioral therapy[25,26], two randomized control trials showed no effect of NRT on cessation rates. Varenicline, however, consistently demonstrated efficacy in two randomized control trials among PLWH[1].

Supported by the Botswanan Ministry of Health and Wellness and with sustainability and scalability in mind, the Botswana Smoking Abstinence Reinforcement Trial (BSMART) will use a Type 2 hybrid effectiveness-implementation study design, informed by the RE-AIM framework to evaluate the effectiveness, implementation and preliminary cost-effectiveness of a well-established SBIRT intervention (consisting of the 5"A"s) delivered by trained LHW case managers, followed by referral to treatment with varenicline prescribed and monitored by trained clinicians in a network of outpatient HIV care. The study objectives are as follows:

1. To assess the *Reach* and *Effectiveness* of BSMART.

   i. Reach is defined as the proportion of PLWH who agree to participate and screen in the intervention program,

ii. Effectiveness is defined as a 7-day biochemically verified point prevalence abstinence from combustible tobacco products at 6 months using CO Check+. Our sample size (n=750 participants) has sufficient power to show at least 8% increase in a clinical effectiveness endpoint in the implementation group relative to standard of care (SOC) in the control group.

2. To assess the *Adoption* and *Implementation* of BSMART indexed by quality and consistency of intervention delivery.
   i. identify how BSMART can be tailored to the network of HIV clinics in Botswana for adoption and implementation fidelity,
   ii. foster comparison across clinical sites, and
   iii. inform the development of activity logs to track implementation strategies.

3. To assess whether the intervention becomes *Maintained* as part of routine practices over time across HIV care facilities.
   i. determine the extent to which the core components of BSMART and implementation strategies continue to be delivered and are maintained over time with fidelity and
   ii. using the Program Sustainability Assessment Tool (PSAT), determine how organizational capacity and infrastructure to deliver BSMART are maintained, including partnerships, networks, and coalitions.
4. To determine the preliminary cost-effectiveness of BSMART - the incremental net monetary benefit at 6 months of follow-up in order to compare the value of BSMART to standard of care (SOC).

Hypothesis:

The BSMART intervention (SBIRT and varenicline) will

i. Increase the proportion of smokers able to quit smoking
ii. Increase the number of attempts to quit smoking
iii. Increase the length of abstinence in failed quit attempts
iv. Decrease the number of cigarettes smoked every day
v. Decrease the number of days of using combustible products within the past month
vi. Decrease the number of days of using combustible products within the past three months in people exposed to the intervention as compared to people in the control phase

Potential Impact: Addressing the syndemic between tobacco use and HIV infection in a coordinated manner aligns with the NIH HIV/AIDS priorities for effective cancer control among PLWH in a high HIV prevalence setting. This study is significant because it integrates smoking cessation interventions, which are lacking in sub-Saharan Africa (SSA), into a real-world HIV care system that has been underpinned by a robust PEPFAR/CDC program and leverages long-standing partnerships with Ministry of Health and Wellness and the University of Botswana's Anti-tobacco Network for population, health system, and policy level impact.

Approach

The traditional research pipeline that is a phased approach to moving interventions from efficacy trials to the real world can take a long time and inhibit the application of research knowledge in a real-world setting. To speed this process, BSMART proposes to use a Type 2 hybrid effectiveness-implementation study design to simultaneously test effectiveness and implementation strategies of existing and evidence-based smoking cessation interventions. Targeted adaptation of these interventions will be informed by our in-depth understanding of the local context and deep-rooted reach into existing HIV care infrastructure for effectiveness and supplemental qualitative data to identify barriers/facilitators in order to assess adoption and implementation factors. The durability/maintenance of the implementation will be examined, and cost-effectiveness to inform the MOHW and the Botswana National AIDS and health promotion agency (NAHPA) will be assessed.

Innovation

The tobacco epidemic is one of the largest public health threats the world has ever faced, killing more than 8 million people a year globally. Yet, interventions in LMIC have been left frustratingly behind. We also argue that the well-funded HIV care system presents a unique opportunity to integrate smoking cessation interventions and move tobacco control into the global conversation. The primary innovation of this study is focused on improving not only smoking cessation among PLWH but also the facilities' capacity and coalitions to maintain the integrated intervention. Post-intervention follow-up will help us understand durability of the implementation. This project is a rare example of a care team intervention focusing on LHWs and task-shifting in the LMIC context. The trial itself will precisely quantify key indices of implementation strategies while also providing effectiveness of interventions.

Methods

Study Design

We will utilize a stepped wedge cluster randomized trial to implement BSMART. BSMART will be sequentially rolled out to 15 participating HIV treatment and care facilities assigned in three steps (Table 1). Each step will provide data for a 12- month control/pre-implementation, a 12-month implementation, and a 12-month maintenance period. We will stratify Botswana's HIV treatment and care facilities into three levels of facilities. We will randomly assign each of the three levels of facilities to one of three study steps. Each step will comprise (i.e. have representation) from three levels of facilities, giving each level of facilities the same probability of beginning the intervention at any step). We choose this design to:

1) minimize the practical, logistical, and financial constraints associated with large-scale project implementation,

2) control for the effect of time, and

3) ensure that all HIV treatment and care practices will eventually offer the intervention and assess maintenance.

The study will screen approximately 6,900 HIV-infected patients and we expect to enroll 750 eligible participants, 375 of whom will participate in the pre-implementation phase and 375 of whom will participate in the implementation phase across three waves of implementation at five facilities each.

Table 1: Time Periods for Stepped Wedge Implementation

Steps	Total Number of Participants	Year 1	Year 2	Year 3	Year 4	Year 5
1	5 facilities, n=250 PLWH (1DH, 2 PHs, 2 clinics)	Control	Implementation	Maintenance		
2	5 facilities, n=250 PLWH (1DH, 2 PHs, 2 clinics)		Control	Implementation	Maintenance	
3	5 facilities, n=250 PLWH (1DH, 2 PHs, 2 clinics)			Control	Implementation	Maintenance

Study Population

Participants will be drawn from the population of HIV-infected patients receiving care at 15 selected health facilities that are part of the Accelerating Botswana through the Last Mile to Epidemic Control (ABLE) project. We plan to screen approximately 6,900 HIV-infected patients (230 PLWH per facility per year) for tobacco smoking and subsequently identify 750 patients who are interested in quitting into the trial, a total of 375 of whom will participate in the control period and 375 of whom will participate in the implementation period across three waves of implementation at five facilities each. Study participants will receive HIV care and treatment according to national standards.

Eligibility criteria: To maximize "real world" practice we attempted to err on the side of inclusivity in the study's eligibility criteria. We will apply the following inclusion criteria:

(1) HIV-infected,

(2) self-reported current daily smoker,

(3) age 18 years and older,

(4) engaged in HIV care as defined by being on ART for at least 6 months at one of 15 selected health facilities (or four reserve facilities), and

(5) willing/able to provide informed consent in English or Setswana.

We will apply the following exclusion criterion:

(1) pregnancy or nursing

We acknowledge that patients living with HIV/AIDS in real-world treatment settings use other substances in addition to tobacco and will often have some problems associated with that use. To limit this study to those who smoke tobacco only means tailoring the intervention to a small group that is not truly representative of the larger population of PLWH.

Study Treatment/ Intervention

Screening, Brief Intervention, and Referral to Treatment (SBIRT).

SBIRT is a comprehensive, integrated, public health approach to the delivery of early intervention and treatment services for persons with substance use disorders. The utility of SBIRT for all forms of tobacco use, especially smoking, has been endorsed by the U.S. Preventive Services Taskforce. We will use a SBIRT approach to promote smoking cessation using the 5 "A"s (Ask, Advise, Assess, Assist and Arrange for Follow-up) that has been used in multiple settings and found to be particularly effective with tobacco and alcohol-use disorders.

Screening and brief intervention: Trained LHW case managers will oversee the screening and brief intervention procedures (i.e., the 5"A"s). The first "A" begins the intervention with the LHW "Asking" eligible clinic clients about smoking. When a participant reports being a daily smoker, the LHW will link the participant to a research assistant in the clinic who will obtain informed consent and enroll the smoker in the trial. The participant will return to the LHW case manager who will proceed to the next 3 "A"s (Advise, Assess, Assist). These 3"A"s will constitute the brief intervention. The LHW will utilize a motivational enhancing conversation focusing on increasing insight and awareness regarding smoking, offering information and Advice and Assessing motivation toward behavioral change. For those participants who are motivated for treatment, a referral (Assist) will be made to a clinic nurse prescriber (NPD) for evaluation for treatment with varenicline. For those not ready to make a quit attempt, the LHW will encourage consideration of quitting and Arrange for a follow-up conversation. A culturally acceptable standardized form will be integrated into intake procedures within the HIV clinic that will allow case managers to document implementation and outcomes of using the 5"A"s.

Referral to treatment: Treatment with varenicline will be offered and provided to those motivated to quit. Smokers will initiate medication treatment with varenicline with a quit date scheduled for day 8 following the first study dose of the medication. They will meet with the study clinician at baseline who will provide medical clearance and sign off on prescription orders. All medication will be provided to participants by the study team. Participants will receive a weekly supply of medication for the first four weeks to ensure proper dosing and monitoring for adverse events. For the subsequent 8 weeks, participants will return every 4 weeks to receive the next month's supply of medication. Dosage adjustments will be permitted to control adverse effects throughout the trial. This will allow us to balance

internal validity with good clinical practice. The dosage of varenicline will be in accordance with package labeling.

Adverse events monitoring will be conducted by the study clinician each time a participant picks up their medication. The effectiveness of the referral process to specialized treatment is a strong measure of SBIRT success and involves a proactive and "warm handshake" between LHW case managers and clinicians to make sure the connection between PLWH and referrals are facilitated. LHW case managers will also provide cessation behavioral strategies, arrange follow-up check-ins to reinforce quitting for successful quitters, re-screen, and encourage cessation for those who do not succeed and return to earlier stages of change.

Standard of Care

The stepped-wedge design allows each site to serve as its own control. Based on our preliminary data, it appears that some but not all clinicians screen for smoking behaviors. Few clinicians provide brief interventions or referral to treatment. To ensure each site has a similar baseline at the beginning of the study, each site will receive a short-quit smoking brochure that each clinic can provide to patients who are identified as smokers. This approach is often used as a control condition for smoking cessation efficacy trials, and we believe it will be useful in our planned effectiveness trial as well.

Retention

We have maintained high viral load suppression and retention rates under the ABLE project. We will leverage the current approach and include additional strategies such as information leaflets about the importance of retention, appointment cards, motivational text messages, and phone calls (and home visits) to remind participants of appointments. All participants who miss study visits will be contacted.

Criteria for Stopping

Withdrawal will take place in the case of significant side effects or at a participant's request.

Psychiatric emergencies: If suicidal ideation or intent is observed, the study suicide prevention plan (SPP) will be implemented, and the participant will be withdrawn. The SPP consists of immediate psychiatric evaluation, use of a suicide prevention contract, provision of 24-hour access to a physician, and referral to emergency services.

Study Procedures

1. **Adaptation of SBIRT**

We will draw on the input of stakeholders such as the Anti-tobacco Network, and our experience working in the HIV care system in Botswana to integrate the use of LHWs. Nesting of the trial within ABLE allows our team including the front-line HIV care team to gain sufficient experience with the interventions. We will lead the formal training which will take 1 week and will emphasize interactive activities such as roleplaying. To ensure sufficient assimilation of training materials, LHW case managers must pass a post-training examination. The 5"A"s already has established theory-informed and evidence-supported methods and strategies to achieve behavior change. New training materials and revisions to the existing LHW case manager standard operating procedures will be piloted. We will hold a pre-implementation workshop with implementation leaders, LHW case managers, and NPDs to discuss the proposed integration of smoking cessation interventions into their scope of work. Training slide sets and materials will be reviewed. Feedback will be solicited on how to successfully adapt LHW case managers for smoking cessation interventions, and stakeholders will be provided with the opportunity to contribute. Engagement of these staff and key informants will ensure strong buy-in and robust sensitization on the outpatient service when we are ready to implement.

2. **Site Integration and training for SBIRT**

Based on our experience with SBIRT implementation, we identified the following areas for its integration into a clinical setting.

Site Engagement: The clinical director or administrator of each facility will be asked to sign a Memorandum of Understanding committing to the BSMART study implementation.

Care Team Staffing: Four implementation leaders at country level, and two LHW case managers and two nurse prescribers/dispensers, will be identified at each of the facilities, the majority of whom are already dedicated to PLWH-centered counseling and HIV treatment management. Implementation leaders will be trained to be trainers on motivational interviewing and the components of the BSMART intervention. Before each implementation period, the implementation leaders will facilitate a 3-day kick-off orientation for the BSMART study and train LHWs and NPDs from sites to be activated at a central location. They will also provide in-service training as needed. The study investigators will provide a wide array of resources e.g. training materials, implementation procedures, and host regular calls with the implementation leaders to support implementation efforts.

Clinical Skill Set: Training for all implementation leaders will be scheduled and led by Dr. DiClemente. The training will introduce the study and provide the background and orientation to the 5"A"s and its assessment form.

Medical Management: A similar training will be provided for implementation leaders who in turn will train all licensed clinicians eligible to prescribe varenicline. This training will be scheduled and led by Dr. Himelhoch. The training will provide the background and orientation to treatment with varenicline. A consultation group will be established to address questions clinicians may have when prescribing varenicline.

Workflow Processes: We will review how to integrate SBIRT into the care team. For example, we will have a LHW case manager in place at the outpatient clinics whose scope will be adapted to meet with the new and existing patients before the end of the clinic visit to introduce the study and conduct the 5"A"s. Other adaptations will include: 1) developing and piloting the new clinical workflow between LHW case manager and nurse prescriber/dispenser; 2) training the care team in implementing the new workflow; and 3) using monthly activity logs to track implementation strategies such as actions, methods, events, or efforts to promote adoption and implementation of SBIRT components.

3. Establishing an Implementation Governance Structure for BSMART

Guided by the Consortium for Cancer Implementation Science's Tools for Stakeholder and Community Engagement, we will establish an implementation governance structure that integrates the voices of stakeholders, practitioners, and researchers (Figure 3).

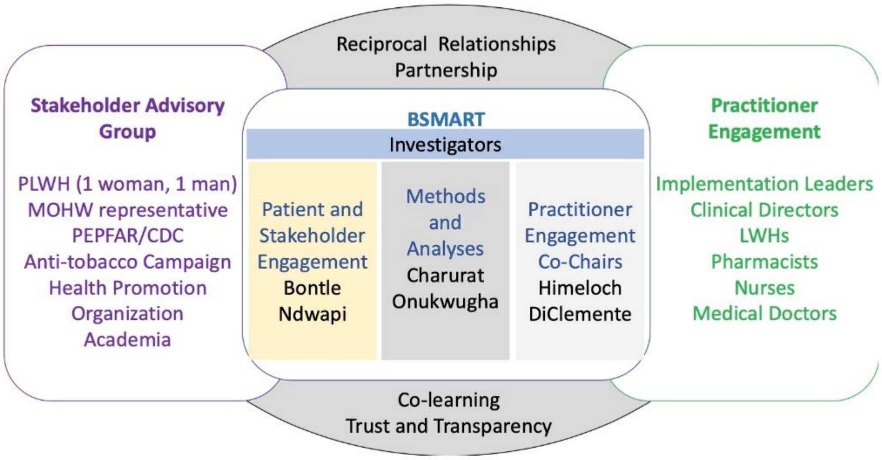

Figure 3. BSMART Implementation Governance Structure

We consider our Stakeholders key to our research; not just in the role of providing advice, but as integral partners in all of our efforts related to the development, implementation and dissemination of our BSMART program.

The membership of the Stakeholder Advisory Group reflects the diverse constituents who are invested in the successful implementation of the smoking cessation program within the context of HIV service delivery. Drs. Mbongwe and Ndwapi will co-lead the Stakeholder Advisory Group activities. Drs. Himelhoch and DiClemente will co-lead the Practitioner Engagement Group. The stakeholders have worked hand-in-hand as colleagues with the BSMART research team to select the intervention, develop the implementation design, and craft the dissemination plan. **Pre-engagement activities** during the proposal development prepare practitioners and stakeholders as well as researchers for meaningful engagement throughout the study. The shared goal of an equitable partnership means the creation of a conceptual framework for implementation and the **analysis plan** are designed to be meaningful for implementers and patients. These on-going focus groups meetings with our Stakeholder Advisory and Practitioner Engagement Groups should allow us to develop the conceptual framework with maximal input. For example, based on pre-engagement questionnaires with practitioners across HIV care clinics, we have included measures of competencies in delivering SBIRT.

The Stakeholder Advisory Group and Practitioner Engagement Core will monitor target enrollment milestones; assist with developing lay descriptions of the study; and inform outreach efforts to encourage diverse patient participation. Patient and stakeholder engagement helps to assure that intervention effects of treatment have face validity with patients. Part of monitoring the conduct of the project is contributing to the data analysis process and reviewing and interpreting results. By bringing patient, stakeholder, and practitioner' perspectives into the dialogue, patient partners will help to enlighten researchers regarding potential alternative explanations or interpretations. This process will lead to appropriate and culturally sensitive translation and dissemination of results. The interpretation and translation processes help to document which results are easy or difficult to understand and guide how best to bring the results to patients and other decision makers.

4. **Qualitative Research**

Pre-implementation – Two focus group discussions (one for nurse prescribers and one for LHW case managers) will be conducted as part of a two-day work-shop in which the SBIRT intervention will be introduced to LHW case managers and nurse prescribers who will be implementing the BSMART intervention in Step 1 sites. The focus will be their initial thoughts on SBIRT and how SBIRT can be modified to make it more culturally appropriate and feasible.

Post-Introduction of SBIRT

Focus Group Discussions -Focus group discussions will be conducted with Lay Health Worker Case Managers and Nurse Prescribers concerning challenges to implementing the intervention and its integration with HIV care. Focus group discussions are ideal for identifying normative understanding following the generation of multiple perspectives. The sample size for each study group category anticipates what is required to reach saturation on key themes but may be supplemented if necessary.

Semi-structured Interviews

During their final study visit at week 24, participants will be asked to participate in a semi-structured interview. For participants who were unable to quit smoking in the course of their participation in the study, the reasons for this will be explored. For participants who quit and resumed smoking, the factors that prevented them from staying smoke free will be explored. For participants who were successful in quitting, the factors that enabled them to quit successfully will be identified.

5. **Training of LHWs, Nurse dispensers, and research assistants**

Training of LHWs on standard of care

LHWs will undergo a two-day onsite training on providing standard of care. This will be provided to them by the study coordinator, the data manager, and the research assistant assigned to the site. They will also be trained on how to fill the screening tool.

Training of LHWs on SBIRT

LHWs will receive formal training on SBIRT which will take three days and will emphasize interactive activities such as roleplaying. To ensure sufficient assimilation of training materials, LHW case managers must pass a post-training examination. The 5”A”s already has established theory-informed and

evidence-supported methods and strategies to achieve behavior change. New training materials and revisions to the existing LHW case manager standard operating procedures will be piloted.

Training of Nurse prescribers/dispensers on varenicline use

Nurse prescribers will attend a three-day didactic and hands-on centralized training on the SBIRT intervention and how to integrate it into their current work at sites. They will receive specialized training on the treatment component of SBIRT – varenicline use. They will be taught how to screen participants for their eligibility to receive varenicline, how to prescribe it and how to monitor participant adherence and side-effects. They will also receive training on the tools they need to complete -the adherence tool and adverse events tool.

In-service training will be conducted for all licensed clinicians eligible to prescribe varenicline - the nurse prescribers/dispensers, which will be scheduled and led by Dr. Himelhoch but provided by Implementation leaders. The in-service will provide the background and orientation to treatment with varenicline. These in-service trainings will be repeated at each clinic (at different days and times) to accommodate diverse staff schedules.

Training of Research Assistants

Research Assistants will undergo a two-week training before they commence work on the BSMART study. They will be trained on the following:

- Research ethics training which includes the Collaborative IRB Training Initiative certification (CITI)
- Good clinical practice
- the study protocol, eligibility and consenting processes,
- how to provide the standard of care intervention,
- CO monitoring
- how to fill the required data collection tools using REDCap and
- how to administer semi-structured interviews to participants

They will attend the training sessions for LHWs and Nurse prescribers on SBIRT.

6. **Constituting consultation groups**

   Consultation groups will be established to address questions clinicians may have when prescribing varenicline.

7. **Participant sampling and recruitment**:

   Recruitment activities will begin at the HIV care services through introduction of the study by the LHW Case Managers. Tobacco use screening will occur within the context of HIV outpatient care. The LHW Case Manager will identify individuals who initially screen positive for tobacco use. The LHW case manager will apply the study eligibility criteria and participants found to be eligible for the study will be linked with the research assistant stationed in the HIV clinic and working closely with the LHW case manager. Individuals who present at the study clinic for screening and are eligible will complete the informed consent process on a first come, first serve basis prior to the completion of any study specific activities. We will ensure ethical conduct of the study through respecting and protecting the interests of all potential and enrolled participants, building trust between researchers and participants, and conducting the study under the principles of good clinical and laboratory practices. Informed consent will be administered in the participant's choice of English or Setswana language by trained research assistants.

Randomization methods

We have chosen 15 (and 4 reserved) high-volume (>1000 HIV-positive patients currently active and on ART) health facilities (See Figure 4) in the ABLE project for inclusion based on numbers of patients, accessibility to the research team, and willingness to collaborate. After a baseline period of 1 year, the BSMART intervention will be implemented in a step of five new randomly selected sites (1 district hospital, 2 primary hospitals, 2 primary care clinics) until it is eventually implemented in all 3 steps (15 sites). A computer-generated list containing facilities stratified by type will determine the order in which the sites receive the intervention. The randomization list will be maintained by a statistician who is not involved in the study, and the site allocation will remain concealed from the research team and clinical teams throughout the study and will be revealed for a given step only 1 month before the implementation of the intervention to allow for training.

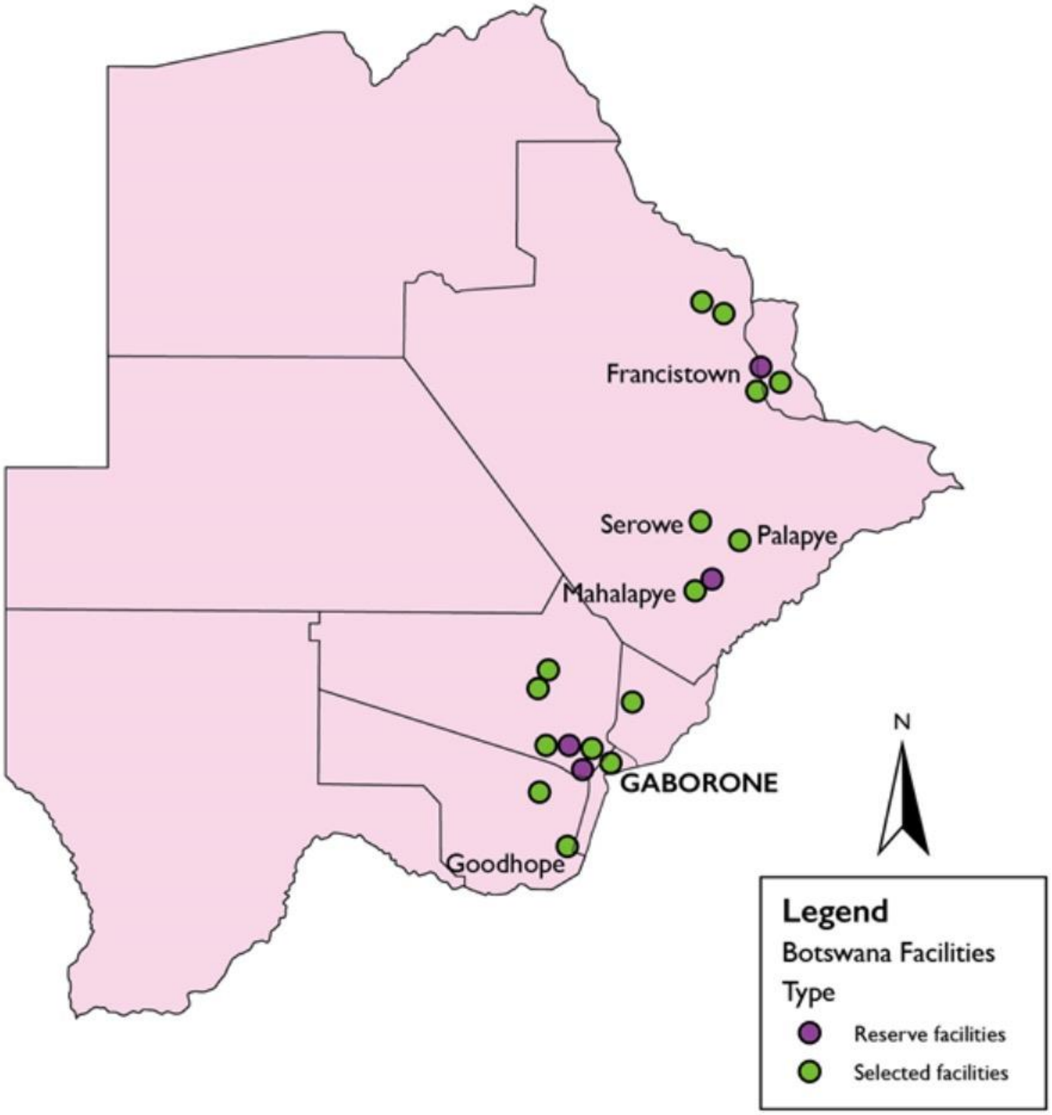

Figure 4: Selected and reserve facilities for BSMART project

Participant Timelines - Control Phase

Informed consent

<u>Study Participants (People living with HIV who smoke)</u>

Individuals who present at the study clinic will be screened for study eligibility. Those found to be eligible will be linked to a research assistant stationed within the same clinic who will lead them through the informed consent process and then offer enrollment to those interested in participating in the study. The informed consent process will be completed on a first come, first served basis prior to the completion of any study specific activities. A research assistant will provide a printed version of the signed informed consent to each participant as well as any study information the participant requests to have printed. The written consent will be provided in the participant's choice of English or Setswana and administered by trained research assistants. Research assistants will review the document with each participant in a private environment, respond to questions, and emphasize participant rights regarding research participation. Research assistants will use assessment tests in the consent process to ensure that the participants comprehend the study and are able to provide informed consent. The ability to provide informed consent is one of the inclusion criteria for this study. Participants will have the right to withdraw participation at any time, without interruption to their health and HIV care and treatment services rendered at the health facility.

Enrollment in BSMART study

At enrollment, research assistants will fill the demographic form, intake smoking history questionnaire and use the stages of change algorithm and readiness ruler to determine the individual's stage of change for quitting. Lay Health worker case managers will provide participants with the standard of care brochure which contains basic information on the risks of smoking and the need to stop smoking.

Follow-up

Research assistants will fill out follow-up smoking questionnaires at weeks 4, 12 and 24 after the patient receives the SOC brochure. Participants will be provided with breath CO measurements using CO Check+

(coVita) at baseline and then weeks 12 and 24 after enrollment. Participants will also be asked to respond to a semi-structured interview at week 24 that explores challenges to smoking cessation, barriers experienced by participants and reasons for success in those who were able to quit successfully.

Table 2: Participant Timeline- Control Phase

	Responsible person	1st clinic visit/ Baseline	Week 4	Week 12	Week 24
Screening of participants	LHW case manager	X			
Informed Consent	Research Assistant	X			
Enrollment in BSMART study	Research Assistant	X			
Intake Smoking Questionnaire	Research Assistant	X			
Receipt of SOC brochure	LHW case manager	X			
Follow-up smoking questionnaire	Research Assistant		X	X	X
Stages of change	Research Assistant	X	X	X	X
Breath CO	Research Assistant	X		X	X
Semi-structured Interview	Research Assistant				X

Participant Timeline - Intervention Phase

Informed consent

Study Participants (People living with HIV who smoke)

Individuals who present at the study clinic will be screened for study eligibility. Those found to be eligible will be linked to a research assistant stationed within the same clinic who will lead them through the informed consent process and then offer enrollment to those interested in participating in the study. The informed consent process will be completed on a first come, first serve basis prior to the

completion of any study specific activities. A research assistant will provide a printed version of the signed informed consent form to each participant as well as any study information the participant requests to have printed. The written consent will be provided in the participant's choice of English or Setswana and administered by trained research assistants. Research assistants will review the document with each participant in a private environment, respond to questions, and emphasize participant rights regarding research participation. Research assistants will use assessment tests in the consent process to ensure that participants understand the study and are able to provide informed consent. (The ability to provide informed consent is one of the inclusion criteria for this study). Participants will have the right to withdraw participation at any time, without interruption to their health and HIV care and treatment services rendered at the health facility.

<u>LHW case managers and nurse prescribers</u>

LHW case managers and nurse prescribers participating in the BSMART study will be asked to participate in Focus Group Discussions to obtain feedback on their experiences with integrating the BSMART intervention (Screening, brief intervention, and referral to treatment - SBIRT) into their work with a view to improve the process. A research assistant will lead them through the informed consent process and then offer enrollment to those interested in participating in the study. The research assistant will provide a printed version of the signed informed consent to each participant as well as any study information the participant requests to have printed. The written consent will be provided in the participant's choice of English or Setswana. Research assistants will review the document with each participant in a private environment, respond to questions, and emphasize participant rights regarding research participation. LHWs and Nurse prescribers have the right to decline participation or to withdraw at any time without affecting their employment or any of their rights or benefits.

Enrollment in BSMART study

At enrollment, research assistants will fill the demographic questionnaire, intake smoking history questionnaire and use the stages of change algorithm and readiness ruler to determine the individual's stage of change for quitting.

Provision of SBIRT by LHW case managers

Trained lay health workers will provide the SBIRT intervention (the 5"A"s) to enrolled participants in a private place in the HIV clinic. For participants not ready to make a quit attempt, the LHW will encourage consideration of quitting and arrange for a follow-up conversation.

Evaluation by nurse practitioners prior to varenicline use

Prior to being prescribed varenicline, eligible participants will be evaluated by a nurse prescriber to assess their suitability. Only eligible patients without any contra-indications to varenicline use will be prescribed varenicline.

Provision of varenicline

Participants referred for treatment with varenicline will be taught the correct use of varenicline by the nurse prescriber/ dispenser (NPD) at the site where the participant receives care. Participants will initiate medication treatment with varenicline for smoking cessation with a quit date scheduled for day 8 following the first study dose of the medication. Participants will meet with the NPD at baseline who will evaluate them, provide medical clearance and sign off on prescription orders if they meet the requirements for varenicline use (See Appendix IX). Varenicline will be prescribed in accordance with package labeling and provided to participants by the nurse prescriber/ dispenser. Participants will receive a weekly supply of medication for the first four weeks to ensure proper dosing and monitoring for adverse events. For the subsequent eight weeks, participants will return every 4 weeks to receive the next month's supply of medication. Dosage adjustments will be permitted in an effort to control adverse effects throughout the trial. This will allow us to balance internal validity with good clinical practice.

Monitoring for adverse events

Adverse event monitoring will be provided by the nurse prescriber/dispenser every time a participant picks up their medication.

Retention

We will leverage the current approach which utilizes lay health workers and expert clients to ensure retention in the study and include additional strategies such as information leaflets about the importance of retention, appointment cards, motivational text messages, and phone calls (and home visits) to remind participants of appointments. All participants who miss study visits will be contacted.

Table 3: Participant Timeline - Intervention Phase

	Responsible person	Baseline	W 1	W 2	W 3	W 4	W 5	W 6	W 7	W 8	W 12	W 24
Screening of participants	LHW case manager	X										
Informed Consent	Research Assistant	X										
Enrollment in BSMART study	Research Assistant	X										
Intake Smoking Questionnaire	Research Assistant	X										
Follow-up smoking questionnaire	Research Assistant					X					X	X
Stages of change	Research Assistant	X				X					X	X
SBIRT Intervention	LHW case manager	X										
Breath CO	Research Assistant	X									X	X
Adherence to varenicline	Nurse prescribers		X	X	X	X				X		
Adverse events monitoring	Nurse prescribers		X	X	X	X	X	X	X	X		
Semi-structured Interview												X

Process and Outcome Measures

1. Clinical Outcomes

Primary Outcome: The clinical effectiveness endpoint at six months is the 7-day point prevalence abstinence from combustible tobacco products validated primarily by breath CO < 6 ppm. The failure for this measure is any smoking (even a puff) during a 7-day window.

Secondary Outcomes:

- Quit attempts
- Length of successful quitting during each attempt
- Number of cigarettes currently smoked, and
- Number of days of using combustible tobacco products for at least 24 hours within the past month and the last 3 months.

2. **Measures to assess the Adoption and Implementation of SBIRT**

- Activity Logs: During the implementation period, implementation leaders and care teams will be sent a monthly email requesting that they complete an activity log, which will take 15–30 minutes to complete depending on the number of activities to report. The logs will collect information about implementation activities, intent, duration, and individuals involved. We will ask them to take an inclusive approach and include any activities they believe are related to implementation. We will also add a hypothetical example to the instructions and share a list of discrete implementation strategies. Each month, the research team will invest up to two hours to collect and combine the logs then follow-up with implementation leaders at sites to address their questions.
- Scripts from Focus Group discussions with LHWs
- Responses from SSIs
- SBIRT checklists

3. **Measures to assess if the BSMART intervention is maintained as part of routine practice over time across HIV care facilities**

RE-AIM measures will be re-assessed at 24 months after BSMART implementation to provide a standardized evaluation approach to understand whether the impact and implementation delivery are maintained and to shed light on where sustainability issues arise e.g. which clinic and populations the implementation is not reaching.

Integrating RE-AIM into quality improvement measures. If BSMART is successful, we will explore building the capacity of each site to use RE-AIM as a tool for institutionalizing quality improvement. Findings will be shared with each site at the end of the maintenance period during learning meetings, and Implementation Leaders will be encouraged to reflect on findings and plan next steps to improve implementation quality. Implementation lessons emerging from other sites will be shared to inform change efforts at other sites. This learning meeting approach will establish feedback loops that allow the intervention to be adapted to ongoing contextual changes.

Multi-level determinants of sustainability. To further identify constructs that are particularly important for sustainability, we will use the 40-item Program Sustainability Assessment Tool to capture measures of coalition, funding stability, partnerships, organizational capacity, program evaluation, program adaptation, communications, and strategic planning in a variety of public health programs during the implementation and maintenance period. Total sustainability score and domain-specific scores will be determined and compared between periods.

4. **Measures to determine the preliminary cost-effectiveness of BSMART**

Cost components and incremental costs. The cost-effectiveness of interventions will be compared based on the 7-day point prevalence abstinence at 6 months. The analysis will adopt a healthcare system perspective and calculate direct medical costs associated with the implementation of each strategy. Fixed costs include any set-up costs associated with the implementation of the interventions (e.g., supplies, information technology infrastructure, baseline staff training) and will be collected at week 0. Variable costs include ongoing staff or lay case manager and nurse contact time with study participants, and follow-up training (time and material costs). Variable costs will be collected at week 12 and quarterly thereafter. Time will be valued using prevailing wages that are applicable to the staff expertise. The calculation of the 6-month costs for the control and implementation phases will involve summing across each of the cost

components at the individual level to provide an estimate of the period cost (per individual and average period cost for each of the strategies. The incremental cost of the interventions will be defined as the difference in average costs when comparing the implementation period to control period. The incremental cost of implementing BSMART = ((‘component A’ cost per person x number of persons for implementation period) + (‘component B’ cost per person x number of persons for implementation period) +…) – ((‘component A’ cost per person x number of persons for control period) + (‘component B’ cost per person x number of persons for control period+ …)). The quarterly cost calculation accounts for the censoring of data due to loss to follow-up or administrative censoring using an inverse probability-weighting [27,28] approach.

Estimating process and outcome measures

All procedures will be conducted according to the local standards of routine clinical care. The following measures will be utilized:

1. **Baseline Demographic Information:** Baseline information will be abstracted from the HIV medical records and collected using a questionnaire. Baseline information will include demographics, medical history, laboratory values measured as part of HIV care (e.g. viral load, CD4), antiretroviral (ARV) history and ART treatment regimen (for current patients), and ART treatment start date. Because of high viral load suppression and high retention rate in this population, we will not be analyzing HIV treatment variables as mediators or outcomes.
2. **5”A”s (SBIRT) checklist:** This checklist will be completed by the LHW case managers and will serve to document the delivery of the intervention. A 10% random sample of the completed 5”A”s checklist will be reviewed by Dr. Magidson during the time that a site is engaged in the intervention to ensure fidelity to the study intervention.
3. **Intake Smoking History Questionnaire** will measure length of smoking, use of other tobacco products, quit attempts, length of longest quit attempt, and smokers in the immediate living environment.
4. **Follow-up Smoking Use Questionnaire** will measure 7-day and 30-day point prevalence abstinence, number of cigarettes smoked (if not abstinent), stage of change and readiness to quit (if not abstinent), length of longest period of abstinence since last evaluation, use of varenicline, side effects, reasons for using or not using varenicline, and length of varenicline use.

5. **Stages of Change Algorithm**[29]and the Readiness Ruler[30], will be used at baseline and follow-up to determine each individual's stage of change for quitting tobacco.
6. **Smoking Abstinence**: Abstinence will be based on self-reported 7-day point prevalence abstinence confirmed by expired CO<6 ppm. Abstinence criteria must be met in participant Month 6 or at an earlier study week and maintained through participant Month 6 to be considered a responder. We will use coVita CO Smokerlyzer, a single-breath point-of-care tool designed specifically for use in smoking cessation programs and clinics.

For those who agree to be treated with varenicline:

7. **Adherence to Medication**[31]: Pill and counseling adherence will be assessed at Weeks 0, 1, 2, 4, 8, and 12. Pill adherence will be tracked by the following question at each medication visit: How many days in the last week did you take at least one of your study pills[32]. Pill adherence will be defined by participant self-report of taking six or seven pills at week eight which coincides with their last receipt of varenicline.
8. **Adverse Event Monitoring** (Baseline, weekly for 4 weeks then every 4 weeks until week 12): Adverse events will be monitored at each study contact and classified and graded using the Common Terminology Criteria for Adverse Events and Common Toxicity Criteria provided by the National Cancer Institute. Our protocol for responding to Serious Adverse Events is summarized in the Human Subjects section.
   The 5"A"s (SBIRT) Checklist and the Intake Smoking History Questionnaire will be administered at baseline for PLWH newly enrolling to HIV treatment and those currently on HIV treatment. A Follow-up Smoking Use Algorithm will be administered at baseline and at 4-, 12-, and 24-weeks of follow-up. The CO measurement will be obtained at baseline, 12 and 24 weeks of patient follow-up using coVita CO Smokerlyzer .

Data Collection

Clinical Data

Data will be collected directly using tablets with REDCap Mobile Device Applications. The study team will work with the ABLE program staff to electronically extract all clinical and laboratory measurements longitudinally from the Integrated Patient Management System (IPMS) and the Patient Information

Management System (PIMS); the two main EMR systems used in Botswana and supported by BUMMHI, UMB's affiliate organization and BSMART study partner. Based on our experience, we anticipate high data quality as part of the ABLE program. All study sites will be connected by telephone and e-mail. Data will be extracted directly into a REDCap database (hosted by BUMMHI) by study personnel and immediately available for viewing by the study coordinator and data manager. Data checks will be put in place to verify completeness for each site (against IPMS, PIMS) and to check for data errors, using modifications of automated checks. Computer programmers in Botswana and Baltimore will evaluate the database and check for inconsistencies and missing data on an ongoing basis, and queries will be directed back to each site to maintain data quality.

Qualitative Data

	Data Collector	Participants	Timing
Telephone Interview	Investigators/ Research Assistants	Implementation Leaders	3-6 months into intervention phase
Focus Group Discussions	Investigators/ Research Assistants	Lay Health Workers Nurse Prescribers	Month 3 of intervention phase
Semi-structured Interviews	Investigators/ Research Assistants	Lay Health Workers	Week 24 of study participation

Costing Data

	Data Collector	Timing
Fixed Costs	Graduate Research Assistant	Baseline
Variable Costs	Graduate Research Assistant	Week 12 then quarterly

Process Variables

Tool	Data collector	Timing
Activity Logs	Research Assistant/ LHW	Monthly during intervention phase
40-item Program Sustainability Assessment Tool	Investigators/ Research Assistants	Intervention and maintenance phases

Power and Sample size

Our sample size is based on the hypothesis that interventions will have a substantial impact on primary endpoints compared to the control phase (Figure 5).

Main effect: Based on a meta-analysis of 24 randomized controlled trials in LMIC[33], smoking abstinence at 6 months follow-up ($P_{control}$) for usual care averages 10% (range, 8% to 14%). Assuming withdrawal from trial follow-up or loss to follow-up to be 5%, our sample of 25 PLWH per cluster per period has power of 87% to detect at least 10% increase (main effect, Δ = ($P_{implementation}$ -$P_{control}$) in the primary endpoint at six months with the assumption that the event rate for those in control phase is 10% and two-sided alpha at intra-cluster correlation of 0.02.

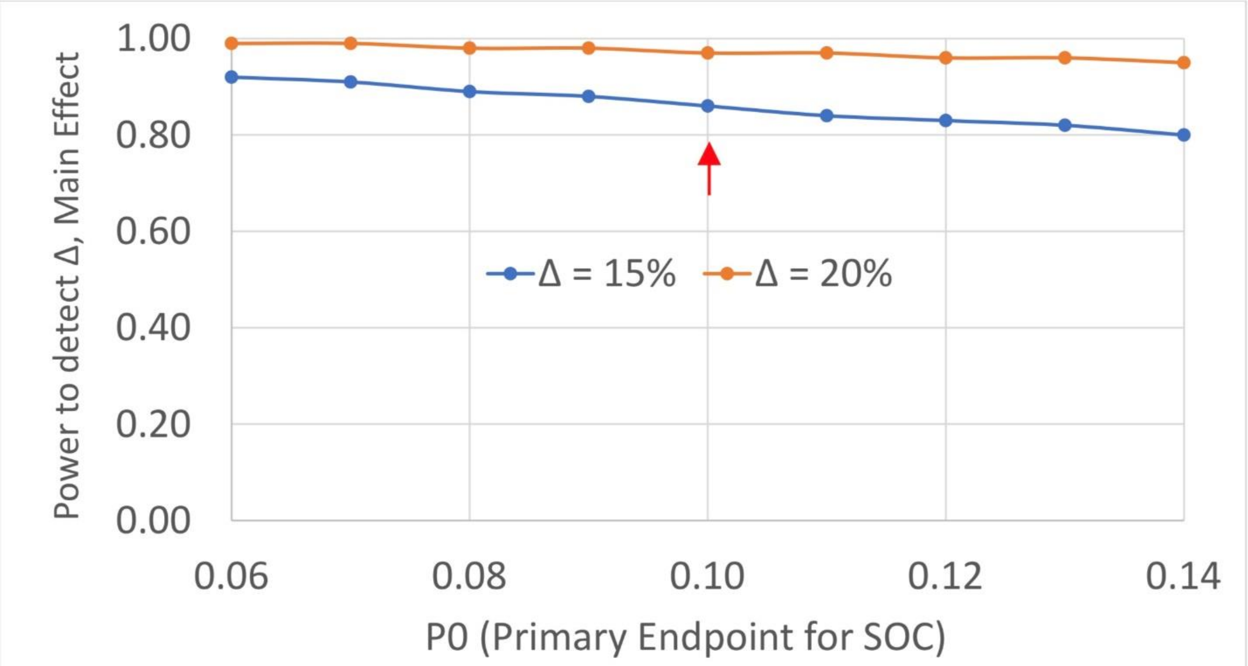

Fig 5. Power to detect primary endpoint, main effect versus standard of care (SOC)

Statistical analysis

Statistical Analysis for Study Objective 1.

We will analyze "Reach" as the percentage of PLWH who participated in the screening for tobacco use for each facility and compare between the control and implementation group using chi-square test. We will examine sociodemographic and clinical characteristics of smokers who did not participate. As analysis of primary outcome with intention-to-treat, the clinical effectiveness endpoint will be compared between the control group and implementation group at the individual level with a generalized linear mixed model with a binary distribution using the jack-knife method to estimate standard errors to account for grouping within clusters and by incorporating a log-link function to estimate the relative risk as a measure of effect[34,35]. We will include random effects to account for the clustering within facilities and periods and fixed effects for type of facilities. The model will be selected as the best model with unique covariance structure that produces the lowest Bayesian Information Criterion (BIC) value. The covariance structures that will be considered in the model are the first order of autocorrelation covariance structure, unstructured covariance structure, and Toeplitz covariance structure. We will use the Satterthwaite method to adjust for denominator degree of freedom for the test for fixed effects. The random coefficients will be modeled using G-side random effects, and we will obtain the subject-specific estimates by defining the appropriate variance-covariance structures.

Sex-stratified and age-stratified analyses and models will be performed as secondary analyses. Given high quality of data and experience in conducting studies in Botswana, we do not expect to have missing observations in the variables required for the primary analysis. To address the concern about contamination, we will conduct a sensitivity analysis excluding patients in the control group in the 90 days before crossing over to the implementation group. Because there will be fewer than 50 clusters, we will conduct a sensitivity analysis using a small sample correction with the Kenward-Roger method[36].

As analysis of secondary outcomes at 6 months, 30-day point prevalence abstinence will be compared between the implementation and the control groups in a similar model to the one used in the analysis of the primary outcome. Longitudinal differences in the Stages of Change Algorithm between the implementation and the control groups will be compared using random-mixed effect regression. Analysis will be conducted to assess factors associated with completion of varenicline regimen using generalized estimating equation model for binary repeated measures[37].

Statistical analysis for Study Objective 2

The CFIR will be used to guide qualitative interviews and activity log coding analysis. The CFIR-ERIC Matching Tool[38,39], which provides a menu of 39 constructs across five domains (Intervention Characteristics, Inner Setting, Outer Setting, Characteristics of Individuals, and Process), will be used to generate a list of potential strategies to address implementation barriers.

Qualitative data analysis. The CFIR will provide the structure for the initial codebook to guide qualitative data and activity log data using a descriptive content coding approach. The coding approach will be both deductive (codes derived from CFIR constructs) and inductive (codes derived from the data). During the coding and rating phases of analysis, the analysts will be blinded to the implementation effectiveness. Data will be independently coded by a revolving team of two analysts. Codes will be compared, and differences will be resolved by consensus discussion.

Quantification of qualitative data. We will quantify qualitative data and activity log data by having two analysts independently assign ratings for each code to reflect its positive or negative impact on implementation. Ratings will range from –2 to +2 with 0 to reflect a neutral or mixed influence of the code. For each facility, data will be consolidated, and two analysts will independently rate each code across all the interviews to obtain a facility rating for each time period. This process will be repeated for the two rounds of interviews and monthly for activity logs.

Quantitative measure of implementation success. Implementation effectiveness will be assessed by measuring the extent of integration of BSMART into the clinical setting within participating facilities. Therefore, we will focus on ***Adoption*** which will be analyzed monthly and for the 12 months of implementation as the number of clinic days per month LHW case managers delivered SBIRT divided by the number of operating clinic days per month. ***Implementation with fidelity*** will be analyzed monthly and for the 12 months of implementation period as the number of SBIRT provided with repeated intervention over time by LHW case managers to PLWH who smoked divided by the number of PLWH who are eligible for the program. Pearson's correlation will be used to assess the strength of correlation between qualitatively derived construct ratings from qualitative interviews and activity logs, and implementation effectiveness across facilities.

Statistical Analysis for Study Objective 3

RE-AIM measures will be re-assessed at 24 months after BSMART implementation to provide a standardized evaluation approach to foster understanding of whether the impact and implementation delivery are maintained and to shed light on where sustainability issues arise e.g. which clinic and population the implementation is not reaching. Trends in the outcomes of clinical effectiveness (collected and analyzed in Study Objective 1) and implementation effectiveness (collected and analyzed in Study Objective 2) will be compared between the implementation period and the maintenance period using chi-square test.

Statistical analysis for study Objective 4.

Incremental cost-effectiveness ratio and net monetary benefit. The incremental cost-effectiveness ratio (ICER) is calculated as the ratio of the difference in mean costs and the difference in mean 7-day point prevalence abstinence at 6 months. The ICER (Figure 6) quantifies the additional cost associated with a unit change in the 7-day point prevalence abstinence at 6 months, comparing between the implementation and control groups. Net benefit (NB) regression[40,41] provides an appropriate approach to quantify the ICER using the incremental net monetary benefit (INMB) and produce a confidence interval around the ICER.

$$ICER = \frac{Cost_{Implementation} - Cost_{Control}}{Effect_{Implementation} - Effect_{Control}}$$

Fig 6: ICER

In equation 1 of Figure 7 we define NB as the difference between the monetized total effect measure (the product of lambda, the willingness to pay per unit of effect, and the total effect units available, e) and the associated costs. The NB regression model is specified in equation 2 of Figure 7 as the difference between effects and costs, estimated via a parametric regression framework where errors are represented by the final term in equation 2, epsilon. Alternative formulations of equation 2 include a generalized linear regression model that incorporates a skewed distribution of the NB. We will identify the initial value of lambda following a targeted literature review and consultations with onsite study collaborators. In equation 2 of Figure 5, the NMB, represented by the β coefficient on the indicator variable for receipt of intervention (i.e., 'Implementation'), will be estimated using the NB regression model.

$NB_{ij} = (\lambda - e_{ij}) - c_{ij}$……………………………………………(1)

$NB_{ij} = \beta_0 + \beta 1 \times implementation_{ij} + \varepsilon_{ij}$…………. (2)

Fig 7: Net Benefit

Sensitivity Analysis: Uncertainty in the estimated incremental cost-effectiveness ratios (ICERs) will be represented using the cost-effectiveness acceptability curve[40,42]. Parameter uncertainty in the choice of the economically preferred intervention (between control and implementation periods) will be represented by the conditional net benefit (cNB) curve. The cNB curve plots the cNB (i.e., the net benefit value given a particular value of the parameter of interest) across centiles of the distribution of the parameter of interest. Sensitivity analyses will include probabilistic one-way sensitivity analyses (POSA)[43] of cost input parameters (e.g., drug costs). Uncertainty in cost inputs will be investigated via designed simulations that use alternative values of cost inputs as determined by draws from assigned distributions (e.g. gamma, lognormal), with distributional parameters estimated from data collected from ongoing studies in Botswana. The POSA varies a specific input parameter value across its full distribution while accounting for concurrent variation in all other parameter values using Monte Carlo simulation, The cNB curve developed from the planned POSA will allow decision makers to identify the impact of the value taken by a specific cost parameter on the value of the BSMAART intervention.

Study timeline

BSMART is a 5-year study:

Year 1: Protocol approvals will be obtained. A stakeholder meeting will be initiated. Standard operating procedures and manuals will be developed. Training of implementation leaders, lay health worker (LHW) case managers, nurse prescriber-dispensers (NPD), research assistants, and other facility healthcare workers will be conducted. Focus group discussions and Semi-Structured Interviews will be held with study participants, LHW case managers and NPDs.

Year 2: Screening and enrollment will be 12 months. In the trial, each participant will be followed for 24 weeks. Trial quality and compliance will be heavily monitored within the context of intervention implementation, data collection, and data quality.

Year 3: Qualitative studies on trial adoption and implementation delivery will be conducted. Participants' follow-up will be concluded.

Year 4: Analysis of trial primary endpoints and subgroup analysis will be conducted.

Year 5: Preliminary cost evaluation of the intervention will be conducted to focus on integration of the interventions into the HIV care system. Analysis for maintenance at the patient level i.e. durability of the intervention and at the facility level i.e. continuation with the interventions after the withdrawal of the interventions will be conducted. A final stakeholder meeting to focus on dissemination will be held.

	Year 1				Year 2				Year 3				Year 4				Year 5			
Timeline for Major Activities	Q1	Q2	Q3	Q4	Q1	Q2	Q3	Q4	Q1	Q2	Q3	Q4	Q1	Q2	Q3	Q4	Q1	Q2	Q3	Q4
Data Management system in place	X																			
Stakeholder Advisory Group & Practitioner Engagement Group Meetings		X	X	X	X	X	X	X	X	X	X	X	X	X	X	X	X	X	X	X
Protocols, surveys, approval		X																		
Adaptation of intervention through qualitative FGs with CMs and NPDs		X	X																	
U01 network meeting with NIH/NCI			X				X				X				X				X	
Training of LHW CMs and NPDs			X	X	X		X	X	X		X	X	X							
Recruitment and enrollment				X	X	X		X	X	X		X	X	X						
Follow-up to 24-weeks (post intervention)					X	X	X	X	X	X	X	X	X	X	X	X	X	X	X	X
Interviews on feasibility and acceptability		X	X	X	X	X	X	X	X	X	X	X	X	X	X	X	X	X	X	X
Analysis of trial outcomes								X	X			X	X	X	X		X	X	X	X
Analysis of qualitative implementation data														X	X	X	X	X		
Preliminary cost evaluation								X	X			X	X			X	X	X	X	
Analysis of durability and HIV data																	X	X	X	

Ethics and Protection of Human Subjects

Risks to Human Subjects

All UMB research meets the requirements for the conduct of research using funds from the U.S. Government. We maintain Federal Wide Assurances (FWA) and with the NIH Office for Human Research Protections (OHRP)- approved institutional review boards (IRBs) in Botswana and the U.S. This protocol, the informed consent documents, study procedures, study documents, and any subsequent modifications will be reviewed and approved by all relevant IRBs responsible for the oversight of the study – the UMB IRB and the Health Research Development Committee (HRDC) of Botswana. All staff who will have contact with participants will receive training on the protection of human subjects in research prior to conducting any study activities and bi-annually thereafter. Key staff will also complete Good Clinical Practices training every three years.

1. Human Subjects Involvement, Characteristics, and Design

The proposed research will involve human subjects as study participants. All procedures will be conducted according to the local standards of routine clinical care. Guided by the RE-AIM framework, our proposal will assess the Reach and Effectiveness of the BSMART interventions, its Adoption and Implementation, and how the BSMART interventions are Maintained over time and across outpatient HIV care facilities. In addition to the implementation trial, we will also conduct semi-structured interviews (SSIs) with study participants, and facility healthcare workers. SSIs conducted by the research team will be used to elicit perceptions, barriers, and stigma from participants at week 24. Focus group discussions (FGDs), which are ideal for identifying normative understandings following the generation of multiple perspectives, will be used with lay healthcare workers and nurse prescribers around the challenges of implementing the intervention and its integration with HIV care. Participants have the right to withdraw from the study procedures, semi-structured interviews, and focus group discussions at any time.

UMB's HIV treatment and care projects in Botswana:

UMB, the Prime Applicant for this study, and the Botswana University of Maryland Medicine Health Initiative (BUMMHI), a subrecipient for this study, currently collaborate on the Accelerating Botswana through the Last Mile to Epidemic Control (ABLE) project providing support for HIV care and treatment in 53 high-volume facilities across Botswana, including the 15 selected and 4 reserve sites proposed for this study, and will provide support through 2025, when the human subjects research at the facilities will be performed. The Botswana National AIDS and Health Promotion Agency (NAHPA) has confirmed the

investigators’ access to these facilities to recruit participants and conduct the proposed study (see Letters of Support).

Characteristics of the study population:

Participants will be drawn from the population of HIV-infected patients receiving care at 15 selected health facilities that are part of the ABLE project (see Facilities and Other Resources). We plan to screen approximately 6,900 HIV-infected patients for tobacco smoking and subsequently enroll 750 patients who are interested in quitting tobacco smoking into the trial, 375 of whom will participate in the pre-implementation phase and 375 of whom will participate in the implementation phase across 3 waves of implementation at 5 facilities each. Study participants will receive HIV care and treatment according to national standards. Participants will be eligible to participate in the study if they meet the following criteria:

Inclusion criteria:

(1) HIV-infected

(2) self-reported daily smoker

(3) age 18 years and older

(4) engaged in HIV care as defined by being on ART for at least 6 months

(5) willing/able to provide informed consent in English or Setswana

Exclusion criteria:

(1) pregnancy or nursing

Informed consent will be administered in the participant’s choice of English or Setswana language.

We will ensure ethical conduct of the study through respecting and protecting the interests of all potential and enrolled participants, building trust between researchers and participants, and conducting the study under the principles of good clinical and laboratory practices.

Participant sampling and recruitment: Recruitment activities will begin at the HIV care services through introduction of the study by the lay health worker (LHW)/Case Managers or clinical nurse dispenser/prescribers. Tobacco use screening will occur within the context of HIV outpatient care. The LHW Case Manager will identify individuals who initially screen positive for tobacco use. They will be

linked with a research assistant also stationed in the HIV outpatient clinic who will lead them through the informed consent process and then offer enrollment to those interested in participating in the study. Individuals who present at the study clinic for screening and enrollment will complete the informed consent process on a first come, first serve basis prior to the completion of any study specific activities.

Individuals participating in interviews or focus group discussion for the qualitative analysis will also complete a consent process prior to completion of any study activities. We will randomly select enrolled participants to complete interviews and will recruit individuals who failed to abstain until we reach our target sample size.

Retention: We have maintained high retention rates under the ABLE project, as evidenced by high viral load suppression rate of 99%. We will leverage the current retention approaches under the ABLE project and include additional strategies. Retention strategies include information leaflets about the importance of retention, appointment cards, motivational text messages, and phone calls (and home visits) to remind participants of appointments. All participants who miss study visits will be contacted.

2. Sources of Materials

Clinical care material will be obtained through extraction of data, and records, including longitudinal clinical and laboratory measurements, and will be available as part of informed consent for the study from the Integrated Patient Management System (IPMS) and the Patient Information Management System (PIMS); the two main electronic medical record (EMR) systems in use in Botswana. Clinical information with individual identifiers will not be released without the written permission of the participants which will be obtained as part of informed consent.

Research material that will be obtained from living individuals will include breath and data. Data collected will include information regarding demographics and socioeconomic characteristics, medical history, tobacco use, and substance use.

Additional materials include survey questionnaires and assessments administered at different timepoints, as well as focus group discussions and semi-structured interviews. Qualitative data will be digitally recorded, transcribed verbatim and translated into English. When the recordings are not being transcribed, they will be maintained in a locked storage cabinet.

Careful data management procedures will be implemented to ensure unique identification and proper ID linkage for all patient visits, data, clinical, demographic data, biologic specimens, and qualitative data. All

data will be secured in a locked file cabinet and password-protected database accessible only by key study personnel. The sites will maintain appropriate medical and research records for this study in compliance with International Council for Harmonization (ICH) E6, section 4.9 on "Records and Reports," and regulatory and institutional requirements for the protection of confidentiality of participants.

Any publication about this research study will omit names and any other personally identifiable information. However, individually identifiable private information may be reviewed by the following groups to monitor the conduct of the study and participant safety: The Ministry of Health and Wellness, the HRDC, the National Institutes of Health, the Office of Human Research Protections, and the UMB IRB.

3. Potential Risks to Subjects

We expect participant risk to be greater than minimal risk though this is offset by the existing and well-trained expertise of the research staff and clinical staff at the facilities engaged in this study. Potential risks to the subjects are the following:

i. Risk of breach of Confidentiality

There is the potential risk of breach of confidentiality regarding healthcare data, HIV status, and/or smoking status. At each of the steps in the study, we will protect participant privacy and confidentiality to obviate these risks e.g. consenting in private setting, not including names on case report or survey forms, etc.)

ii. Potential risks/side effects of quitting smoking

These include headache and nausea, tingling in hands and feet, coughing and sore throat, increased appetite and associated weight gain, intense cravings for nicotine, irritability, anxiety, and depression.

At each study facility, we will train the nurse prescriber/dispensers directly involved in managing the patients on these issues and identify referral systems to civil society support groups or other healthcare services in in order to ensure that these issues can be adequately addressed. In addition, information on these side effects will be discussed with the patients.

iii. Potential risks/side effects of varenicline:

These include nausea (up to 30%). In addition to nausea, other side effects occurring >5% of clinical trials and twice the rate in placebo were abnormal dreams, constipation, flatulence, and vomiting. Also in rare cases, changes in behavior, hostility, agitation, depressed mood, suicidal thoughts/actions, rash, and sensitivity reactions have occurred.

We will closely monitor for depressive and other neuropsychiatric symptoms and have a careful plan in place for any exacerbation of symptoms or suicidality. All participants will be thoroughly informed of the various side-effects of varenicline, which they might experience and will be appropriately cautioned concerning their activities in the hours after administration. Since participation is voluntary, participants can withdraw at any time if they find the behavioral procedures or drug effects undesirable. Each time a participant receives their medication, the prescribing clinicians will assess for adverse side effects using a standardized questionnaire. If a participant reports depressed mood or suicidal ideation, the participant will receive an immediate evaluation by a qualified clinician to ensure participant safety and proper provision of treatment. Treatment with varenicline will be stopped for any participant experiencing these types of adverse events. Further participation in the study will be decided upon with input from the treating clinician, the research team and the participant.

We are aware that in September 2021 Pfizer voluntarily recalled varenicline (marketed under the brand name Chantix) due to concern that the medication may contain levels of N-nitroso-varenicline above the FDA limit (37ng per day). It is important to note that other makers of varenicline (i.e., Apotex) did not have this problem with their manufacturing of varenicline and remain FDA approved. https://www.fda.gov/drugs/drug-safety-and-availability/fda-updates-and-press-announcements-nitrosaminevarenicline-chantix

Adequacy of Protection Against Risks

1. Informed Consent

Individuals will be asked to provide informed consent for the trial as well as for the focus group discussions and semi-structured interviews. Research assistants will provide a printed version of the signed informed consent to each participant as well as any study information the participant requests to have printed. The written consent will be provided in the participant's choice of English or Setswana and administered by trained research assistants. Research assistants will review the document with each participant in a private environment, respond to questions, and emphasize participant rights regarding research

participation. We will use assessment tests in the consent process to ensure that the participants comprehend the study and are able to provide informed consent. The ability to provide informed consent is part of the inclusion criteria for this study. Participants will have the right to withdraw participation at any time, without interruption to their health and HIV care and treatment services rendered at the health facility.

2. Protections Against Risk

Risk to the participants will be minimized by thorough training and supervision of all staff. Care has been taken to protect the privacy as well as confidentiality of participants, and to safeguard all data that is linked from electronic medical records or newly collected as part of the study. Additionally, the study will take place at current high-volume facilities already providing care to the HIV patients to be recruited, and with appropriate safeguards in place to protect patient privacy and confidentiality and to keep all medical records secure. All study data, reports and administrative forms will be identified by a coded number only to maintain participant confidentiality. All databases will be secured with password-protected access system, and computer entries will be identified by coded number only. Forms, appointment books, audio-recordings, and any other data forms that link participant ID numbers to other identifying information will be stored in a separate, locked cabinet. All data analysis will be done on datasets which have only the study number as a unique identifier. Clinical information with individual identifiers will not be released without the written permission of the participant. We expect these procedures to adequately protect participant confidentiality. However, it is possible that a participant's study participation or HIV status or smoking status could become known to people in the community and may result in stigma or discrimination. Should that occur, study staff will work with the participant and his/her family as appropriate to resolve the situation in whatever manner is preferred.

Participants referred to treatment with varenicline will be taught the correct use of varenicline by the nurse prescriber-dispenser (NPD) at the site where the participant receives care. Participants will initiate medication treatment with varenicline for smoking cessation with a quit date scheduled for day 8 following first study dose of the medication. Participants will meet with the NPD at baseline who will provide medical clearance and sign off on prescription orders. All medication will be provided to participants by the study team. Participants will receive a weekly supply of medication for the first four weeks to ensure proper dosing and monitoring for adverse events. For the subsequent 8 weeks, participants will return every 4 weeks to receive the next month's supply of medication. Dosage adjustments will be permitted in an effort to control adverse effects throughout the trial. This will allow

us to balance internal validity with good clinical practice. Varenicline will be prescribed in accordance with package labeling. Adverse events monitoring will occur by the study clinician each time a participant picks up their medication.

A centralized training will be conducted for all licensed clinicians eligible to prescribe varenicline, the nurse prescribers/dispensers, which will be scheduled and led by trained Implementation leaders under the guidance of Dr. Himelhoch. The training will provide the background and orientation to treatment with varenicline. A consultation group will be established to address questions clinicians may have when prescribing varenicline. These trainings will be repeated for the sites in every step of the study (Step 1 , step 2 and step 3 - see Table 1: Time periods for Stepped Wedge Implementation).

Per IRB guidelines, all research staff will be trained and certified on Human Subjects Research through the Collaborative IRB Training Initiative (CITI). Additionally, per UMB guidelines any staff conducting human research must complete HIPAA 125 (formally 101 and 120) and the HIPAA 201 which is provided by the University of Maryland Baltimore and will be made available to all local staff. Training above will be completed prior to any involvement in the implementation of the research protocol.

Potential Benefits of the Proposed Research to Research Participants and Others

There is a direct benefit to research participants, who will all receive smoking cessation handouts and other education materials as part of SBIRT and the 5”A”s. For research participants in the implementation phase, exposure to SBIRT and using varenicline as a smoking cessation aid can impact their future smoking abstinence, future quit attempt, and overall health management. There is substantial health benefit for those individuals who reduce smoking frequency or achieve smoking abstinence as a result of the intervention(s) provided, including those who do not achieve smoking abstinence during the study but acquire the tools to be successful in a future quit attempt. For those who have sustained smoking abstinence, the health benefit will continue to increase even after the study has ended, and lead to lung cancer control.

Also, since PLWH who smoke are more susceptible to HIV-related opportunistic infections and noncommunicable diseases, a reduction or elimination of smoking will likely improve their overall health and well-being. Smoking can also negatively impact ART effectiveness, so reduced smoking may improve the response of research participants to ART, and as a result, their overall HIV management and health.

For the health workers at the facilities engaged in screening and providing the intervention, LHW Case Managers and clinical nurse dispenser/prescribers respectively, the proposed study will provide them with additional screening skills and tools, which can be applied to benefit other patients who smoke.

Importance of Knowledge to be Gained

As a result of the proposed research, we will gain knowledge about the effectiveness of Screening, Brief Intervention and Referral to Treatment (SBIRT) through the 5”A”s in conjunction with varenicline for achieving smoking abstinence and lung cancer control in PLWH in low- and middle-income countries (LMICs). Through focus groups and semi-structured interviews, the study will also provide information about challenges of implementing the intervention and integration with HIV care delivery. Additionally, this study will generate information about the cost effectiveness and incremental costs of implementing SBIRT with varenicline, for scaleup across Botswana and to similar LMIC settings, and potentially have implications for using similar task sharing models for delivery of smoking cessation and other cancer control interventions globally.

Data and safety monitoring plan

For all enrolled participants, study staff will collect information on events related to study involvement at each follow up visit including adverse events, perceived social harm, and events related to side effects related to quitting smoking. In addition to these events which we will monitor closely, we can expect that this population of HIV-infected men and women will experience adverse events unrelated to study procedures including new diagnoses of opportunistic infections, side effects from ART medications, hospitalization, and possibly death. Participant records will be carefully monitored internally by the study coordinator, project director, and investigators to ensure that no adverse events or social harms have been missed. We expect events to be minimal, but all will be documented, assessed for seriousness/severity and relatedness, carefully monitored, and managed by the onsite investigators. Our trial will adhere to the NIH adverse event reporting guidelines. All study-related adverse events will be reported to the MPI and to all regulatory bodies following the requirements of each committee.

Confidentiality / Data Management and Security

Study participants will be assigned a coded identification number at the time of study enrollment. In order to maintain patient confidentiality, all laboratory specimens, study case report forms, and reports will be identified using that coded number. Only research staff will have access to the unique coded number. Key study personnel will store research data (including medical and laboratory records) in locked cabinets, and all e-files will be password protected. Data will be stored in password-protected files. Access to all data will be ID- and password protected, including data warehouse software and computers managing and analyzing survey data. No data will be released with any information that may directly or indirectly identify participants, their clinical information, or laboratory results to outside agencies.

Protocol Compliance

Serious Adverse Event (SAE) Reporting: Safety monitoring for this study will focus on reportable new information of events that include unanticipated problems involving risks to participants, including unanticipated problems that meet the definition of a serious adverse event.

Unanticipated Problems: The Office for Human Research Protections (OHRP) considers unanticipated problems involving risks to subjects or others to include, in general, any incident, experience, or outcome that meets all of the following criteria:

- Unexpected in terms of nature, severity, or frequency given (a) the research procedures that are described in the protocol-related documents, such as the IRB-approved research protocol and informed consent document; and (b) the characteristics of the subject population being studied;
- Related or possibly related to participation in the research (possibly related means there is a reasonable possibility that the incident, experience, or outcome may have been caused by the procedures involved in the research); and
- Suggests that the research places subjects or others at a greater risk of harm (including physical, psychological, economic, or social harm) than was previously known or recognized.

Serious Adverse Events: A serious adverse event (SAE) is one that meets one or more of the following criteria:

- Results in death
- Is life-threatening (places the subject at immediate risk of death from the event as it occurred)
- Results in inpatient hospitalization or prolongation of existing hospitalization
- Results in a persistent or significant disability or incapacity
- Results in a congenital anomaly or birth defect

An important medical event that may not result in death, be life threatening, or require hospitalization may be considered an SAE when, based upon appropriate medical judgment, the event may jeopardize the subject and may require medical or surgical intervention to prevent one of the outcomes listed in this definition.

Reporting Procedures

Incidents or events that meet the OHRP criteria for unanticipated problems require the creation and completion of an unanticipated problem report form. OHRP recommends that investigators include the following information when reporting an adverse event, or any other incident, experience, or outcome as an unanticipated problem to the IRB:

- appropriate identifying information for the research protocol, such as the title, investigator's name, and the IRB project number, a detailed description of the adverse event, incident, experience, or outcome;

- an explanation of the basis for determining that the adverse event, incident, experience, or outcome represents an unanticipated problem,
- a description of any changes to the protocol or other corrective actions that have been taken or are proposed in response to the unanticipated problem. To satisfy the requirement for prompt reporting, unanticipated problems will be reported using the following timeline. Unanticipated problems that are serious adverse events will be reported to the UMB IRB and the Health Research Development Committee (HRDC) in Botswana within 1 week of the investigator becoming aware of the event. Any other unanticipated problem will be reported to the UMB and HRDC within 2 weeks of the investigator becoming aware of the problem. All unanticipated problems will be reported to appropriate institutional officials (as required by an institution's written reporting procedures), the supporting agency head (or designee), and OHRP within one month of the IRB's receipt of the report of the problem from the investigator.

Data Safety and Monitoring Board (DSMB)

An independent DSMB will be established according to ensure compliance with NIH regulations on clinical trials. The membership of the committee will include expertise in behavioral medicine, substance use, biostatistics, and clinical randomized trials, HIV care, and clinical research. The responsibilities of the DSMB will be to monitor data from the trial and to advise the sponsor and study leadership on any recommended changes to the conduct of the study, including early termination for futility or for unexpectedly high benefit on the primary endpoint if appropriate. A formal interim analysis is not anticipated, as it is important to measure the effect of the intervention over the full proposed period of time. However, data from the study communities on operational performance including uptake and adverse events will be reported to the DSMB and reviewed on a monthly basis via electronic data capture, facilitating timely analysis. We will monitor adverse outcomes and other items above during the intervention on a monthly basis. We will convene the DSMB every six months during the intervention period in order to review progress and safety issues. Reports to the DSMB will include:

(1) assessments of recruitment, accrual, retention, safety, and data quality; and

(2) consideration of new data that may become available, including scientific or therapeutic developments that may have an impact on participant safety or the ethics of the trial. We will pause enrollment or stop the study altogether for reasons of patient safety.

Data Collection and Storage

All study data will be collected electronically by trained research assistants during study activities. Data entry to the tablet-based electronic database (REDCap) will be performed by trained study staff.

Research Electronic Data Capture- REDCap: REDCap is an application for building and managing online databases primarily created for electronic data collection, storage and transmission. UMB has developed and currently supports secure, local installation of REDCap for ongoing studies in Botswana. REDCap provides a web-based interface for collecting data with data validation and includes the ability for automated export to statistical packages. The software also includes data logging for HIPAA compliance and the ability for administrators to define access rights on a per-user basis.

Data Safety and Security

Access credentials: Only authorized users with usernames and passwords will be given access to the REDCap database by the administrators. Users will be made aware that providing user IDs or sharing passwords with unauthorized individuals is a BREACH OF CONFIDENTIALITY and is grounds for appropriate disciplinary action. Access will be granted to only IRB approved study team members who are trained in protecting personal health information (PHI), have completed the CITI certification and who will access REDCap on secured networks and devices.

Ensuring Data Quality

The approach to data quality is based on the following key points:

Components of data quality	Approach
1. Data Completeness	Minimum missing values
2. Data Accuracy	Matching values in the database with original observation
3. Data Precision	Defining units and measurability
4. Timely collection	Minimal time loss between observation and recording
5. Data Verification	Independent assessment or monitoring
6. Data Tracing	Logging actions taken while handling the data

Above points will be ensured as follows:

a) Data Validation and Queries

The data manager will assess data quality using REDCap's Data Quality module. Any identified issues will either be corrected or documented in the study data handling manual. Electronic data collection forms will be programmed with online validation checks. These checks will:

• Alert data entry users to missing data

• Check that numeric variables and dates are within reasonable ranges

• Check for consistency within the data

b) Data Quality Audit

At intervals throughout the study a subset of entered data will be compared with the original data collection forms. The number of compared forms and error counts will be recorded in order to measure the percentage data entry error rate.

These audits will be conducted using random samples as follows:

- 10 of the first 100 subjects
- 20 of the first 200 subjects
- 75 of the total 750 subjects

The error rate for study data will be maintained below 0.1%. If error rates remain high for three consecutive samples the team will need to consider alternative remedies in order to bring the error rate within acceptable limits.

1. Data Extraction and Transfer

Data can be exported by authorized users only, using REDCap's data export module. De-identified study data will be exported into an Excel sheet and transmitted via Dropbox to co-investigators on a weekly basis.

2. Archiving and Destruction

After study completion, all study materials will be stored in a locked shelf, inside a locked room at the PI's office at UMB. Subsequently, the study database on the REDCap system will be archived at UMB.

Dissemination Plan

We have plans to disseminate findings from the proposed study at various levels, in compliance with NIH policy:

1. ClinicalTrials.gov: We will register BSMART within the first 6 months of the funding award.
    - All submitted information will be updated semi-annually or when modifications are required.
    - Any apparent errors, deficiencies, and/or inconsistencies identified by the NIH as part of the quality control review process will be addressed by Dr. Charurat, MPI (Contact).
    - Corrections to submitted information will be made within 15 days for registration information and 25 days for results information.
    - Trial results will be submitted no later than one year after the primary completion date.
    - Informed consent documents for the trial enrollment will include specific statement relating to posting of the clinical trial and results at ClinicalTrials.gov.
    - The University of Maryland Baltimore has an internal policy in place to ensure that clinical trial registration and result reporting occur in compliance with the NIH policy requirements.

2. Local Dissemination of study findings: We have engaged the community in the study design. In addition, our experiences with previous studies in Botswana suggest that sharing aggregate data from the trial with stakeholders, including NAHPA, MOHW, the Anti-tobacco Network and the community members, builds significant trust. We will provide each relevant stakeholder with aggregate information derived from study data in meaningful and appropriate ways to improve health access, outcomes, and equity. The Stakeholder Advisory Group and Practitioner Engagement Group will provide ongoing input into dissemination efforts.

3. National Dissemination: To ensure successful integration of our proposed research into broader practice and policy, we will continue to leverage our strong partnerships with the MOHW, NAHPA, UB, civil societies, and PEPFAR/CDC in Botswana. Training of LHW Case Managers is an essential part of a cost-effective, evidence-based strategy on smoking cessation and treatment of tobacco dependence because of their interaction with smokers and tobacco consumers as care providers and their role as health communicators. We will also leverage our relationship with the Anti-

tobacco Network as a technical partner of the study (Dr. Mbongwe, BSMART MPI) to re-invigorate the national smoking cessation campaign that is underpinned by interventions that have been developed and tested locally.

4. Regional Dissemination: We will also focus on sharing findings at regional meetings in sub-Saharan Africa, such as the African Organization for Research and Training in Cancer (AORTIC) annual conference. We will submit abstracts for presentation at this conference to share our study findings.
5. International Dissemination: We will submit abstracts to international conferences such as the Consortium of Universities for Global Health (CUGH) and the International AIDS Society conference.
6. Publications: We expect to have a series of peer-reviewed articles generated from our study throughout the project period and have allotted funds in our budget to make these publications open access for ease of reading, especially for those in other low- and middle-income countries (LMICs).

References

1. Ashare RL, Thompson M, Serrano K, et al. Placebo-controlled randomized clinical trial testing the efficacy and safety of varenicline for smokers with HIV. *Drug Alcohol Depend*. 2019;200(December 2018):26-33. doi:10.1016/j.drugalcdep.2019.03.011

2. Ebbert J. Varenicline for smoking cessation: efficacy, safety, and treatment recommendations. *Patient Prefer Adherence*. 2010;4:355. doi:10.2147/ppa.s10620

3. Deeken JF, Tjen-A-Looi A, Rudek MA, et al. The rising challenge of non-AIDS-defining cancers in HIV-infected patients. *Clin Infect Dis*. 2012;55(9):1228-1235. doi:10.1093/cid/cis613

4. Hessol NA, Martínez-Maza O, Levine AM, et al. Lung cancer incidence and survival among HIV-infected and uninfected women and men. *Aids*. 2015;29(10):1183-1193. doi:10.1097/QAD.0000000000000690

5. Engels EA, Pfeiffer RM, Goedert JJ, et al. Trends in cancer risk among people with AIDS in the United States 1980-2002. *Aids*. 2006;20(12):1645-1654. doi:10.1097/01.aids.0000238411.75324.59

6. Shiels MS, Pfeiffer RM, Gail MH, et al. Cancer burden in the HIV-infected population in the United States. *J Natl Cancer Inst*. 2011;103(9):753-762. doi:10.1093/jnci/djr076

7. Parka LS, Hernandez-Ramirez RU, Silverberg MJ, Crothers K, Dubrow R. Prevalence of non-HIV cancer risk factors in persons living with HIV/AIDS: A meta-analysis. *Aids*. 2016;30(2):273-291. doi:10.1097/QAD.0000000000000922

8. Engels EA, Brock M V., Chen J, Hooker CM, Gillison M, Moore RD. Elevated incidence of lung cancer among HIV-infected individuals. *J Clin Oncol*. 2006;24(9):1383-1388. doi:10.1200/JCO.2005.03.4413

9. Chaturvedi AK, Pfeiffer RM, Chang L, Goedert JJ, Biggar RJ, Engels EA. Elevated risk of lung cancer among people with AIDS. *Aids*. 2007;21(2):207-213. doi:10.1097/QAD.0b013e3280118fca

10. Clifford GM, Lise M, Franceschi S, et al. Lung cancer in the Swiss HIV Cohort Study: Role of smoking, immunodeficiency and pulmonary infection. *Br J Cancer*. 2012;106(3):447-452. doi:10.1038/bjc.2011.558

11. Sigel K, Wisnivesky J, Gordon K, et al. HIV as an independent risk factor for incident lung cancer. *Aids*. 2012;26(8):1017-1025. doi:10.1097/QAD.0b013e328352d1ad

12. Reddy KP, Kong CY, Hyle EP, et al. Lung cancer mortality associated with smoking and smoking cessation among people living with HIV in the United States. *JAMA Intern Med*. 2017;177(11):1613-1621. doi:10.1001/jamainternmed.2017.4349

13. Hoffman AC, Starks VL, Gritz ER. The impact of cigarette smoking on HIV/AIDS: Urgent need for research and cessation treatment. *AIDS Educ Prev*. 2009;21(SUPPL. 3):1-2. doi:10.1521/aeap.2009.21.3-supp.1

14. Nahvi S, Cooperman NA. Review: The need for smoking cessation among HIV-positive smokers. *AIDS Educ Prev*. 2009;21(SUPPL. 3):14-27. doi:10.1521/aeap.2009.21.3-supp.14

15. Hartmann-Boyce J, Stead LF, Cahill K, Lancaster T. Efficacy of interventions to combat tobacco addiction: Cochrane update of 2013 reviews. *Addiction*. 2014;109(9):1414-1425.

doi:10.1111/add.12633

16. Mdege ND, Shah S, Ayo-Yusuf OA, Hakim J, Siddiqi K. Tobacco use among people living with HIV: analysis of data from Demographic and Health Surveys from 28 low-income and middle-income countries. *Lancet Glob Heal*. 2017;5(6):e578-e592. doi:10.1016/S2214-109X(17)30170-5

17. Méndez D, Alshanqeety O, Warner KE. The potential impact of smoking control policies on future global smoking trends. *Tob Control*. 2013;22(1):46-51. doi:10.1136/tobaccocontrol-2011-050147

18. Gentilello LM, Rivara FP, Donovan DM, et al. Alcohol interventions in a trauma center as a means of reducing the risk of injury recurrence. *Ann Surg*. 1999;230(4):473-483. doi:10.1097/00000658-199910000-00003

19. Solberg LI, Maciosek M V., Edwards NM. Primary Care Intervention to Reduce Alcohol Misuse. Ranking Its Health Impact and Cost Effectiveness. *Am J Prev Med*. 2008;34(2). doi:10.1016/j.amepre.2007.09.035

20. Bernstein SL, Boudreaux ED. Emergency Department-Based Tobacco Interventions Improve Patient Satisfaction. *J Emerg Med*. 2010;38(4):e35-e40. doi:10.1016/j.jemermed.2008.03.034

21. Cunningham RM, Bernstein SL, Walton M, et al. Alcohol, tobacco, and other drugs: Future directions for screening and intervention in the emergency department. *Acad Emerg Med*. 2009;16(11):1078-1088. doi:10.1111/j.1553-2712.2009.00552.x

22. Krist AH, Davidson KW, Mangione CM, et al. Interventions for Tobacco Smoking Cessation in Adults, Including Pregnant Persons: US Preventive Services Task Force Recommendation Statement. *Jama*. 2021;325(3):265-279. doi:10.1001/jama.2020.25019

23. Barua RS, Rigotti NA, Benowitz NL, et al. 2018 ACC Expert Consensus Decision Pathway on Tobacco Cessation Treatment: A Report of the American College of Cardiology Task Force on Clinical Expert Consensus Documents. *J Am Coll Cardiol*. 2018;72(25):3332-3365. doi:10.1016/j.jacc.2018.10.027

24. Anthenelli RM, Benowitz NL, West R, et al. Neuropsychiatric safety and efficacy of varenicline, bupropion, and nicotine patch in smokers with and without psychiatric disorders (EAGLES): A double-blind, randomised, placebo-controlled clinical trial. *Lancet*. 2016;387(10037):2507-2520. doi:10.1016/S0140-6736(16)30272-0

25. Lloyd-Richardson EE, Stanton CA, Papandonatos GD, et al. Motivation and patch treatment for HIV+ smokers: A randomized controlled trial. *Addiction*. 2009;104(11):1891-1900. doi:10.1111/j.1360-0443.2009.02623.x

26. Stanton CA, Papandonatos GD, Shuter J, et al. Outcomes of a tailored intervention for cigarette smoking cessation among latinos living with HIV/AIDS. *Nicotine Tob Res*. 2015;17(8):975-982. doi:10.1093/ntr/ntv014

27. Society IB. Median Regression with Censored Cost Data Author (s): Heejung Bang and Anastasios A . Tsiatis Published by : International Biometric Society Stable URL : http://www.jstor.org/stable/3068588 REFERENCES Linked references are available on JSTOR for this ar. 2016;58(3):643-649.

28. Lin DY. Linear regression analysis of censored medical costs. *Biostatistics*. 2000;1(1):35-47. doi:10.1093/biostatistics/1.1.35

29. DiClemente CC, Prochaska JO, Fairhurst SK, Velicer WF, Velasquez MM, Rossi JS. The Process of Smoking Cessation: An Analysis of Precontemplation, Contemplation, and Preparation Stages of Change. *J Consult Clin Psychol*. 1991;59(2):295-304. doi:10.1037/0022-006X.59.2.295

30. DiClemente CC, Schlundt D, Gemmell L. Readiness and Stages of Change in Addiction Treatment. *Am J Addict*. 2004;13(2):103-119. doi:10.1080/10550490490435777

31. Catz SL, Jack LM, McClure JB, et al. Adherence to varenicline in the COMPASS smoking cessation intervention trial. *Nicotine Tob Res*. 2011;13(5):361-368. doi:10.1093/ntr/ntr003

32. Kohrt BA, Jordans MJD, Rai S, et al. Therapist competence in global mental health: Development of the ENhancing Assessment of Common Therapeutic factors (ENACT) rating scale. *Behav Res Ther*. 2015;69:11-21. doi:10.1016/j.brat.2015.03.009

33. Akanbi MO, Carroll AJ, Achenbach C, et al. The efficacy of smoking cessation interventions in low- and middle-income countries: a systematic review and meta-analysis. *Addiction*. 2019;114(4):620-635. doi:10.1111/add.14518

34. Hussey MA, Hughes JP. Design and analysis of stepped wedge cluster randomized trials. *Contemp Clin Trials*. 2007;28(2):182-191. doi:10.1016/j.cct.2006.05.007

35. Hemming K, Haines TP, Chilton PJ, Girling AJ, Lilford RJ. The stepped wedge cluster randomised trial: Rationale, design, analysis, and reporting. *BMJ*. 2015;350. doi:10.1136/bmj.h391

36. Kenward MG, Roger JH. Small Sample Inference for Fixed Effects from Restricted Maximum Likelihood Author (s): Michael G . Kenward and James H . Roger Published by : International Biometric Society Stable URL : https://www.jstor.org/stable/2533558 REFERENCES Linked references. *Biometrics*. 1997;53(3):983-997.

37. Lipsitz SR, Kim K, Zhao L. Analysis of repeated categorical data using generalized estimating equations. *Stat Med*. 1994;13(11):1149-1163. doi:10.1002/sim.4780131106

38. Waltz TJ, Powell BJ, Matthieu MM, et al. Use of concept mapping to characterize relationships among implementation strategies and assess their feasibility and importance: Results from the Expert Recommendations for Implementing Change (ERIC) study. *Implement Sci*. 2015;10(1):1-8. doi:10.1186/s13012-015-0295-0

39. Powell BJ, Waltz TJ, Chinman MJ, et al. A refined compilation of implementation strategies: Results from the Expert Recommendations for Implementing Change (ERIC) project. *Implement Sci*. 2015;10(1):1-15. doi:10.1186/s13012-015-0209-1

40. Hoch JS, Dewa CS. Lessons from Trial-Based Cost-Effectiveness Analyses of Mental Health Interventions. *Pharmacoeconomics*. 2007;25(10):807-816. doi:10.2165/00019053-200725100-00001

41. Willan AR, Briggs AH, Hoch JS. Regression methods for covariate adjustment and subgroup analysis for non-censored cost-effectiveness data. *Health Econ*. 2004;13(5):461-475. doi:10.1002/hec.843

42. Jain R, Onukwugha E. Sensitivity Analysis in cost-effectiveness studies: from guidelines to practice. *Pharmacoeconomics*. 2011;29(4):297-314.

43. McCabe C, Tramonti G, Sutton A, Hall P, Paulden M. Probabilistic One-Way Sensitivity Analy...pdf.

Pharmacoeconomics. 2021;39(1):19-24.

44. Calhoun A, Mainor A, Moreland-Russell S, Maier RC, Brossart L, Luke DA. Using the program sustainability assessment tool to assess and plan for sustainability. *Prev Chronic Dis*. 2014;11(2014):1-7. doi:10.5888/pcd11.130185

Appendix A: Informed Consent Forms

Appendix A1: Research Consent Form – Control Phase

Protocol Title: Botswana Smoking Abstinence Reinforcement Trial (BSMART)

Study No.: *HP-00102995*

Principal Investigators:

Dr. Manhattan Charurat	University of Maryland, Baltimore
Dr. Bontle Mbongwe	University of Botswana, Botswana
Dr. Seth Himelhoch	University of Kentucky, Kentucky

Sponsor: *National Cancer Institute (NCI)*

CONCISE SUMMARY:

We are inviting you to take part in this research study. Participation in this research study is voluntary. You can choose to participate or not in this study. You can change your mind about participating at any time. Whatever choice you make, you will still receive the medical care you are receiving now, and you will not have to give up any of your rights.

This research study is trying to test one method to help people with HIV who smoke to stop smoking. If you choose to participate, you will:

- Receive a brochure telling you how to stop smoking.
- Need to answer some questions and
- Do a test where you blow into a machine.

Your visits at the clinic could take about 2 hours and you will be asked to come for four visits over a period of six months.

The biggest risk you may have if you participate in this interview is that people may get to know that you are living with HIV and that you smoke.

It will not cost you any money to participate in this research study. You will receive 80 Pula (6$ USD) for transportation costs for each visit and there are four planned study visits.

The things I am about to tell you will give you more information about this study to help you decide if you would like to participate. Please listen carefully and ask the study staff to explain anything you do not understand. You will have a chance to ask questions before you make a decision to participate.

PURPOSE OF STUDY

The purpose of this study is to identify patients with HIV who smoke cigarettes and want to stop smoking and test a new way to help them stop smoking. We plan to include 750 people who have HIV and smoke cigarettes. We are working at 15 clinics.

PROCEDURES

After you agree to participate and fill out a consent form, you will be asked several questions to see if you are eligible to be in this study. We will be asking you questions about your smoking habits and health history. During the screening appointment, you will be asked to provide your medical history. We will also have you blow into a carbon monoxide breath machine to measure the amount of carbon monoxide in your lungs from smoking. This will inform us of your smoking activity.

If you pass all the screening procedures, you will be asked to complete a baseline interview. In this interview, we will ask you several questions. The topics will include: your smoking history, your mental health history, and questions about your daily routine.

Standard of care:

Standard of care will be administered by a trained lay health worker during the control phase of the study. Participants will be given a quit smoking brochure and a one session brief advice to quit smoking. This one-time session will last approximately 2 minutes.

Visit schedule:

You will be in the study for a total of 24 weeks. You will be interviewed when you agree to join the study and then after four weeks and at 12, and 24 weeks.

WHAT ARE MY RESPONSIBILITIES IF I TAKE PART IN THIS RESEARCH?

You will be expected to come to the clinic for your appointments and answer questions on your smoking habits. During three visits, you will blow into a breath machine.

POTENTIAL RISKS/DISCOMFORTS:

i. Risks associated with assessment interviews:

The risk from research interviews is minimal. During assessments you may be uncomfortable discussing your smoking habits or mental health history and treatments. You may become frustrated and tense if you encounter any difficulties while completing these measures. All interviewers are trained to recognize signs of distress or anxiety. You will be reminded that you can refuse to answer any question that makes you uncomfortable and may take breaks whenever you need.

ii. Risk of breach of confidentiality

There is a slight risk of breach of confidentiality (to minimize this we will use a unique study ID number for your information rather than your name).

iii. Risks associated with smoking cessation

(Nicotine withdrawal symptoms)

There are additional risks in this study that occur when you stop smoking or are trying to stop smoking. One of these is the stress of and symptoms associated with nicotine withdrawal. Some people feel depressed, are unable to sleep, get angry easily, are anxious, have difficulty concentrating, are restless, or may have decreased heart rate. If you have any of these, you may feel uncomfortable; however, these only last for a short time and most people manage well.

You will be closely monitored for depression and suicidal feelings. If at any point during the study you have any feelings/behaviors that make you feel concerned, a study investigator will be contacted. You may need to meet with him/her and/or your treatment team to ensure your safety.

POTENTIAL BENEFITS

You may or may not benefit from this study. If you participate in this study, you could benefit by stopping smoking.

ALTERNATIVES TO PARTICIPATION

This is a research study, and your participation is voluntary. Your alternative is not to take part in the study and seek services to help you to stop smoking at a site convenient to you or not at all. If you choose not to take part, your healthcare will not be affected.

All participants who are excluded from the study will be provided with information regarding the benefits of smoking cessation and will be counseled to discuss treatment for this with their primary HIV provider.

CONFIDENTIALITY AND ACCESS TO RECORDS

There is a risk of breach of confidentiality. To minimize this risk, all data will be coded with a unique ID number. All data including information from chart reviews or therapist reports will be labeled with the unique study ID. Only the study team will have access to the link between the unique ID and your name. Data will be stored in a secure location in a locked office within a locked cabinet and electronic data will be password protected.

The monitors, auditors, and the IRB will be granted direct access to your medical records for verification of the research procedures and data. By signing this document, you are authorizing this access.

The data from the study may be published. However, you will not be identified by name. People designated from the institutions where the study is being conducted and people from the sponsor will be allowed to inspect sections of your medical and research records related to the study. Everyone using study information will work to keep your personal information confidential. Your personal information will not be given out unless required by law.

A description of this clinical trial will be available on http://www.ClinicalTrials.gov, as required by U.S. Law. This Web site will not include information that can identify you. At most, the Web site will include a summary of the results. You can search this Web site at any time.

RIGHT TO WITHDRAW

Your participation in this study is voluntary. You do not have to take part in this research. You are free to withdraw your consent at any time. Refusal to take part or to stop taking part in the study will involve no penalty or loss of benefits you deserve. If you withdraw from this study, already collected data may not be removed from the study database. You will be asked whether the investigator can collect data from your routine medical care. If you agree, this data will be handled the same as research data.

CAN I BE REMOVED FROM THE RESEARCH?

The person in charge of the research study or the sponsor can discontinue a participant from the research study without their approval. Possible reasons for removal include but are not limited to failure to follow instructions of the research staff as well as if the person in charge decides that the research study is no longer in your best interest. The sponsor can also end the research study early. If this were to happen the study doctor will tell you about this and you will have the chance to ask questions.

Contact information for questions and concerns

If you decide to stop taking part in the interview, or if you have questions, concerns, or complaints, please contact Dr. Lillian Okui at telephone number +267-7164 2205.

COSTS TO PARTICIPANTS

It will not cost you anything to take part in this study.

PAYMENT TO PARTICIPANTS

There are up to 4 total study visits, and you will be reimbursed 80 Pula ($6 USD) for each of your study visits.

STUDY-RELATED INJURY

If you have an injury, promptly seek care from any healthcare provider. If you have an emergency, go to the nearest health center.

UNIVERSITY STATEMENT

The University of Maryland, Baltimore (UMB) is committed to providing participants in its research studies all rights due to them under State and federal law. You give up none of your legal rights by signing this consent form or by participating in this study. This study has been reviewed and approved by an Institutional Review Board (IRB). The IRB is a group of scientists, physicians, experts, and community representatives. The IRB's membership includes persons who are not affiliated with UMB and persons who do not conduct research studies.

If you have questions, concerns, complaints, or believe you have been harmed through participation in this study as a result of researcher negligence, you can contact members of the IRB or the Human Research Protections Office (HRPO) to ask questions, discuss problems or concerns, obtain information, or offer input about your rights as a research participant. The contact information for the IRB and the HRPO is:

University of Maryland, Baltimore

Institutional Review Board

Human Research Protections Office

620 W. Lexington Street, Second Floor

Baltimore, MD 21201

410-706-5037

Signing this consent form indicates that you have read this consent form (or have had it read to you), that your questions have been answered to your satisfaction, and that you voluntarily agree to participate in this research study. You will receive a copy of this signed consent form.

HEALTH RESEARCH AND DEVELOPMENT COMMITTEE (HRDC) IN BOTSWANA STATEMENT

If you have questions, concerns, complaints, or believe you have been harmed through participation in this study as a result of study team member negligence, you can contact members of the IRB or the staff of the HRDC to ask questions, discuss problems or concerns, obtain information, or offer input about your rights as a study participant. The contact information for the HRDC is:

K. Motlhanka, Health Research and Development Committee

Landline: +267 363 2018/ +267 363 2500

Email: kmotlhanka@gov.bw

STATEMENT OF CONSENT

Participant's Statement

I have read this consent form or had the information read to me. I have had the chance to discuss this research study with a study staff and have had my questions answered in a language that I understand. The risks and benefits have been explained to me. I understand

that my participation in this study is voluntary and that I may choose to withdraw at any time. I freely agree to participate in this research study. I understand that all efforts will be made to keep information regarding my personal identity confidential.

By signing this consent form, I have not given up any of my legal rights that I have as a participant in a research study.

1: I agree to participate in this research study: Yes No

2: I agree to be contacted for future research. Yes No

Participant's printed name: ___________________________________

Participant Signature: ___________________________ Date: _________________________

Researcher's Statement

I, the undersigned, have fully explained the research study to the participant named above and believe that the participant has understood and has willingly and freely given his/ her consent.

Researcher's name: ___________________________________

Signature: ___________________________ Date: _________________________

Role in the study: ____

Appendix A2: Research Consent Form – Intervention Phase

Protocol Title: Botswana Smoking Abstinence Reinforcement Trial (BSMART)

Study No: *HP-00102995*

Principal Investigators:

Dr. Manhattan Charurat	University of Maryland, Baltimore
Dr. Bontle Mbongwe	University of Botswana, Botswana
Dr. Seth Himmelhoch	University of Kentucky, Kentucky

Sponsor: *National Cancer Institute (NCI)*

CONCISE SUMMARY:

We are inviting you to take part in this research study. Participation in this research study is voluntary. You can choose to participate or not in this study. You can change your mind about participating at any time. Whatever choice you make, you will still receive the medical care you are receiving now, and you will not have to give up any of your rights.

This research study is trying to test one method to help people with HIV who smoke to stop smoking. If you choose to participate, you will:

- Need to answer some questions
- Undergo a physical examination
- Do a test where you blow into a machine and if you are found to be eligible
- Be prescribed a study medication called varenicline

Your participation at the clinic could take about 2 hours each visit. You will be asked to participate for about six months. The greatest risk of participating in this study is the loss of privacy and confidentiality such as your HIV status and side effects from taking a prescribed medication called Varenicline to assist you to stop smoking.

It will not cost you any money to participate in this research study. Prescribed medication will be paid for by the research study and will not cost you any money. Though you will be expected to come for 8 study visits, you will be provided transportation costs for four of them. You will receive 80 Pula ($6 USD) for transportation for study visits at which you will be asked a lot of questions.

The things I am about to tell you will give you more information about this study to help you decide if you would like to participate. Please listen carefully and ask the study staff to explain anything you do not understand. You will have a chance to ask questions before you make a decision to participate.

PURPOSE OF STUDY

The purpose of this study is to identify patients with HIV who smoke cigarettes and want to stop smoking and test a new way to help them stop smoking. We plan to include 750 people who have HIV and smoke cigarettes. We are working at 15 clinics.

PROCEDURES

a. Initial Screening

After you agree to participate and fill out a consent form, you will be asked several questions to see if you are eligible to be in this study. We will be asking you questions about your smoking habits and health history. During the screening appointment, you will be asked to provide medical history and complete a physical assessment. We will also have you blow into a carbon monoxide breath machine to measure the amount of carbon monoxide in your lungs from smoking. This will inform us of your smoking activity.

If you pass all the screening procedures, you will be asked to complete a baseline interview. This interview will ask you several questions. The topics will include: your smoking history, your mental health history and questions about your daily routine.

Female participants will be asked to take a pregnancy test during this visit and approximately monthly while taking study medication. We will ask female participants to use an acceptable form of contraception during the 12 weeks you are taking study medication. Condoms will be available from study staff.

b. Study Treatment

You will receive a special type of counseling and if you need it, treatment with Varenicline for approximately 12 weeks. Treatment participants who require varenicline will receive medication to take each day for 12 weeks. The study clinician will provide medical clearance and sign off on prescription orders. You will be supplied the medication if you are eligible. You will receive a weekly supply of medication for the first few weeks to ensure proper dosing and monitoring. During the first four weeks when you are taking the study medication, you will come in for a brief medication discussion every week to ensure you are doing well with your medication or if you are having any medication side effects or concerns.

Dosage:

If you are prescribed Varenicline, you will receive Varenicline in accordance with package labeling. You will begin varenicline one week before the date you have set to stop smoking.

For the first week, you will receive 0.5mg once a day for the first three days and then twice a day for the remaining four days. For the remaining eleven weeks, you will receive 1mg twice a day.

You will be in the study for a total of 24 weeks. You will be interviewed at baseline, week 4, week 12, and at approximately week 24. At week 1,2,3,4 and 8 there will also be a short check up on your vital signs, your medication adherence, your health and any medication side effects.

During week 4 and week 8 of the study, you will return to receive a four-week supply of medication. That means that you will come in for a brief 20-minute appointment. We will ask you about your mood, any side effects that you may be experiencing, check your vital signs and have you take a pregnancy test if applicable.

During visits at week 4, 12 and 24, you will be asked about the amount you are smoking and during your visits during weeks 12 and 24, we will have you blow into our Carbon Monoxide Breath machine to measure the amount of carbon monoxide in your airway and lungs.

All assessments will be administered in a confidential and private location by experienced staff.

WHAT ARE MY RESPONSIBILITIES IF I TAKE PART IN THIS RESEARCH?

You will be expected to come to the clinic for your appointments and answer questions on your smoking habits. During three visits, you will blow into a breath machine. If you are given varenicline, you should take the medication as prescribed after eating with a full glass of water. If any side-effects occur, you should report them to your clinician in a timely manner.

POTENTIAL RISKS/DISCOMFORTS:

Potential risks/side effects of varenicline

These include: nausea (up to 30%). In addition to nausea, other side effects occurring in greater than 5% of clinical trials and twice the rate in placebo were abnormal dreams, constipation, flatulence, and vomiting. Also in rare cases, changes in behavior, hostility, agitation, depressed mood, suicidal thoughts/actions, rash, and sensitivity reactions have occurred. We will closely monitor for depressive and other neuropsychiatric symptoms and have a careful plan in place for any exacerbation of symptoms or suicidality.

i. Risks associated with assessment interviews:

The risk from research interviews is minimal. During assessments you may be uncomfortable discussing your smoking habits or mental health history and treatments. You may become frustrated and tense if you encounter any difficulties while completing these measures. All interviewers are trained to recognize signs of distress or anxiety. You will be reminded that you can refuse to answer any question that makes you uncomfortable and may take breaks whenever you need.

ii. Risk of breach of confidentiality

There is a slight risk of breach of confidentiality (to minimize this we will use a unique ID study number for your information rather than your name)

iii. Risks associated with smoking cessation (Nicotine withdrawal symptoms)

There are additional risks in this study that occur when you stop smoking or are trying to stop smoking. One of these is the stress of and symptoms associated with nicotine withdrawal. Some people feel depressed, are unable to sleep, get angry easily, are anxious, have difficulty concentrating, are restless, or may have decreased heart rate. If you have any of these, you may feel uncomfortable; however, these only last for a short time and most people manage well.

You will be closely monitored for depression and suicidal feelings. If at any point during the study you endorse any concerning feelings/behaviors, a study investigator will be contacted. You may need to meet with him/her and/or your treatment team to ensure your safety.

If a female participant becomes pregnant during the period they are taking medication, they will stop participating in the study and will immediately stop taking the medication provided. They can speak to the study clinician about what they should do.

POTENTIAL BENEFITS

You may or may not benefit from this study. There is no guarantee that you will receive direct benefit from your participation in this study. If you participate in this study, you could benefit by stopping smoking.

ALTERNATIVES TO PARTICIPATION

This is a research study, and your participation is voluntary. Your alternative is not to take part in the study and seek services to help you to stop smoking at a site convenient to you or not at all. If you choose not to take part, your healthcare will not be affected.

All participants who are excluded from the study will be provided with information regarding the benefits of smoking cessation treatment and will be counseled to discuss treatment for this with their primary HIV provider.

CONFIDENTIALITY AND ACCESS TO RECORDS

There is a risk of breach of confidentiality. To minimize this risk all data will be coded with a unique ID number. All data including information from chart reviews or therapist reports will be labeled with the unique study ID. Only the study team will have access to the link between the unique ID and your name. Data will be stored in a secure location in a locked office within a locked cabinet and electronic data will be password protected.

The monitors, auditors, the IRB will be granted direct access to your medical records for verification of the research procedures and data. By signing this document, you are authorizing this access.

The data from the study may be published. However, you will not be identified by name. People designated from the institutions where the study is being conducted and people from the sponsor will be allowed to inspect sections of your medical and research records related to the study. Everyone using study information will work to keep your personal information confidential. Your personal information will not be given out unless required by law.

A description of this clinical trial will be available on http://www.ClinicalTrials.gov, as required by U.S. Law. This Web site will not include information that can identify you. At most, the Web site will include a summary of the results. You can search this Web site at any time.

RIGHT TO WITHDRAW

Your participation in this study is voluntary. You do not have to take part in this research. You are free to withdraw your consent at any time. Refusal to take part or to stop taking part in the study will involve no penalty or loss of benefits you deserve. If you withdraw from this study, already collected data may not be removed from the study database. You will be asked whether the investigator can collect data from your routine medical care. If you agree, this data will be handled the same as research data.

CAN I BE REMOVED FROM THE RESEARCH?

The person in charge of the research study or the sponsor can discontinue a participant from the research study without their approval. Possible reasons for removal include but are not limited to failure to follow instructions of the research staff as well as the person in charge decides that the research study is no longer in your best interest. The sponsor can also end the research study early. If this were to happen the study doctor will tell you about this and you will have the chance to ask questions.

Contact information for questions and concerns

If you decide to stop taking part in the interview, or if you have questions, concerns, or complaints, please contact Dr. Lillian Okui at telephone number +267-7164 2205.

COSTS TO PARTICIPANTS

It will not cost you anything to take part in this study.

PAYMENT TO PARTICIPANTS

There are up to 8 total study visits and you will be reimbursed 80 Pula ($6 USD) for four of your study visits.

STUDY-RELATED INJURY

If you have an injury, promptly seek care from any healthcare provider. If you have an emergency, go to the nearest health center.

UNIVERSITY STATEMENT

The University of Maryland, Baltimore (UMB) is committed to providing participants in its research studies all rights due to them under State and federal law. You give up none of your legal rights by signing this consent form or by participating in this study. This study has been reviewed and approved by an Institutional Review Board (IRB). The IRB is a group of scientists, physicians, experts, and community representatives. The IRB's membership includes persons who are not affiliated with UMB and persons who do not conduct research studies.

If you have questions, concerns, complaints, or believe you have been harmed through participation in this study as a result of researcher negligence, you can contact members of the IRB or the Human Research Protections Office (HRPO) to ask questions, discuss problems or concerns, obtain information, or offer input about your rights as a research participant. The contact information for the IRB and the HRPO is:

University of Maryland, Baltimore

Institutional Review Board

Human Research Protections Office

620 W. Lexington Street, Second Floor

Baltimore, MD 21201

410-706-5037

Signing this consent form indicates that you have read this consent form (or have had it read to you), that your questions have been answered to your satisfaction, and that you voluntarily agree to participate in this research study. You will receive a copy of this signed consent form.

HEALTH RESEARCH AND DEVELOPMENT COMMITTEE (HRDC) IN BOTSWANA STATEMENT

If you have questions, concerns, complaints, or believe you have been harmed through participation in this study as a result of study team member negligence, you can contact members of the IRB or the staff of the HRDC to ask questions, discuss problems or concerns, obtain information, or offer input about your rights as a study participant. The contact information for the HRDC is:

K. Motlhanka, Health Research and Development Committee

Landline: +267 363 2018/ +267 363 2500

Email: kmotlhanka@gov.bw

STATEMENT OF CONSENT

Participant's Statement

I have read this consent form or had the information read to me. I have had the chance to discuss this research study with a study staff and have had my questions answered in a

language that I understand. The risks and benefits have been explained to me. I understand that my participation in this study is voluntary and that I may choose to withdraw at any time. I freely agree to participate in this research study. I understand that all efforts will be made to keep information regarding my personal identity confidential.

By signing this consent form, I have not given up any of my legal rights that I have as a participant in a research study.

I agree to participate in this research study: Yes No

Participant's printed name: ___________________________________

Participant Signature: ___________________________ Date: _________________________

Researcher's Statement

I, the undersigned, have fully explained the research study to the participant named above and believe that the participant has understood and has willingly and freely given his/ her consent.

Researcher's name: ___________________________________

Signature: ___________________________ Date: _________________________

Role in the study: ____

Appendix A3: Research Consent Form- Focus Group Discussion with Lay Health Workers

Protocol Title: Botswana Smoking Abstinence Reinforcement Trial (BSMART)

Study No.: *HP-00102995*

Principal Investigators:

Dr. Manhattan Charurat University of Maryland, Baltimore

Dr. Bontle Mbongwe University of Botswana, Botswana

Dr. Seth Himelhoch University of Kentucky, Kentucky

Sponsor: *National Cancer Institute (NCI)*

CONCISE SUMMARY:

We are inviting you to take part in this research study. Participation in this research study is voluntary. You can choose to participate or not in this study. You can change your mind about participating at any time. Whatever choice you make will not affect your employment and you will not need to give up any of your benefits or rights.

This research study is trying to test one method to help people with HIV who smoke to stop smoking. We want to talk with you about this because you have been or will be trained to

- identify participants who were eligible to take part in the BSMART study,
- counsel participants and prepare them to quit smoking.
- work with them to develop a plan to change their smoking habits and
- refer them to nurse prescribers when needed.

(For the post-BSMART Intervention FGD) We want to find out from you how prepared you felt to counsel participants and work with them to develop a plan to change their smoking habits. We also want to find out how easy (or difficult) it was for you to include this program in your work at the clinic. We want to learn from you about any challenges you had and get ideas from you on the best way to do it. You will need to come for two focus group sessions.

The biggest risk you may have if you participate in this interview is that people may get to know that you are living with HIV or that you work in a clinic that provides care to people living with HIV.

It will not cost you any money to participate in this interview. Instead, you will receive 80 Pula (6$ USD) for transportation for participating in the focus group discussion.

The things I am about to tell you will give you more information about this study to help you decide if you would like to participate.

Please listen carefully and ask the study staff to explain anything you do not understand. You will have a chance to ask questions before you make a decision to participate.

PURPOSE OF STUDY

The purpose of this study is to identify patients with HIV who smoke cigarettes and want to stop smoking and test a new way to help them stop smoking. We plan to include 750 people who have HIV and smoke cigarettes. We are working at 15 clinics.

You have been selected to participate in this interview because you are a lay health worker working with participants who live with HIV and smoke. Your participation in this discussion is important because you will help us identify the challenges and successes of a program to help people to stop smoking.

This study is being conducted by the University of Maryland, Baltimore, University of Botswana and the Botswana University of Maryland Medicine Health Initiative (BUMMHI), who currently collaborate on Accelerating Botswana through the Last Mile to Epidemic Control (ABLE) project providing support for HIV care and treatment in 53 high-volume facilities across Botswana, including the 15 selected and 4 reserve sites proposed for this study.

PROCEDURES

After you agree to participate and fill out a consent form, you will be asked questions to help us plan the best way to give patients this program.

For confidentiality, we will assign a unique code to you to identify you and only this number will be written to identify your answers.

We will use 2 voice recorders (no video), one as a backup, to record our conversation so that we can listen to the recording in case we miss anything you say in our notetaking during the conversation. The audio record will be transcribed and stored in a password-protected computer. After transcription, both files will be destroyed. If you do not want us to record your discussion, you will not be able to take part in the focus group discussions.

We are not looking for specific responses; there are no right or wrong answers to questions. We will not ask you to share any stories about your personal life. When we ask questions, it is fine not to answer any question you do not want to answer. You may also choose to stop answering questions at any time.

WHAT ARE MY RESPONSIBILITIES IF I TAKE PART IN THIS RESEARCH?

You will be expected to participate in two focus group discussions.

POTENTIAL RISKS/DISCOMFORTS:

There is a risk of loss of privacy as this is a focus group discussion. We will ask participants to keep information from this discussion confidential and to not discuss outside of this group discussion. Additionally, no names will be used during the discussion and transcription.

POTENTIAL BENEFITS

Your participation in this interview will help us to plan the smoking cessation program in a way that will be easier for you to provide to patients and in a way that will not take too much of your time. You will help us to provide a program that can help people to stop smoking.

ALTERNATIVES TO PARTICIPATION

This is a research study, and your participation is voluntary. You can ask questions at any time. Your alternative is not to take part in the focus group discussion. If you choose not to take part, your employment will not be affected, and you will not lose any of your benefits or rights.

CONFIDENTIALITY AND ACCESS TO RECORDS

There is a risk of breach of confidentiality. To minimize this risk all data will be labeled with a unique study ID. Only the study team will have access to any link between the unique ID and the participant's name. Data will be stored in a secure location in a locked office within a locked cabinet and electronic data will be password protected.

The monitors, auditors, and the IRB will be granted direct access to your study records for verification of the research procedures and data. By signing this document, you are authorizing this access.

"The data from the study may be published. However, you will not be identified by name. People designated from the institutions where the study is being conducted and people from the sponsor will be allowed to inspect sections of your medical and research records related to the study. Everyone using study information will work to keep your personal information confidential. Your personal information will not be given out unless required by law."

A description of this clinical trial will be available on http://www.ClinicalTrials.gov, as required by U.S. Law. This Web site will not include information that can identify you. At most, the website will include a summary of the results. You can search this Web site at any time.

RIGHT TO WITHDRAW

Your participation in the focus group discussions is voluntary. You do not have to take part in this research. You are free to withdraw your consent at any time. Refusal to take part or to stop taking part in the study will involve no penalty or loss of benefits or employment to which you are otherwise entitled. If you withdraw from this study, already collected data may not be removed from the study database.

CAN I BE REMOVED FROM THE RESEARCH?

The person in charge of the research study or the sponsor can remove you from the research study without your approval. Possible reasons for removal include but are not limited to failure to follow instructions of the research staff as well as if the person in charge decides that the research study is no longer in your best interest. The sponsor can also end the research study early. If this were to happen the study coordinator will tell you about this and you will have the chance to ask questions.

Contact information for questions and concerns:

If you decide to stop taking part in the interview, or if you have questions, concerns, or complaints, please contact Dr. Lillian Okui at telephone number +267-7164 2205.

COSTS TO PARTICIPANTS

It will not cost you anything to take part in the focus group discussions.

PAYMENT TO PARTICIPANTS

You will be reimbursed 80 Pula (6$ USD) for travel expenses incurred to participate in each focus group discussion.

STUDY-RELATED INJURY

If you have an injury, promptly seek care from any healthcare provider. If you have an emergency, go to the nearest health center.

UNIVERSITY STATEMENT

The University of Maryland, Baltimore (UMB) is committed to providing participants in its research studies all rights due to them under State and federal law. You give up none of your legal rights by signing this consent form or by participating in this study. This study has been reviewed and approved by an Institutional Review Board (IRB). The IRB is a group of scientists, physicians, experts, and community representatives. The IRB's membership includes persons who are not affiliated with UMB and persons who do not conduct research studies.

If you have questions, concerns, complaints, or believe you have been harmed through participation in this study as a result of researcher negligence, you can contact members of the IRB or the Human Research Protections Office (HRPO) to ask questions, discuss problems or concerns, obtain information, or offer input about your rights as a research participant. The contact information for the IRB and the HRPO is:

University of Maryland, Baltimore

Institutional Review Board

Human Research Protections Office

620 W. Lexington Street, Second Floor

Baltimore, MD 21201

410-706-5037

Signing this consent form indicates that you have read this consent form (or have had it read to you), that your questions have been answered to your satisfaction, and that you voluntarily agree to participate in this research study. You will receive a copy of this signed consent form.

HEALTH RESEARCH AND DEVELOPMENT COMMITTEE (HRDC) IN BOTSWANA STATEMENT

If you have questions, concerns, complaints, or believe you have been harmed through participation in this study as a result of study team member negligence, you can contact members of the IRB or the staff of the HRDC to ask questions, discuss problems or concerns, obtain information, or offer input about your rights as a study participant. The contact information for the HRDC is:

K. Motlhanka, Health Research and Development Committee

Landline: +267 363 2018/ +267 363 2500

Email: kmotlhanka@gov.bw

STATEMENT OF CONSENT

Participant's Statement

I have read this consent form or had the information read to me. I have had the chance to discuss this research study with a study staff and have had my questions answered in a language that I understand. The risks and benefits have been explained to me. I understand that my participation in this study is voluntary and that I may choose to withdraw at any time. I freely agree to participate in this research study. I understand that all efforts will be made to keep information regarding my personal identity confidential.

By signing this consent form, I have not given up any of my legal rights that I have as a participant in a research study.

I agree to participate in this research study: Yes No

Participant's printed name: ___________________________________

Participant Signature: ___________________________ Date: _________________________

Researcher's Statement

I, the undersigned, have fully explained the research study to the participant named above and believe that the participant has understood and has willingly and freely given his/ her consent.

Researcher's name: ___________________________________

Signature: ___________________________ Date: _________________________

Role in the study: ____

Appendix A4: Research Consent Form - Focus Group Discussion with Nurse Prescribers/ Dispensers

Protocol Title: Botswana Smoking Abstinence Reinforcement Trial (BSMART)

Study No.: *HP-00102995*

Principal Investigators:

Dr. Manhattan Charurat	University of Maryland, Baltimore
Dr. Bontle Mbongwe	University of Botswana, Botswana
Dr. Seth Himelhoch	University of Kentucky, Kentucky

Sponsor: *National Cancer Institute (NCI)*

CONCISE SUMMARY:

We are inviting you to take part in this research study. Participation in this research study is voluntary. You can choose to participate or not in this study. You can change your mind about participating at any time. Whatever choice you make will not affect your employment and you will not need to give up any of your benefits or rights.

This research study is trying to test one method to help people with HIV who smoke to stop smoking. We want to talk with you about this because you will be or have been trained to

- identify participants who were eligible to take varenicline,
- prescribe varenicline
- monitor the adherence of participants to varenicline and
- identify and manage side-effects to varenicline and you have provided these services for some months.

(For the post-BSMART Intervention FGD) We want to find out from you how prepared you felt to dispense varenicline and complete the data collection tools you were expected to fill and how easy it was for you to include this program into your work at the clinic. We want to learn from you about any challenges you had and get ideas from you on the best way to do it. You will only need to come for one focus group session.

The biggest risk you may have if you participate in this interview is that people may get to know that you provide care to people living with HIV.

It will not cost you any money to participate in this interview. Instead, you will receive 80 Pula ($6 USD) for transportation for participating in each focus group discussion.

The things I am about to tell you will give you more information about this study to help you decide if you would like to participate.

Please listen carefully and ask the study staff to explain anything you do not understand. You will have a chance to ask questions before you make a decision to participate.

PURPOSE OF STUDY

The purpose of this study is to identify patients with HIV who smoke cigarettes and want to stop smoking and test a new way to help them stop smoking. We plan to include 750 people who have HIV and smoke cigarettes. We are working at 15 clinics.

You have been selected to participate in this interview because you are a clinician working with participants who live with HIV and smoke. Your participation in this discussion is important because you will help us identify the challenges and successes of a program to help people to stop smoking.

This study is being conducted by the University of Maryland, Baltimore, University of Botswana and the Botswana University of Maryland Medicine Health Initiative (BUMMHI), who currently collaborate on Accelerating Botswana through the Last Mile to Epidemic Control (ABLE) project providing support for HIV care and treatment in 53 high-volume facilities across Botswana, including the 15 selected and 4 reserve sites proposed for this study.

PROCEDURES

After you agree to participate and fill out a consent form, you will be asked questions to help us plan the best way to give patients this program.

For confidentiality, we will assign a unique code to you to identify you and only this number will be written to identify your answers.

We will use two voice recorders (no video), one as a backup, to record our conversation so that we can listen to the recording in case we miss anything you say in our notetaking during the conversation. The audio record will be transcribed and stored in a password-protected computer. After transcription, both files will be destroyed. If you do not want us to record your discussion, you will not be able to take part in this focus group discussion.

We are not looking for specific responses; there are no right or wrong answers to questions. We will not ask you to share any stories about your personal life. When we ask questions, it is fine not to answer any question you do not want to answer. You may also choose to stop answering questions at any time.

WHAT ARE MY RESPONSIBILITIES IF I TAKE PART IN THIS RESEARCH?

You will be expected to participate in two focus group discussions.

POTENTIAL RISKS/DISCOMFORTS:

There is a risk of loss of privacy as this is a focus group discussion. We will ask participants to keep information from this discussion confidential and to not discuss outside of this group discussion. Additionally, no names will be used during the discussion and transcription.

POTENTIAL BENEFITS

Your participation in this interview will help us to plan the smoking cessation program in a way that will be easier for you to provide to patients and in a way that will not take too much of your time. You will help us to provide a program that can help people to stop smoking.

ALTERNATIVES TO PARTICIPATION

This is a research study, and your participation is voluntary. You can ask questions at any time. Your alternative is not to take part in the focus group discussion. If you choose not to take part, your employment will not be affected, and you will not lose any of your benefits or rights.

CONFIDENTIALITY AND ACCESS TO RECORDS

There is a risk of breach of confidentiality. To minimize this risk, all data will be coded with a unique ID number. Only the study team will have access to the link between the unique ID and your name. Data will be stored in a secure location in a locked office within a locked cabinet and electronic data will be password protected.

The monitors, auditors, and the IRB will be granted direct access to your medical records for verification of the research procedures and data. By signing this document, you are authorizing this access.

The data from the study may be published. However, you will not be identified by name. People designated from the institutions where the study is being conducted and people from the sponsor will be allowed to inspect sections of your research records related to the study. Everyone using study information will work to keep your personal information confidential. Your personal information will not be given out unless required by law.

A description of this clinical trial will be available on http://www.ClinicalTrials.gov, as required by U.S. Law. This Web site will not include information that can identify you. At most, the Web site will include a summary of the results. You can search this Web site at any time.

RIGHT TO WITHDRAW

Your participation in the focus group discussion is voluntary. You do not have to take part in this research. You are free to withdraw your consent at any time. Refusal to take part or to stop taking part in the study will involve no penalty or loss of benefits or employment to which you are otherwise entitled. If you withdraw from this study, the already collected data may not be removed from the study database.

CAN I BE REMOVED FROM THE RESEARCH?

The person in charge of the research study or the sponsor can remove you from the research study without your approval. Possible reasons for removal include but are not limited to failure to follow instructions of the research staff as well as if the person in charge decides that the research study is no longer in your best interest. The sponsor can also end the research study early. If this were to happen the study coordinator will tell you about this and you will have the chance to ask questions.

Contact information for questions and concerns

If you decide to stop taking part in the interview, or if you have questions, concerns, or complaints, please contact Dr. Lillian Okui at telephone number +267-7164 2205.

COSTS TO PARTICIPANTS

It will not cost you anything to take part in this study.

PAYMENT TO PARTICIPANTS

You will be reimbursed 80 Pula ($6 USD) for travel expenses incurred to participate in each focus group discussion.

STUDY-RELATED INJURY

If you have an injury, promptly seek care from any healthcare provider. If you have an emergency, go to the nearest health center.

UNIVERSITY STATEMENT

The University of Maryland, Baltimore (UMB) is committed to providing participants in its research studies all rights due to them under State and federal law. You give up none of your legal rights by signing this consent form or by participating in this study. This study has been reviewed and approved by an Institutional Review Board (IRB). The IRB is a group of scientists, physicians, experts, and community representatives. The IRB's membership includes persons who are not affiliated with UMB and persons who do not conduct research studies.

If you have questions, concerns, complaints, or believe you have been harmed through participation in this study as a result of researcher negligence, you can contact members of the IRB or the Human Research Protections Office (HRPO) to ask questions, discuss problems or concerns, obtain information, or offer input about your rights as a research participant. The contact information for the IRB and the HRPO is:

University of Maryland, Baltimore

Institutional Review Board

Human Research Protections Office

620 W. Lexington Street, Second Floor

Baltimore, MD 21201

410-706-5037

Signing this consent form indicates that you have read this consent form (or have had it read to you), that your questions have been answered to your satisfaction, and that you voluntarily agree to participate in this research study. You will receive a copy of this signed consent form.

HEALTH RESEARCH AND DEVELOPMENT COMMITTEE (HRDC) IN BOTSWANA STATEMENT

If you have questions, concerns, complaints, or believe you have been harmed through participation in this study as a result of study team member negligence, you can contact members of the IRB or the staff of the HRDC to ask questions, discuss problems or concerns,

obtain information, or offer input about your rights as a study participant. The contact information for the HRDC is:

K. Motlhanka, Health Research and Development Committee

Landline: +267 363 2018/ +267 363 2500

Email: kmotlhanka@gov.bw

STATEMENT OF CONSENT

Participant's Statement

I have read this consent form or had the information read to me. I have had the chance to discuss this research study with a study staff and have had my questions answered in a language that I understand. The risks and benefits have been explained to me. I understand that my participation in this study is voluntary and that I may choose to withdraw at any time. I freely agree to participate in this research study. I understand that all efforts will be made to keep information regarding my personal identity confidential.

By signing this consent form, I have not given up any of my legal rights that I have as a participant in a research study.

I agree to participate in this research study: Yes No

Participant's printed name: ___________________________________

Participant Signature: ___________________________ Date: _________________________

Researcher's Statement

I, the undersigned, have fully explained the research study to the participant named above and believe that the participant has understood and has willingly and freely given his/ her consent.

Researcher's name: ___________________________________

Signature: ___________________________ Date: _________________________

Role in the study: ____

Appendix A5: Research Consent Form - Collection of cost data from LHWs and NPDs

Protocol Title: Botswana Smoking Abstinence Reinforcement Trial (BSMART)

Study No.: *HP-00102995*

Principal Investigators:

Dr. Manhattan Charurat	University of Maryland, Baltimore
Dr. Bontle Mbongwe	University of Botswana, Botswana
Dr. Seth Himelhoch	University of Kentucky, Kentucky

Sponsor: *National Cancer Institute (NCI)*

CONCISE SUMMARY:

We are inviting you to take part in this research study. Participation in this research study is voluntary. You can choose to participate or not in this study. You can change your mind about participating at any time. Whatever choice you make will not affect your employment and you will not need to give up any of your benefits or rights.

This research study is trying to test one method to help people with HIV who smoke to stop smoking, and to estimate the cost of providing these services to clients who need them. To help us calculate the cost of the program, we will need to collect information on how much you earn and how much time you spend with each participant as you are providing them with services to help them to stop smoking.

The biggest risk you may have if you participate in this study is that people may get to know that you provide care to people living with HIV.

It will not cost you any money to provide the information we need.

The things I am about to tell you will give you more information about this study to help you decide if you would like to participate. Please listen carefully and ask the study staff to explain anything you do not understand. You will have a chance to ask questions before you make a decision to participate.

PURPOSE OF STUDY

The purpose of this research study is to identify patients who have HIV and smoke cigarettes who want to stop smoking and test a new way to help them stop smoking. We plan to include 750 people who have HIV and smoke cigarettes. We are working at 15 clinics. We also need to estimate how much it costs to provide these services to participants at your facility.

You have been selected to participate in this research study because you are a LHW or clinician working with participants who live with HIV and smoke. Your participation in this study is important because you are one of the hospital staff providing smoking cessation services to participants and we need to estimate how much it costs for health workers like you to provide these services to clients.

This study is being conducted by the University of Maryland, Baltimore, University of Botswana and the Botswana University of Maryland Medicine Health Initiative (BUMMHI), who currently collaborate on Accelerating Botswana through the Last Mile to Epidemic Control (ABLE) project providing support for HIV care and treatment in 53 high-volume facilities across Botswana, including the 15 selected and 4 reserve sites proposed for this study.

PROCEDURES

After you agree to participate and fill out a consent form, you will have your interactions with participants timed at every study visit. Research staff will fill out the time the visit starts on the participant follow-up form and the time the visit ends. Research staff will also collect salary information from the accounts department at your facility.

WHAT ARE MY RESPONSIBILITIES IF I TAKE PART IN THIS RESEARCH?

You will need to provide standard of care or the BSMART intervention to participants (depending on the study phase being implemented at your facility at the time).

POTENTIAL RISKS/DISCOMFORTS:

There is a risk of loss of privacy and discomfort at having the time you spend with each participant recorded.

POTENTIAL BENEFITS

You will be trained in motivational interviewing and how best to counsel people who smoke to encourage them to quit. You will also be trained to fill out data collection tools.

For nurse prescribers, you will also be trained on how to screen participants to identify those eligible to use varenicline, and how to monitor participants for adherence and side-effects.

ALTERNATIVES TO PARTICIPATION

This is a research study, and your participation is voluntary. You can ask questions at any time. Your alternative is not to take part in the study. If you choose not to take part, your employment will not be affected, and you will not lose any of your benefits or rights.

CONFIDENTIALITY AND ACCESS TO RECORDS

There is a risk of breach of confidentiality. To minimize this risk, all data will be coded with a unique ID number. Only the study team will have access to the link between the unique ID and your name. Data will be stored in a secure location in a locked office within a locked cabinet and electronic data will be password protected.

The monitors, auditors, and the IRB will be granted direct access to your study records for verification of the research procedures and data. By signing this document, you are authorizing this access.

The data from the study may be published. However, you will not be identified by name. People designated from the institutions where the study is being conducted and people from the sponsor will be allowed to inspect sections of your research records related to the study. Everyone using study information will work to keep your personal information confidential. Your personal information will not be given out unless required by law.

A description of this clinical trial will be available on http://www.ClinicalTrials.gov, as required by U.S. Law. This Web site will not include information that can identify you. At most, the Web site will include a summary of the results. You can search this Web site at any time.

RIGHT TO WITHDRAW

Your participation in this study is voluntary. You do not have to take part in this research. You are free to withdraw your consent at any time. Refusal to take part or to stop taking part in the study will involve no penalty or loss of benefits or employment to which you are otherwise entitled. If you withdraw from this study, already collected data may not be removed from the study database.

CAN I BE REMOVED FROM THE RESEARCH?

The person in charge of the research study or the sponsor can remove you from the research study without your approval. Possible reasons for removal include but are not limited to failure to follow instructions of the research staff as well as if the person in charge decides that the research study is no longer in your best interest. The sponsor can also end the research study early. If this were to happen the study coordinator will tell you about this and you will have the chance to ask questions.

Contact information for questions and concerns

If you decide to stop taking part in the interview, or if you have questions, concerns, or complaints, please contact Dr. Lillian Okui at telephone number +267-7164 2205.

COSTS TO PARTICIPANTS

It will not cost you anything to take part in this study.

PAYMENT TO PARTICIPANTS

You will not receive any payment for participating in the study.

STUDY-RELATED INJURY

If you have an injury, promptly seek care from any healthcare provider. If you have an emergency, go to the nearest health center.

UNIVERSITY STATEMENT

The University of Maryland, Baltimore (UMB) is committed to providing participants in its research studies all rights due to them under State and federal law. You give up none of your legal rights by signing this consent form or by participating in this study. This study has been reviewed and approved by an Institutional Review Board (IRB). The IRB is a group of scientists, physicians, experts, and community representatives. The IRB's membership includes persons who are not affiliated with UMB and persons who do not conduct research studies.

If you have questions, concerns, complaints, or believe you have been harmed through participation in this study as a result of researcher negligence, you can contact members of the IRB or the Human Research Protections Office (HRPO) to ask questions, discuss problems or concerns, obtain information, or offer input about your rights as a research participant. The contact information for the IRB and the HRPO is:

University of Maryland, Baltimore

Institutional Review Board

Human Research Protections Office

620 W. Lexington Street, Second Floor

Baltimore, MD 21201

410-706-5037

Signing this consent form indicates that you have read this consent form (or have had it read to you), that your questions have been answered to your satisfaction, and that you voluntarily agree to participate in this research study. You will receive a copy of this signed consent form.

HEALTH RESEARCH AND DEVELOPMENT COMMITTEE (HRDC) IN BOTSWANA STATEMENT

If you have questions, concerns, complaints, or believe you have been harmed through participation in this study as a result of study team member negligence, you can contact members of the IRB or the staff of the HRDC to ask questions, discuss problems or concerns, obtain information, or offer input about your rights as a study participant. The contact information for the HRDC is:

K. Motlhanka, Health Research and Development Committee

Landline: +267 363 2018/ +267 363 2500

Email: kmotlhanka@gov.bw

STATEMENT OF CONSENT

Participant's Statement

I have read this consent form or had the information read to me. I have had the chance to discuss this research study with a study staff and have had my questions answered in a language that I understand. The risks and benefits have been explained to me. I understand that my participation in this study is voluntary and that I may choose to withdraw at any time. I freely agree to participate in this research study. I understand that all efforts will be made to keep information regarding my personal identity confidential.

By signing this consent form, I have not given up any of my legal rights that I have as a participant in a research study.

I agree to participate in this research study: Yes No

Participant's printed name: ___________________________________

Participant Signature: ___________________________ Date: _________________________

Researcher's Statement

I, the undersigned, have fully explained the research study to the participant named above and believe that the participant has understood and has willingly and freely given his/ her consent.

Researcher's name: ___________________________________

Signature: ___________________________ Date: _________________________

Role in the study: ____

Appendix B: Baseline, Smoking Questionnaires and Readiness Tools

Appendix BI: BSMART Screening Tool

**Researcher ID:**	**Participant ID:**
**District:**	**Facility:**
**Date: (dd/mm/yyyy)**	

Eligibility Checklist

**Inclusion Criteria**		
**Yes**	**No**	
		Confirmed chart diagnosis of HIV and receiving treatment at the participating HIV clinic
		Current self-reported daily smoker
		Age 18 or older
		Willing and able to provide informed consent in English or Setswana
**Exclusion Criteria**		
**Yes**	**No**	
		Current pregnancy or nursing

For use during screening of subjects

Name of subject　　　　　　　　　　　　Date Screened

Reviewed and subject acceptable for study: _______ Yes ______ No

Signature of Principal Investigator　　　　　　　　　　　　Date

Appendix B2: BSMART Demographics Form

**Researcher ID:**	**Participant ID:**
**District:**	**Facility:**
**Date: (dd/mm/yyyy)**	

1. **What is your date of birth? _________________ Or How old are you? _______**Age in years

2. **Sex**

☐ Male

☐ Female

☐ Other (Please specify) ___________________________

3. **How much education have you completed?**

☐ None (Never attended school)

☐ Primary school

☐ Secondary School

☐ Senior Secondary School

☐ Tertiary

4. **What is your marital status?**

☐ Single (Never married)

☐ Married/ Living with a partner

☐ Previously married (Separated/ Widowed/ Divorced)

5. **What is your employment status?**

☐ Employed

☐ Unemployed

☐ Retired

☐ Other (please specify) _______________________________

Appendix B3: Intake Smoking Questionnaire

BSMART Intake Smoking History Questionnaire

**Researcher ID:**	**Participant ID:**
**District:**	**Facility:**
**Date: (dd/mon/yyyy)**	

	**Question**	**Response**	
1.	How many days of the week do you smoke?	☐ 7<br>☐ 6<br>☐ 5<br>☐ 4<br>☐ 3<br>☐ 2<br>☐ 1	
Determining nicotine dependence (Fagerstrom Nicotine Dependence Scale)			
		**Answers**	**Points**
2a.	How soon after you wake up do you smoke your first cigarette?	Within 5 mins<br>6 – 30 mins<br>31 – 60 mins<br>After 60 mins	3<br>2<br>1<br>0
2b.	Do you find it difficult to stop yourself from smoking in places where it is forbidden eg. In church, at the library, in waiting rooms at the bus stop or train station, at government offices?	Yes<br>No	1<br>0
2c.	Which cigarette would you hate to give up the most?	The first one in The morning<br>All others	1<br>0

2d.	How many cigarettes do you smoke per day?	10 or less<br>11 – 20<br>21 – 30<br>31 or more	0<br>1<br>2<br>3
2e.	Do you smoke more frequently during the first hours after waking up than during the rest of the day?	Yes<br>No	1<br>0
2f.	Do you smoke if you are so ill that you are in bed most of the day?	Yes<br>No	1<br>0
Add up the responses to all items in Q2. A score of 5 or more shows significant dependence while a score of 4 or less shows low to moderate dependence			
Now we are going to ask you questions about times you may have tried to quit smoking			
3.	How often have you used each of the following in your efforts to quit smoking? (Answer each item)<br>1 = Never<br>2 = Seldom<br>3 = Occasionally<br>4 = Frequently<br>5 = Repeatedly	**1 2 3 4 5**	
	▪ Quitting all at once	1 2 3 4 5	
	▪ Gradually cutting down	1 2 3 4 5	
	▪ Nicorette gum	1 2 3 4 5	
	▪ Nicotine patch	1 2 3 4 5	
	▪ Nasal sprays	1 2 3 4 5	
	▪ Counseling (Pharmacist or counselor)	1 2 3 4 5	
	▪ Stop smoking support group	1 2 3 4 5	
	▪ Electronic cigarettes	1 2 3 4 5	
	▪ Self-help manuals or books	1 2 3 4 5	
	▪ Other____		

4.	Since you started smoking regularly, have you ever quit for a period of at least 24 hours?<br>**If No, skip to question 7.**	☐ Yes<br>☐ No
5.	In the last year, how many times have you quit for at least 24 hours? (If more than 9 times, put 9)	____________ times
6.	After your most recent quit attempt, how long did you stay off cigarettes?	☐ 1-2 days<br>☐ A few days<br>☐ A week<br>☐ Two to four weeks<br>☐ More than four weeks
I will now ask you a few questions about your smoking history		
7.	To the best of your knowledge, categorize the use of cigarettes by the following people in your life	1 = Smoker<br>2 = Ex-smoker<br>3 = Never smoked<br>4 = Not applicable
	▪ Father<br>▪ Mother<br>▪ Spouse<br>▪ Best friend<br>▪ Closest work associate	1 2 3 4<br>1 2 3 4<br>1 2 3 4<br>1 2 3 4<br>1 2 3 4
8.	How old were you when you started smoking?	_____________ years
9.	How many years have you smoked? (Total number of years)	______________ years
10.	What type of tobacco do you usually smoke?<br>(**Select all that apply**)<br>▪ Manufactured cigarettes<br>▪ Homemade (roll your own)<br>▪ Cigars<br>▪ e-cigarettes<br>▪ Waterpipe (bubbly)	 <br>☐<br>☐<br>☐<br>☐<br>☐

	▪ Other (please specify)	____________________
	For people who smoke manufactured cigarettes, what brand of cigarette do you usually smoke?	Write in brand.<br>____________________
11.	In addition to smoking cigarettes, do you use snuff?<br>**If No, Continue to Q13.**	☐ Yes<br>☐ No
12.	How often do you use snuff?	☐ Daily<br>☐ Every few days<br>☐ Once a week<br>☐ Every few weeks<br>☐ Once a month<br>☐ Less than monthly (rarely)
I will now ask you some questions about how confident you are about quitting cigarette smoking		
13.	How confident are you that you will be able to stop smoking at this time?<br>**1 = Not at all confident**<br>**2 = Somewhat confident**<br>**3 = Moderately confident**<br>**4 = Very confident**<br>5 **= Extremely confident**	1 2 3 4 5
For each question choose from the following alternatives and circle the choice in the right-hand margin with the code number that best suits you.<br>**1 = Never 2 = Almost Never 3 = Sometimes 4 = Fairly Often 5 = Very Often**		
14.	In the past 30 days, how often have you felt confident about your ability to handle your personal problems?	1 2 3 4 5
15.	In the last 30 days, how often have you felt that you were unable to control the important things in your life?	1 2 3 4 5
16.	In the last 30 days, how often have you felt things were going your way?	1 2 3 4 5

17.	In the last 30 days, how often have you felt difficulties were piling up so high that you could not overcome them?	1 2 3 4 5

Smoking situations: Temptation

Listed below are situations that lead some people to smoke. We would like to know how tempted you may be to smoke in these situations.

Please answer the following questions by using a 5-point scale with **5 = Extremely tempted 4 = Very tempted 3 = Moderately tempted 2 = Not very tempted 1 = Not at all tempted**

18. How tempted would you be to smoke in this situation?

	At a bar or cocktail lounge having a drink	1 2 3 4 5
	When I am desiring a cigarette	1 2 3 4 5
	When things are just not going the way I want and I am frustrated	1 2 3 4 5
	With my spouse or close friend who is smoking	1 2 3 4 5
	When there are arguments and conflicts with my family	1 2 3 4 5
	When I am happy and celebrating	1 2 3 4 5
	When I am very angry about something or someone	1 2 3 4 5
	When I would experience an emotional crisis, such as an accident or death in the family	1 2 3 4 5
	When I see someone smoking and enjoying it	1 2 3 4 5
	When I realize that quitting smoking is an extremely difficult task for me	1 2 3 4 5
	When I am craving a cigarette	1 2 3 4 5
	When I first get up in the morning	1 2 3 4 5
	When I need to lift my spirits	1 2 3 4 5
	With friends at a party	1 2 3 4 5
	When I am extremely anxious or depressed	1 2 3 4 5
	When I am stressed	1 2 3 4 5

	When I realize I haven't smoked for a while	1 2 3 4 5

Pros and Cons

The following statements represent different opinions about smoking.

19. Please rate HOW IMPORTANT each statement is to you according to the following 5-point scale with

5 = Extremely important 4 = Very Important 3 = Moderately important 2 = Slightly important

1 = Not important

	Smoking cigarettes is pleasurable	1 2 3 4 5
	My smoking affects the health of others	1 2 3 4 5
	I like the image of a cigarette smoker	1 2 3 4 5
	Others close to me would suffer if I became ill from smoking	1 2 3 4 5
	I am relaxed and therefore more pleasant when smoking	1 2 3 4 5
	Because I continue to smoke, some people I know think I lack character	1 2 3 4 5
	If I try to stop smoking, I'll be irritable and a pain to be around	1 2 3 4 5
	Smoking cigarettes is hazardous to my health	1 2 3 4 5
	My family and friends like me better when I am happily smoking than when I am miserable trying to quit	1 2 3 4 5
	I'm embarrassed to have to smoke	1 2 3 4 5
	I like myself better when I smoke	1 2 3 4 5
	My cigarette smoking bothers other people	1 2 3 4 5
	Smoking helps me concentrate and do better work	1 2 3 4 5
	People think I'm foolish for ignoring the warnings about cigarette smoking	1 2 3 4 5
	Smoking cigarettes relieves tension	1 2 3 4 5
	People close to me disapprove of my smoking	1 2 3 4 5
	By continuing to smoke, I feel I am making my own decisions	1 2 3 4 5
	I'm foolish to ignore the warnings about cigarettes	1 2 3 4 5

	After not smoking for a while, a cigarette makes me feel great	1 2 3 4 5
	I would be more energetic right now if I didn’t smoke	1 2 3 4 5

Smoking situations: Confidence

Listed below are situations that lead some people to smoke. We should like to know how confident you are that you would not smoke in these situations.

Please answer the following question by using a 5-point scale with **5 = extremely confident; 4 = very confident; 3 = moderately confident; 2 = not very confident and 1 = not at all confident**

20. How confident are you that you would NOT smoke in this situation?

	At a bar or cocktail lounge having a drink	1 2 3 4 5
	When I am desiring a cigarette	1 2 3 4 5
	When things are just not going the way I want and I am frustrated	1 2 3 4 5
	With my spouse or close friend who is smoking	1 2 3 4 5
	When there are arguments and conflicts with my family	1 2 3 4 5
	When I am happy and celebrating	1 2 3 4 5
	When I am very angry about something or someone	1 2 3 4 5
	When I would experience an emotional crisis, such as an accident or death in the family	1 2 3 4 5
	When I see someone smoking and enjoying it	1 2 3 4 5
	When I realize that quitting smoking is an extremely difficult task for me	1 2 3 4 5
	When I am craving a cigarette	1 2 3 4 5
	When I first get up in the morning	1 2 3 4 5
	When I need to lift my spirits	1 2 3 4 5
	With friends at a party	1 2 3 4 5
	When I am extremely anxious or depressed	1 2 3 4 5
	When I am stressed	1 2 3 4 5

	When I realize I haven't smoked for a while	1 2 3 4 5
21.	**At this time, what is your personal goal with regards to smoking?**<br>1 = To quit and stay off forever<br>2 = To not smoke for a limited time<br>3 = To be able to control how much I smoke<br>4 = To quit someday but not now<br>5 = To continue to smoke	1 2 3 4 5 6

Alcohol Use

1.	How often did you have a drink containing alcohol, even beer or wine?<br><br>**If Never, skip to next section "Other substance use"**	(0) Never<br>(1) Monthly or less<br>(2) 2-4 times per month<br>(3) 2-3 times per week<br>(4) ≥ 4 times per week
2.	How many drinks do you have on a typical day when you drink?<br><br>**If response is 5-6, 7-9 or 10 or more, continue to Q3. If not, continue to section on other substance use.**	(0) 1-2<br>(1) 3-4<br>(2) 5-6<br>(3) 7-9<br>(4) 10 or more
3.	How often do you have six or more drinks on one occasion?	(0) Never<br>(1) Less than monthly<br>(2) Monthly<br>(3) Weekly<br>(4) Daily or almost daily
Low Risk Limits:<br>Men: 4 per occasion; 14 per week<br>Women/ People over 65: 3 per occasion; 7 per week		

Other substance Use

1	Have you ever used marijuana (include colloquial name)?<br>**If No, skip to Q7**	☐ Yes<br>☐ No
2	Have you used marijuana in the past 3 months?<br>**If No, skip to Q7.**	☐ Yes<br>☐ No
3	When last did you use marijuana?	☐ Within the past few days<br>☐ A week ago<br>☐ Two to four weeks ago<br>☐ More than four weeks ago
4.	How often do you use marijuana?	☐ Less than monthly<br>☐ Monthly<br>☐ Weekly<br>☐ Daily/ Almost daily
5.	Do you ever combine marijuana with tobacco?<br>**If No, skip to section on other drugs.**	☐ Yes<br>☐ No
6.	How often do you combine marijuana with tobacco?	☐ Less than monthly<br>☐ Monthly<br>☐ Weekly<br>☐ Daily/Almost daily
**Now I will ask you questions about some other drugs.**		
7.	Have you ever used any other "hard drugs"?<br>**If No, exit questionnaire**<br><br>**If Yes, what did you use? (Select all that apply)**<br>1 = Heroin<br>2 = Cocaine<br>3 = LSD	☐ Yes<br>☐ No<br><br>☐<br>☐<br>☐

	4 = PCP<br>5 = Methamphetamine<br>6 = Ecstasy	☐<br>☐<br>☐
8.	Have you used Drug X in the past 3 months?<br>**If No, exit questionnaire**	☐ Yes<br>☐ No
9.	When last did you use drug X?	☐ Within the past few days<br>☐ A week ago<br>☐ Two to four weeks ago<br>☐ More than four weeks ago
10.	How often do you use drug X?	☐ Less than monthly<br>☐ Monthly<br>☐ Weekly<br>☐ Daily/ Almost Daily

Appendix B4: Follow-Up Smoking Questionnaire

BSMART

Follow-up Smoking Questionnaire

**Researcher ID:**	**Participant ID:**
**District:**	**Facility:**
**Date: (dd/mon/yyyy)**	

**Please answer these questions based on your experiences with smoking since the last time we saw you. When you select an answer, I will enter it on the form in the tablet.**		
	**Question**	**Response**
We are going to ask you some questions about your smoking behavior		
1.	Have you smoked a cigarette, even a puff, during the past 30 days?	☐ Yes<br>☐ No
2.	Have you smoked a cigarette, even a puff, during the past 7 days?	☐ Yes<br>☐ No
3.	Are you currently smoking regularly?<br><br>**If yes, how many cigarettes a day?**	☐ Yes<br>☐ No<br>_________ cigarettes
4.	How many days of the week do you smoke?	☐ 7<br>☐ 6<br>☐ 5<br>☐ 4<br>☐ 3<br>☐ 2<br>☐ 1
Now we are going to ask you some questions about quitting smoking		

5.	Since your last study visit, have you ever quit for a period of at least 24 hours?<br>**If No, skip to Q9.**	☐ Yes<br>☐ No
6.	Since your last study visit, how many times have you quit for at least 24 hours? (If more than 9 times, put 9)	________ times
7.	After your most recent quit attempt, how long did you stay off cigarettes?	☐ 1-2 days<br>☐ A week<br>☐ 2-4 weeks<br>☐ Over 4 weeks
8.	While trying to quit, how serious have each of the following problems been for you?<br>**(Use key below)**<br>**1 = Not at all**<br>**2 = A little**<br>**3 = Moderately**<br>**4 = Very**<br>**5 = Extremely**	
	▪ Weight gain	1 2 3 4 5
	▪ Increased eating	1 2 3 4 5
	▪ Digestive problems	1 2 3 4 5
	▪ Nausea	1 2 3 4 5
	▪ Headaches	1 2 3 4 5
	▪ Drowsiness	1 2 3 4 5
	▪ Depression or low mood	1 2 3 4 5
	▪ Fatigue	1 2 3 4 5
	▪ Insomnia	1 2 3 4 5
	▪ Difficulty concentrating	1 2 3 4 5
	▪ Heart pounding, or sweating	1 2 3 4 5
	▪ Irritability	1 2 3 4 5
	▪ Restlessness	1 2 3 4 5
	▪ Anxiety	1 2 3 4 5

	▪ Craving for tobacco	1 2 3 4 5
Now we will ask you some questions about your personal goals concerning smoking.		
9.	At this time, what is your personal goal with regards to smoking?<br>**1 = To stay off forever (I have already quit)**<br>**2 = To quit and stay off forever**<br>**3 = To not smoke for a limited time**<br>**4 = To be able to control how much I smoke**<br>**5 = To quit someday but not now**<br>**6 = To continue to smoke**<br><br>If response to Q9 is ***"to stay off forever (I have already quit)"***, skip to Q10.<br>If response to Q9 is ***"To quit and stay off forever"***, continue to Q11.<br>If Response to Q9 is to quit someday but not now or to continue to smoke, skip to Q12.	1 2 3 4 5 6
10.	How confident are you that you will be able to stay off forever at this time?<br>**1 = Not at all confident**<br>**2 = Somewhat confident**<br>**3 = Moderately confident**<br>**4 = Very confident**<br>**5 = Extremely confident**	1 2 3 4 5
11.	How confident are you that you will be able to stop at this time?<br>**1 = Not at all confident**<br>**2 = Somewhat confident**<br>**3 = Moderately confident**<br>**4 = Very confident**<br>**5 = Extremely confident**	1 2 3 4 5

12.	What is the most important factor that has stopped you from making a quit attempt?	

Smoking situations: Confidence

Listed below are situations that lead some people to smoke. We should like to know how confident you are that you would not smoke in these situations.

Please answer the following question by using a 5-point scale with **5 = extremely confident; 4 = very confident; 3 = moderately confident; 2 = not very confident and 1 = not at all confident**

13. How confident are you that you would NOT smoke in this situation?

	At a bar or cocktail lounge having a drink	1 2 3 4 5
	When I am desiring a cigarette	1 2 3 4 5
	When things are just not going the way I want and I am frustrated	1 2 3 4 5
	With my spouse or close friend who is smoking	1 2 3 4 5
	When there are arguments and conflicts with my family	1 2 3 4 5
	When I am happy and celebrating	1 2 3 4 5
	When I am very angry about something or someone	1 2 3 4 5
	When I would experience an emotional crisis, such as an accident or death in the family	1 2 3 4 5
	When I see someone smoking and enjoying it	1 2 3 4 5
	When I realize that quitting smoking is an extremely difficult task for me	1 2 3 4 5
	When I am craving a cigarette	1 2 3 4 5
	When I first get up in the morning	1 2 3 4 5
	When I need to lift my spirits	1 2 3 4 5
	With friends at a party	1 2 3 4 5
	When I am extremely anxious or depressed	1 2 3 4 5
	When I am stressed	1 2 3 4 5
	When I realize I haven't smoked for a while	1 2 3 4 5

Appendix B5: Stages of Change Algorithm

UMBC

myUMBC | Events | Directory

Enter Search Terms

The HABITS Lab at UMBC

Health and Addictive Behaviors: Investigating Transtheoretical Solutions

HOME | ABOUT THE LAB ▼ | THE MODEL ▼ | PUBLICATIONS | PRESENTATIONS | RELATED LINKS | CONTACT US

Staging Algorithms

Smoking Algorithm

CLASSIFICATION OF THE STAGES OF CHANGE FOR SMOKING CESSATION

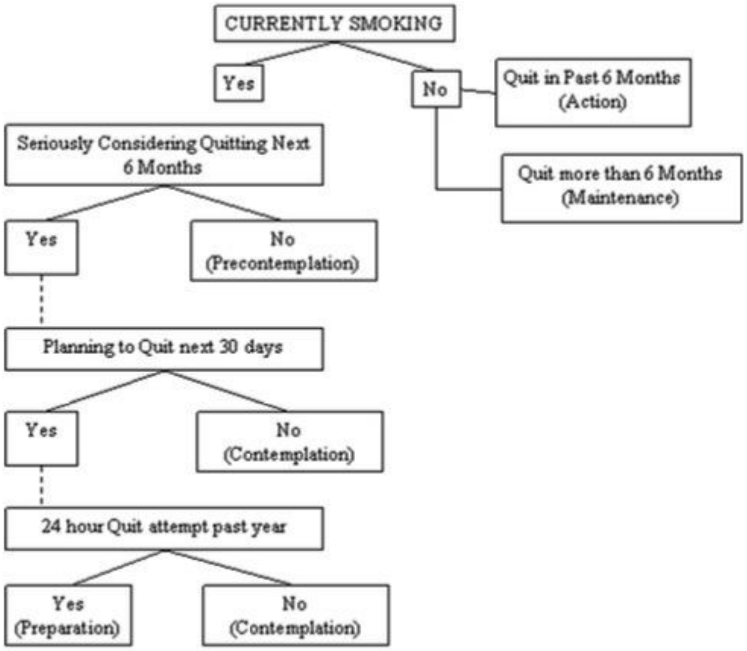

Smoking Stage of Change
Client ID#______________________

Short Form   Date:_________/_________/_______

Assessment Point:_________________

1. Are you currently a smoker?

- A) Yes, I currently smoke.
- B) No, I quit within the last 6 months.
- C) No, I quit more than 6 months ago.
- D) No, I have never smoked.

Smokers only:

2. In the last year, how many times have you quit smoking for at least 24 hours? _____

3. Are you seriously thinking of quitting smoking?

- A) Yes, within the next 30 days
- B) Yes, within the next 6 months
- C) No, not thinking of quitting

Smoking Stage of Change
Short Form
Scoring Sheet

1. Are you currently a smoker?

- A) Yes, I currently smoke.
- B) No, I quit within the last 6 months. (ACTION STAGE)
- C) No, I quit more than 6 months ago. (MAINTENANCE STAGE)
- D) No, I have never smoked. (NONSMOKER)

Smokers only:

2. In the last year, how many times have you quit smoking for at least 24 hours? ______

3. Are you seriously thinking of quitting smoking?

- A) Yes, within the next 30 days (PREPARATION STAGE if they have one 24-hour quit attempt in the past year; if there was no quit attempt in the past year, then CONTEMPLATION STAGE)
- B) Yes, within the next 6 months (CONTEMPLATION)
- C) No, not thinking of quitting (PRECONTEMPLATION)

Appendix B6: Readiness Ruler

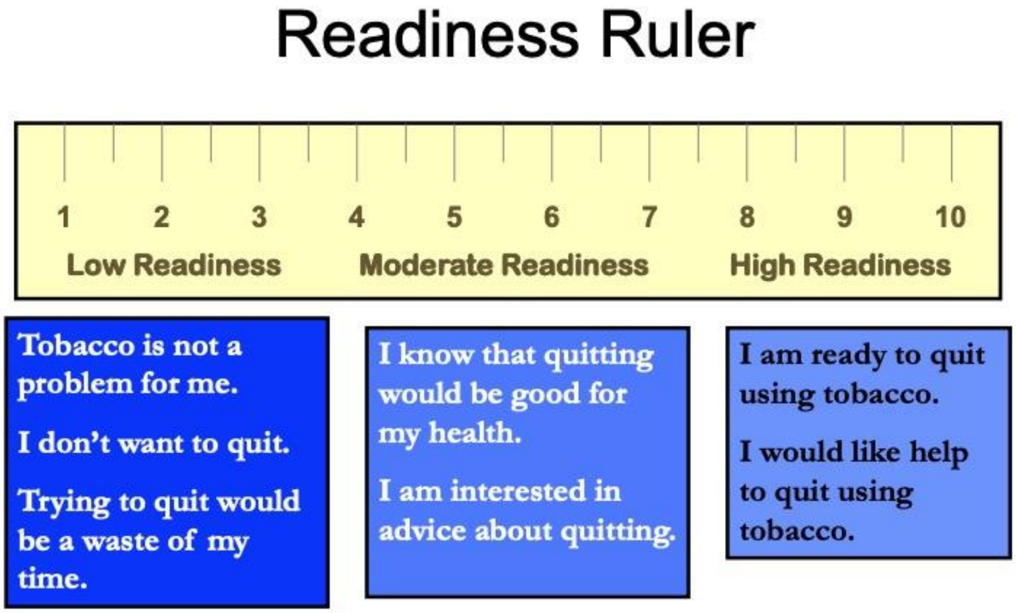

MARYLAND RESOURCE CENTER
FOR QUITTING USE & INITIATION OF TOBACCO

Maryland's 1-800 QUIT NOW
SmokingStopsHere.com

Appendix C: Tools for Varenicline Use

Appendix C1: Varenicline Indications and Uses

INDICATIONS AND USAGE

CHANTIX is a nicotinic receptor partial agonist indicated for use as an aid to smoking cessation treatment. (1 and 2.1)

DOSAGE AND ADMINISTRATION

Begin CHANTIX dosing one week before the date set by the patient to stop smoking. Alternatively, the patient can begin CHANTIX dosing and then quit smoking between days 8 and 35 of treatment. (2.1)

Starting Week: 0.5 mg once daily on days 1–3 and 0.5 mg twice daily on days 4–7. (2.1)

Continuing Weeks: 1 mg twice daily for a total of 12 weeks. (2.1)

An additional 12 weeks of treatment is recommended for successful quitters to increase likelihood of long-term abstinence. (2.1)

Consider a gradual approach to quitting smoking with CHANTIX for patients who are sure that they are not able or willing to quit abruptly. Patients should begin CHANTIX dosing and reduce smoking by 50% from baseline within the first four weeks, by an additional 50% in the next four weeks, and continue reducing with the goal of reaching complete abstinence by 12 weeks. Continue treatment for an additional 12 weeks, for a total of 24 weeks. (2.1)

Severe Renal Impairment (estimated creatinine clearance less than 30 mL/min): Begin with 0.5 mg once daily and titrate to 0.5 mg twice daily. For patients with end-stage renal disease undergoing hemodialysis, a maximum of 0.5 mg daily may be given if tolerated. (2.2)

Consider dose reduction for patients who cannot tolerate adverse effects. (2.1)

Another attempt at treatment is recommended for those who fail to stop smoking or relapse when factors contributing to the failed attempt have been addressed. (2.1)

Provide patients with appropriate educational materials and counseling to support the quit attempt. (2.1)

DOSAGE FORMS AND STRENGTHS

Tablets: 0.5 mg and 1 mg (3)

CONTRAINDICATIONS

History of serious hypersensitivity or skin reactions to CHANTIX. (4)

WARNINGS AND PRECAUTIONS

Neuropsychiatric Adverse Events: Postmarketing reports of serious or clinically significant neuropsychiatric adverse events have included changes in mood (including depression and mania), psychosis, hallucinations, paranoia, delusions, homicidal ideation, aggression, hostility, agitation, anxiety, and panic, as well as suicidal ideation, suicide attempt, and completed suicide. Observe patients attempting to quit smoking with CHANTIX for the occurrence of such symptoms and instruct them to discontinue CHANTIX and contact a healthcare provider if they experience such adverse events. (5.1)

Seizures: New or worsening seizures have been observed in patients taking CHANTIX. CHANTIX should be used cautiously in patients with a history of seizures or other factors that can lower the seizure threshold. (5.2)

Interaction with Alcohol: Increased effects of alcohol have been reported. Instruct patients to reduce the amount of alcohol they consume until they know whether CHANTIX affects them. (5.3)

Accidental Injury: Accidental injuries (e.g., traffic accidents) have been reported. Instruct patients to use caution driving or operating machinery until they know how CHANTIX may affect them. (5.4)

Cardiovascular Events: Patients with underlying cardiovascular (CV) disease may be at increased risk of CV events; however, these concerns must be balanced with the health benefits of smoking cessation. Instruct patients to notify their healthcare providers of new or worsening CV symptoms and to seek immediate medical attention if they experience signs and symptoms of myocardial infarction (MI) or stroke. (5.5 and 6.1)

Somnambulism: Cases of somnambulism have been reported in patients taking CHANTIX. Some cases described harmful behavior to self, others, or property. Instruct patients to discontinue CHANTIX and notify their healthcare provider if they experience somnambulism. (5.6 and 6.2)

Angioedema and Hypersensitivity Reactions: Such reactions, including angioedema, infrequently life-threatening, have been reported. Instruct patients to discontinue CHANTIX and immediately seek medical care if symptoms occur. (5.7 and 6.2)

Serious Skin Reactions: Rare, potentially life-threatening skin reactions have been reported. Instruct patients to discontinue CHANTIX and contact a healthcare provider immediately at first appearance of skin rash with mucosal lesions. (5.8 and 6.2)

Nausea: Nausea is the most common adverse reaction (up to 30% incidence rate). Dose reduction may be helpful. (5.9)

ADVERSE REACTIONS

Most common adverse reactions (>5% and twice the rate seen in placebo-treated patients) were nausea, abnormal (e.g., vivid, unusual, or strange) dreams, constipation, flatulence, and vomiting. (6)

To report SUSPECTED ADVERSE REACTIONS, contact Pfizer Inc. at 1-800-438-1985 or FDA at 1-800-FDA-1088 or www.fda.gov/medwatch.

DRUG INTERACTIONS

Other Smoking Cessation Therapies: Safety and efficacy in combination with other smoking cessation therapies has not been established. Coadministration of varenicline and transdermal nicotine resulted in a high rate of discontinuation due to adverse events. (7.1)

Effect of Smoking Cessation on Other Drugs: Pharmacokinetics or pharmacodynamics of certain drugs (e.g., theophylline, warfarin, insulin) may be altered, necessitating dose adjustment. (7.2)

Appendix C2: Common Terminology Criteria for adverse Events and Common Toxicity Criteria

(See accompanying PDF)

Appendix C3: Exclusion Criteria for Varenicline Use

Criteria	Mode of Detection
Previous Allergic Reaction or hypersensitivity to Varenicline	Participant report ever in lifetime
Pregnant, nursing, or becoming pregnant during the study	Pregnancy Test
Current use of any medication that could interfere with the protocol (such as Bupropion)	Patient history and chart review
Moderate to severe renal impairment	Patient history and chart review
Unstable cardiovascular disease (myocardial infarction in the past year, uncontrolled arrythmia, uncontrolled congestive heart failure	Clinician assessment and chart review
The study clinician feels the individual is not medically stable enough to participate in the study	Past medical history and/ or current medical status

Appendix C4: BSMART Medication Adherence Form

BSMART Medication Adherence Form

**Researcher ID:**	**Participant ID:**
**District:**	**Facility:**
**Date: (dd/mm/yyyy)**	

(Completed only at Week 1, 2, 3, 4, 8, and 12)

**Week:**					
☐ **Week 1**	☐ **Week 2**	☐ **Week 3**	☐ **Week 4**	☐ **Week 8**	☐ **Week 12**

How many days last week did you take at least 1 of your study pills?

____

(Completed only at week 2, 3, 4, 8, and 12)

**Week:**				
☐ **Week 2**	☐ **Week 3**	☐ **Week 4**	☐ **Week 8**	☐ **Week 12**

How many days last week did you take at least 2 of your study pills?

____

Appendix C5: BSMART Side-effects Checklist

BSMART Side Effect Checklist

**Researcher ID:**	**Participant ID:**
**District:**	**Facility:**
**Date: (dd/mon/yyyy)**	

**Week Number:**					
☐ **Week 1**	☐ **Week 2**	☐ **Week 3**	☐ **Week 4**	☐ **Week 8**	☐ **Week 12**

Ask the participant if he/she experienced these side effects in the last week. If participant endorses a side effect, then ask if it was mild, moderate or severe. If participant endorses a side effect, also ask how often it happened and if they took any medications for it or called a doctor. Make any notes at the back of this form. Use the following format: "Did you experience nausea in the last week?" If yes, continue with: "Would you say the nausea was mild, moderate or severe? How often did you experience nausea in the last week? Did you take any medications or call a doctor for your nausea in the last week?"

**Side Effect**	**None (0)**	**Mild (1)**	**Moderate (2)**	**Severe (3)**	**Not Rated (4)**	**Missing (99)**	**Related to Medication: A = None; B = Remote; C = Possible; D = Probable; E = Missing**
1. Nausea	☐	☐	☐	☐	☐	☐	☐ A ☐ B ☐ C ☐ D ☐ E
2. Vomiting	☐	☐	☐	☐	☐	☐	☐ A ☐ B ☐ C ☐ D ☐ E
3. Constipation	☐	☐	☐	☐	☐	☐	☐ A ☐ B ☐ C ☐ D ☐ E
4. Dizziness	☐	☐	☐	☐	☐	☐	☐ A ☐ B ☐ C ☐ D ☐ E
5. Disturbance in attention	☐	☐	☐	☐	☐	☐	☐ A ☐ B ☐ C ☐ D ☐ E
6. Anorexia (loss of appetite)	☐	☐	☐	☐	☐	☐	☐ A ☐ B ☐ C ☐ D ☐ E
7. Increased appetite	☐	☐	☐	☐	☐	☐	☐ A ☐ B ☐ C ☐ D ☐ E
8. Weight loss	☐	☐	☐	☐	☐	☐	☐ A ☐ B ☐ C ☐ D ☐ E

9. Weight increase	☐	☐	☐	☐	☐	☐	☐ A ☐ B ☐ C ☐ D ☐ E
10. Gastroesophageal reflux	☐	☐	☐	☐	☐	☐	☐ A ☐ B ☐ C ☐ D ☐ E
11. Dyspepsia (indigestion)	☐	☐	☐	☐	☐	☐	☐ A ☐ B ☐ C ☐ D ☐ E
12. Insomnia	☐	☐	☐	☐	☐	☐	☐ A ☐ B ☐ C ☐ D ☐ E
13. Abnormal dreams	☐	☐	☐	☐	☐	☐	☐ A ☐ B ☐ C ☐ D ☐ E
14. Sleep disorder	☐	☐	☐	☐	☐	☐	☐ A ☐ B ☐ C ☐ D ☐ E
15. Nightmares	☐	☐	☐	☐	☐	☐	☐ A ☐ B ☐ C ☐ D ☐ E
16. Abdominal pain	☐	☐	☐	☐	☐	☐	☐ A ☐ B ☐ C ☐ D ☐ E
17. Chest pain	☐	☐	☐	☐	☐	☐	☐ A ☐ B ☐ C ☐ D ☐ E
18. Back pain	☐	☐	☐	☐	☐	☐	☐ A ☐ B ☐ C ☐ D ☐ E
19. Myalgia (muscle pain)	☐	☐	☐	☐	☐	☐	☐ A ☐ B ☐ C ☐ D ☐ E
20. Arthralgia (joint pain)	☐	☐	☐	☐	☐	☐	☐ A ☐ B ☐ C ☐ D ☐ E
21. Stiffness	☐	☐	☐	☐	☐	☐	☐ A ☐ B ☐ C ☐ D ☐ E
22. Tremor	☐	☐	☐	☐	☐	☐	☐ A ☐ B ☐ C ☐ D ☐ E
23. Restlessness	☐	☐	☐	☐	☐	☐	☐ A ☐ B ☐ C ☐ D ☐ E
24. Headache	☐	☐	☐	☐	☐	☐	☐ A ☐ B ☐ C ☐ D ☐ E
25. Fever	☐	☐	☐	☐	☐	☐	☐ A ☐ B ☐ C ☐ D ☐ E
26. Sore throat	☐	☐	☐	☐	☐	☐	☐ A ☐ B ☐ C ☐ D ☐ E
27. Dry mouth	☐	☐	☐	☐	☐	☐	☐ A ☐ B ☐ C ☐ D ☐ E
28. Hypersalivation	☐	☐	☐	☐	☐	☐	☐ A ☐ B ☐ C ☐ D ☐ E
29. Mucousal ulceration	☐	☐	☐	☐	☐	☐	☐ A ☐ B ☐ C ☐ D ☐ E
30. Toothache	☐	☐	☐	☐	☐	☐	☐ A ☐ B ☐ C ☐ D ☐ E
31. Dysgeusia (metallic taste)	☐	☐	☐	☐	☐	☐	☐ A ☐ B ☐ C ☐ D ☐ E
32. Respiratory Disorder	☐	☐	☐	☐	☐	☐	☐ A ☐ B ☐ C ☐ D ☐ E
33. Dyspnea (labored breathing)	☐	☐	☐	☐	☐	☐	☐ A ☐ B ☐ C ☐ D ☐ E
34. Rhinorrhea (nasal mucous)	☐	☐	☐	☐	☐	☐	☐ A ☐ B ☐ C ☐ D ☐ E
35. Malaise (fatigue/asthenia/lethargy)	☐	☐	☐	☐	☐	☐	☐ A ☐ B ☐ C ☐ D ☐ E
36. Epidermal/dermal pruritus (rash)	☐	☐	☐	☐	☐	☐	☐ A ☐ B ☐ C ☐ D ☐ E
37. Uticaria (hives, itching)	☐	☐	☐	☐	☐	☐	☐ A ☐ B ☐ C ☐ D ☐ E

38. Bruising easily	☐	☐	☐	☐	☐	☐	☐ A ☐ B ☐ C ☐ D ☐ E
39. Tinnitus (ringing in the ears)	☐	☐	☐	☐	☐	☐	☐ A ☐ B ☐ C ☐ D ☐ E
40. Liver abnormalities	☐	☐	☐	☐	☐	☐	☐ A ☐ B ☐ C ☐ D ☐ E
41. Other ___________________	☐	☐	☐	☐	☐	☐	☐ A ☐ B ☐ C ☐ D ☐ E
42. Other ___________________	☐	☐	☐	☐	☐	☐	☐ A ☐ B ☐ C ☐ D ☐ E
43. Other ___________________	☐	☐	☐	☐	☐	☐	☐ A ☐ B ☐ C ☐ D ☐ E
44. Other ___________________	☐	☐	☐	☐	☐	☐	☐ A ☐ B ☐ C ☐ D ☐ E
45. Additional Comments:							

Appendix C6: BSMART Contraception Consent Form

BSMART Contraception Consent

**Researcher ID:**	**Participant ID:**
**District:**	**Facility:**
**Date: (dd/mm/yyyy)**	

Do you agree to use an acceptable method of contraception from the time of screening until all medication is completed (approximately 12 weeks)?

Acceptable methods of contraception include intra-uterine device, condom, spermicidal gels, diaphragm, etc. (please initial one of the following)

________ Yes ________ No

*Condoms will be available (for free) at study sites

*If male, please initial here to waive contraception consent form: __________

_________________________ _______________

Participant Date

_________________________ _______________

Staff Signature Date

Appendix C7: BSMART Prescription Form

Protocol: Botswana Smoking Abstinence Reinforcement Trial

IRB Protocol Number:

Principal Investigator: Manhattan Charurat, PhD, MSc

Subject Name ______________________________________

Study Number______________________________________

Date of Birth_______________________________________

Protocol Prescription

Days 1-3: Dates_____________________________________

Varenicline (0.5mg, total 0.5mg daily): take one tablet (0.5mg) daily, one tablet in the morning after eating and with a full glass (8 ounces) of water.

Days 4 - 7: Dates_____________________________________

Varenicline (0.5mg, total 1mg daily): take one tablet (0.5mg) twice daily, one tablet in the morning and one tablet in the evening, after eating and with a full glass (8ounces) of water.

Weeks 2 - 12: Dates_____________________________________

Varenicline (0.5mg, total 2mg daily): take two tablets (1mg) twice daily, two tablets in the morning and two tablets in the evening, after eating and with a full glass (8ounces) of water.

Prescribers Name: _______________________________________

Prescribers Signature: ____________________________________

Date: ____

Appendix C8: BSMART quit day preparation

QUIT Day Preparation

When is it?

Quit day for everyone taking Apotex is

on the 8th day of taking the medicine.

This means that your quit day will be ________________________________.

What to do on Quit Day

Getting ready for your quit day starts many days before the Quit Day. Remember, your "Quit Day" starts on the morning of your 8th day on Apotex. You are to QUIT smoking as soon as you wake up that day. In order to quit on the morning of "Quit day", you must prepare your mind and your body the night before.

Tips to prepare for Quit Day:

1. **Make sure that you have PERMANENTLY gotten rid of any remaining cigarettes before going to bed. This means any hidden cigarettes, current packs or unopened packs should be disposed of.**
2. **You should keep in close contacts with friends or loved ones during this week if possible so that you can receive support when you need it.**

3. **It is very helpful to have a plan, perhaps hour-by-hour for not smoking for the first day or even the first few days after quitting.**
4. **As part of preparation, you should go over your reasons for quitting smoking.**

Appendix D: Focus Group Guides

Appendix D1: FGD Guide for Lay Health Workers

BSMART

Focus Group Introduction Script for Lay Health Workers

Reminder, you don't need to follow this language exactly. It's important that you…

- **Introduce yourself and any other members of the research team present**
- **Remind participants of the purpose of the FGD**
- **What to expect and norms**
  - **You may ask people to expand**
  - **You may ask the group to move on**
  - **People may not agree or have different experiences – that's okay**
  - **It's okay for participants to respond to one another or ask each other questions**
- **Remind participants that the FGD will be recorded**
- **Underscore the importance of keeping the conversation private**
- **Ask if anyone has any questions**

Welcome and thank you all for being here today. My name is _________ and I will be moderating the focus group discussion today. My role is to facilitate discussion by prompting you all with questions and making sure everyone gets a chance to share. I'd also like to introduce my colleague __________ who will be taking notes to ensure we accurately capture what you share with us today.

As a reminder, the purpose of our discussion today is to hear about your experiences integrating the SBIRT intervention into your work and what it has been like for you to implement this intervention. The implementation and evaluation of SBIRT is part of a collaborative effort between the University of Maryland, Baltimore, University of Botswana, and the Botswana University of Maryland Medicine Health Initiative (BUMMHI). The feedback you share with us today will be used to help us better understand how implementation has been going and how we may improve it.

Our goal today is to have a discussion about your experiences with the SBIRT intervention – screening people for participating, implementing the brief intervention, and referring to other resources. Everyone here today has been trained to screen and oversee the SBIRT intervention procedures and has been implementing these procedures for about three months.

I will be asking you all to respond to a series of prompts about the different aspects of the intervention. There are no right, or wrong answers and it is okay if you disagree with one another. I'm interested in hearing everyone's experiences and not a group consensus. So, please feel to respond to one another and even ask each other questions, you do not need to wait for me to do so. My role is simply to make sure our discussion stays on topic and covers all aspects of the intervention. Everyone's perspective is important, so I encourage you all to participate in the discussion.

There may be times when I do ask someone to explain their comment further and other times when I ask that we move onto the next topic. I will do these things to ensure that we fully understand what everyone has to say while also making sure we get through all the content we need to cover and respect everyone's time. Our discussion should take about an hour.

As a reminder, once we get started our discussion will be audio recorded. The purpose of the recording is to help us accurately capture your experiences. To make sure we capture everyone well, please be sure to speak up so the microphone(s) [**gesture to where the microphone(s) are in the room**] pick you up. Also, please try to not talk over each other so that we can clearly document what everyone has to say.

It is very important that we agree that everything we discuss in this room today is private. As was explained in the consent form you signed to participate, my colleagues and I are not going to use anyone's name or other identifying information. We will keep everything private, and we expect all of you to also respect each other's privacy by not sharing what was discussed here with others.

Now, before we get started, does anyone have any questions?

[after responding to any questions or concerns] **START RECORDING**

Semi-Structured Focus Group Discussion Guide

Reminder: this is a semi-structured guide. You should be responsive to the discussion as it unfolds which may require that you ask the prompts in a different order in order to maintain conversation flow better.

There may also be instances when a prompt has already been sufficiently answered in the conversation. When this happens, acknowledge that participants have talked about this topic already and ask if anyone has anything to add to it before moving on.

Your primary responsibilities are to

- **Make sure all content areas are discussed**
- **Make sure all participants are given the chance to speak when they have something to say**
- **Manage time**

Be sure to check-in with other team members prior to the participants arriving so that you can discuss how everyone can perform their roles and support one another during the FGD

Alright, let's go ahead and get started.

SECTION A: SBIRT TRAINING

First, I'd like to just get an idea of how well prepared you felt to implement the SBIRT screening tool and generally providing SBIRT to your [patients/clients/participants]. So, if you think back to the training, you received…

1. **How effective was the training you received on SBIRT in preparing you for actually implementing it?**

   Prompts (if needed):

   - *Was the content comprehensive?*
   - *Did you feel confident about implementing the SBIRT when you started?*
   - *What was the most useful part of the training?*

2. What changes to the training would improve it to make it more useful?

Prompts (if needed):

- *Should any content be added or expanded? If so, what?*
- *Should any content be removed? If so, what?*

3. Do you have any other comments about your experiences with the SBIRT training?

SECTION B: SBIRT IMPLEMENTATION

Great, thank you everyone for that feedback. Now I'd like to hear about your experiences implementing SBIRT and I want to begin with the SBIRT screening tool.

1. **Generally, what have your experiences with using the SBIRT screening tool (i.e., questionnaire) been like?**

   Prompts (if needed):

   - *Has it been easy to use?*
   - *Have you experienced any difficulties or challenges with it?*

2. **How did [clients/patients/participants] respond to being screened?**

   Prompts (if needed):

   - *Were they positive, negative, or neutral about it?*

3. **Do you have any recommendations for improving the screening tool?**

   Prompts (if needed):

   - *Do any aspects need to be changed? Added? Removed?*
   - *Do you have all the support you need for using it?*

Now I'd like to hear more broadly about your experiences providing the brief SBIRT intervention.

4. **Have you encountered any challenges with providing the brief SBIRT intervention?**

   Prompts (if needed):

   - *What have they been?*
   - *Why do you think that is?*

5. **Do you have any recommendations for addressing these challenges?**

   Prompts (if needed):

   - *It's okay if you have ideas that do not relate to your own challenges*

6. **Now, what is working? What works well about the brief SBIRT intervention?**

   Prompts (if needed):

   - *Why do you think that is?*

I also want to ask you about your experiences referring your [clients/patients/participants] to additional resources, like the Nurse Prescribers for receiving medications.

7. **Have you encountered any challenges with, as part of the SBIRT intervention, referring people to additional resources?**

   Prompts (if needed):

   - *What have they been?*
   - *Why do you think that is?*

8. **Do you have any recommendations for addressing these challenges?**

9. **What works well with referring people to these additional resources, like the Nurse Prescribers?**

10. **How has it been working with other people who are part of the SBIRT intervention?**

    Prompts (if needed):

    - *Nurse Prescribers?*

Thank you for sharing about your experiences, you all have provided really useful insights so far. This is the final set of questions I have for you all and they relate to your experiences working with [outpatients] as part of this intervention.

11. **How has it been recruiting outpatients to participate in SBIRT?**

    Prompts (if needed):

    - *Have they been resistant or interested in the program?*

12. **What seems to impact eligible outpatients' willingness to participate in SBIRT?**

    Prompts (if needed):

    - *Interest in quitting smoking?*
    - *Concerns about time, the medication, or aspects related to the facility?*

13. **Do you have any recommendations for how we can facilitate interest in SBIRT participation among your outpatients?**

14. **Once enrolled, have you noticed the needs of participants changing as you continue to work with them around smoking cessation?**

15. **How do you think we could address participants' shifting needs?**

SECTION C: CLOSING

Thank you everyone! That is the end of my prepared questions.

1. **Is there something else you think I should have asked you all about?**

2. **Does anyone have anything else to share about implementing SBIRT?**

Again, thank you for participating in the discussion. You all provided helpful insights about the implementation of SBIRT.

As a reminder, please respect the privacy of your fellow focus group members by not sharing what was said here with other people. If you have any questions, please feel free to ask me or my colleagues here. Your consent forms also have contact information, if anything else comes up.

STOP RECORDING

BSMART

Focus Group Participant Information Collection Document

Date: ___________________________

FGD Group: __________________________

Moderator Initials: ________________

Note taker initials: ___________________

Start Time: ________________________

Stop Time: ___________________________

Number of participants at the start of FGD: ___________

Number of participants at the end of FGD: ____________

Demographic information to be obtained for every FGD participant on a one-to-one basis, immediately after consent is obtained

Participant number or fake name	Age in complete years	Participant gender	Level of education
1.			
2.			
3.			
4.			
5.			
6.			
7.			
8.			
9.			
10.			

Appendix D2: Focus Group Guide for Nurse Prescribers

BSMART

Focus Group Introduction Script for Nurse Prescribers

Reminder, you don't need to follow this language exactly. It's important that you…

- **Introduce yourself and any other members of the research team present**
- **Remind participants of the purpose of the FGD**
- **What to expect and norms**
    - **You may ask people to expand**
    - **You may ask the group to move on**
    - **People may not agree or have different experiences – that's okay**
    - **It's okay for participants to respond to one another or ask each other questions**
- **Remind participants that the FGD will be recorded**
- **Underscore the importance of keeping the conversation private**
- **Ask if anyone has any questions**

Welcome and thank you all for being here today. My name is _________ and I will be moderating the focus group discussion today. My role is to facilitate discussion by prompting you all with questions and making sure everyone gets a chance to share. I'd also like to introduce my colleague __________ who will be taking notes to ensure we accurately capture what you share with us.

As a reminder, the purpose of our discussion today is to hear about your experiences integrating the SBIRT intervention into your work and what it has been like for you to implement this intervention. The implementation and evaluation of SBIRT is part of a collaborative effort between the University of Maryland, Baltimore, University of Botswana, and the Botswana University of Maryland Medicine Health Initiative (BUMMHI). The feedback you share with us today will be used to help us better understand how implementation has been going and how we may improve it.

Our goal today is to have a discussion about your experiences with the SBIRT intervention, specifically dispensing varenicline and monitoring its effects on participants of the intervention. Everyone here

today is a [**nurse prescriber**?] involved with this project. I will be asking you all to respond to a series of prompts about your experiences in this role.

There are no right, or wrong answers and it is okay if you disagree with one another. I'm interested in hearing everyone's experiences and not a group consensus. So, please feel to respond to one another and even ask each other questions, you do not need to wait for me to do so. My role is simply to make sure we cover all topics and manage the time we have together. Everyone's perspective is important, so I encourage you all to participate in the discussion.

There may be times when I ask someone to explain their comment further and other times when I ask that we move onto the next topic. I will do these things to ensure that we fully understand what everyone has to say while also making sure we get through all the content we need to cover and respect everyone's time. Our discussion should take about 30 minutes.

As a reminder, once we get started our discussion will be audio recorded. The purpose of the recording is to help us accurately capture your experiences. To make sure we capture everyone well, please be sure to speak up so the microphone(s) [**gesture to where the microphone(s) are in the room**] pick you up. Also, please try to not talk over each other so that we can clearly document what everyone has to say.

It is very important that we agree that everything we discuss in this room today is private. As was explained in the consent form you signed to participate, my colleagues and I are not going to use anyone's name or other identifying information. We will keep everything private, and we expect all of you to also respect each other's privacy by not sharing what was discussed here with others.

Now, before we get started, does anyone have any questions?

[after responding to any questions or concerns] **START RECORDING**

Semi-Structured Focus Group Discussion Guide for Nurse Prescribers

Reminder: this is a semi-structured guide. You should be responsive to the discussion as it unfolds which may require that you ask the prompts in a different order in order to maintain conversation flow better.

There may also be instances when a prompt has already been sufficiently answered in the conversation. When this happens, acknowledge that participants have talked about this topic already and ask if anyone has anything to add to it before moving on.

Your primary responsibilities are to

- **Make sure all content areas are discussed**
- **Make sure all participants are given the chance to speak when they have something to say**
- **Manage time**

Be sure to check-in with other team members prior to the participants arriving so that you can discuss how everyone can perform their roles and support one another during the FGD

--

Alright, let's go ahead and get started.

SECTION A: TRAINING

First, I'd like to just get an idea of how well prepared you felt to dispense varenicline to patients. So, if you think back to the training, you received…

4. **How effective was the training you received on varenicline in preparing you for dispensing it to intervention participants?**

   Prompts (if needed):

   - *Was the content comprehensive?*
   - *Did you feel confident about dispensing the medication when you started?*
   - *What was the most useful part of the training?*

5. **How prepared did you feel to complete the adherence tool form?**

   2b. What about the side effects form?

   Prompts (if needed):

   - *Did your comfort with these tools increase over time?*
   - *What about the training helped you feel prepared?*
   - *Do you have recommendations for improving training for these tools?*

6. **What changes to the training would improve it to make it more useful?**

   Prompts (if needed):

   - *Should any content be added or expanded? If so, what?*
   - *Should any content be removed? If so, what?*

7. **Do you have any other comments about your experiences with the training on varenicline?**

SECTION B: DISPENSING VARENICLINE

Great, thank you everyone for that feedback. Now I'd like to hear about your experiences actually dispensing the medication and monitoring patients.

16. **Generally, what have your experiences been with identifying participants who are eligible to use varenicline?**

17. **Generally, how have your experiences been with dispensing varenicline gone?**

    2a. What have your experiences been with completing the adherence tool?

    Prompts (if needed):

    - *Was the tool easy or difficult to fill out?*
    - *Did you receive support with filling out the tool?*

18. Have you experienced any difficulties or challenges?

3b. What recommendations would you have for addressing these challenges?

19. How has it been monitoring patients' responses to varenicline?

Prompts (if needed):

- *Are patients responsive and willing to share?*
- *Are patients reliably picking up refills on time?*

20. Would you say patients' reactions to varenicline have been mostly positive or mostly negative?

5a. Why do you say that?

21. Have you had to fill out the side effects tool, at least once?

6a. [if yes]. How did it go filling out the tool?

Prompts (if needed):

- *Was it difficult or easy?*
- *Did you receive any support with it?*

22. Do you have any recommendations for improving your ability to monitor patients?

23. Do you have any recommendations for improving patients' experiences with varenicline?

24. Do you have any recommendations for improving the tools you have to complete?

Prompts (if needed):

- *Adherence tool?*
- *Side effects tool?*

SECTION C: CLOSING

Thank you for sharing about your experiences, you all have provided really useful insights so far. This is the final set of questions I have for you.

1. **How has it been working with other people who are part of the SBIRT intervention?**

   Prompts (if needed):

   - *Lay healthcare workers?*
   - *Case managers?*

2. **Do you have any recommendations for improving the SBIRT intervention, in general?**

Thank you everyone! That is the end of my prepared questions.

3. **Is there something else you think I should have asked you all about?**

4. **Does anyone have anything else to share about dispensing varenicline or the SBIRT intervention?**

Again, thank you for participating in the discussion. You all provided helpful insights.

As a reminder, please respect the privacy of your fellow focus group members by not sharing what was said here with other people. If you have any questions, please feel free to ask me or my colleagues here. Your consent forms also have contact information, if anything else comes up.

STOP RECORDING

BSMART

Focus Group Participant Information Collection Document

Date: ___________________________ **FGD Group:** __________________________

Moderator Initials: _________________ **Note taker initials:** ____________________

Start Time: ________________________ **Stop Time:** ___________________________

Number of participants at the start of FGD: ___________

Number of participants at the end of FGD: ____________

Demographic information to be obtained for every FGD participant on a one-to-one basis, immediately after consent is obtained

Participant number or fake name	Age in complete years	Participant gender	Level of education
1.			
2.			
3.			
4.			
5.			
6.			
7.			
8.			
9.			
10.			

Appendix E: Semi-structured Interview Questionnaires

Appendix E1: SSI for participants who did not quit smoking

BSMART Participant Interview Guide

Participants Who Did Not Quit Smoking

> ***Note to Interviewer:***
>
> **Text in red refers to important directions for you.**

SECTION A: INTRODUCTION

Thank you for taking the time to talk with me today. My name is ___________ and I am part of a team doing research on how we can help people quit smoking, especially when they encounter challenges when trying to do so.

Our conversation today will be quite informal, so please feel free to share anything you think would be important or helpful for us to know about your experiences with smoking and trying to quit.

I have a list of prepared questions that I will ask you. Sometimes I may ask you to expand or clarify your responses, I do this to make sure I fully understand. Other times I may ask that we move onto the next topic so that I can respect your time and ensure we can cover everything we need to get through.

You may or may not remember that I will be audio recording this interview. The reason for that is so that I can focus on what you are saying without trying to write it all down. No one will hear the recording except for people working on the project and we will remove any identifying information from the transcript of the interview. In reports from this project, we will never identify you as a participant or provide any information that allows other people to figure out you took part.

As a reminder, your participation in this interview is completely voluntary. If there are any questions you'd rather not answer, that is fine – just let me know. And, if at any point, you need a break, you can let me know that too. We are interested in your honest thoughts and experiences so there are no "right" or "wrong" answers to my questions.

Do you have any questions before we begin the interview?

[after responding to any questions or concerns] **START RECORDING**

Let's get started!

SECTION B: SMOKING EXPERIENCES

First, I want to ask you a little about your history with smoking tobacco. Please, can you take a moment to think back to when you began smoking.

1. **How old were you when you started smoking regularly?**

   Prompt (if needed):

   If participant cannot give an exact age, a ballpark number or some other indicator (e.g., a year in school; before/after a significant life event) works as well

2. **What influenced you to begin smoking?**

   Prompt (if needed):

   - *What led you to start smoking?*
   - *Did you feel pressured by anyone to smoke?*

Now thinking about over the last week

3. **Tell me about when and how much you smoke.**

   Prompt (if needed):

   - *What time of day?*
   - *How many times on a typical day?*

4. **Does having HIV impact your smoking behaviors?**

   Prompt (if needed):

   - *Do you smoke more, less, or the same amount?*
   - *Why do you think that is?*

5. **What makes you smoke?**

   Prompt (if needed):

   - *Pleasure, alleviate boredom*
   - *Stress, manage depression or anxiety, manage anger*

- *Avoid withdrawal symptoms*
- *Manage pain or medications (e.g., make them work better, remove bad taste)*
- *Part of a social activity*
- *Weight management*
- *Need to (addicted to it); too hard to quit*
- *Avoid use of harder drugs or alcohol*
- *Helps with energy levels or concentration*
- *Not worried about health*

6. **Are these things that make you smoke now different from the things that made you smoke when you first started?**

   Prompt (if needed):

   - *Why do you think that is?*

Now, when you think about these reasons for why you smoke *[note a few or all of them]*

7. **Which of these reasons for smoking make it most difficult to quit?**

SECTION C: SMOKING CESSATION

Thank you for everything you have shared so far. I want to ask you a bit more about your thoughts and experiences with quitting smoking.

1. **Have you recently tried to quit smoking?** *IF YES,*

   a. **What reasons, recently, made you want to quit smoking?**

2. **Had you tried to quit smoking in the past? (Yes or No)**

   IF YES, ***were you successful?***

   IF YES

a. **What prompted you to quit smoking then?**
b. **What helped you the most with this?**
c. **Why do you think you weren't able to successfully quit?**

IF NO

a. **Why do you think you have not tried to quit in the past?**
b. **What is different about your recent circumstances that you have tried to quit recently?**

3. **What characteristics or resources could help you quit next time?**

   Prompt (if needed):

   - *Aspects of your personality (e.g., determined, resilient)?*
   - *Aspects of your situation (e.g., family or friends who motivate you)?*
   - *How do these characteristics help you?*

4. **Is there anything else you think, had you had access to, would have helped you stop smoking?**

SECTION D: EXPERIENCES WITH SBIRT

I really appreciate you sharing all of that with me. Now, we are in the last section of my prepared questions, and they focus on your experiences with the support you received related to smoking cessation and varenicline.

If you could please take a moment to think back to when you were referred to SBIRT. You were screened to participate and then worked with a health worker who referred you to receive the varenicline. Specifically, your health worker discussed quitting smoking with you.

1. **How did that conversation go?**
   a. **Did you feel the health worker understood your situation and perspectives well?**
   b. **Did the health worker listen to and address your concerns and questions?**
   c. **Did you feel comfortable saying "no" to them?**
   d. **Did you feel like you had a good understanding of what you were agreeing to do?**

2. **Have you had additional check-ins or conversations with your health worker about managing your smoking?**
    a. **What aspects of these interactions were helpful?**

3. **Is there anything that your health worker could have done differently to make this experience better?**

Great, this has been really helpful.

Now, I want you to think about your experiences with varenicline.

4. **Were you prescribed a drug called Varenicline to take?**

    IF NO, Go to the closing section. IF YES,

5. **What was it like for you taking varenicline?**

    Prompts (if needed):

    - *Did you experience side effects? What were they like?*

6. **Is there anything that would have helped you better prepare for integrating varenicline into your routine?**

7. **Do you have any other recommendations for improving this intervention to better support smoking cessation?**

    Prompts (if needed):

    - *Anything your health worker could have done differently?*
    - *Anything the prescribing nurse could have done differently?*
    - *Anything other members of your support community could have done?*

SECTION E: CLOSING

I really appreciate all of your answers and everything you have shared with me today.

1. **Is there anything else that you feel we should have talked about regarding your experiences with trying to quit smoking?**

2. **Is there anything else you'd like to share about your experiences with the SBIRT intervention or varenicline?**

Those are all the prepared questions I have. Thank you for taking the time to talk with me today. As a reminder, all of your responses will always be kept confidential and will never be linked with your name or other identifying information.

Do you have any questions for me at this time? If anything comes up in the future, your consent form has contact information listed on it. Please feel free to contact us.

[after responding to any questions or concerns] **STOP RECORDING**

Appendix E2: SSIs for participants who quit and then resumed smoking

BSMART Participant Interview Guide

Participants Who Quit then Resumed Smoking

Note to Interviewer: **Text in red refers to important directions for you.**

SECTION A: INTRODUCTION

Thank you for taking the time to talk with me today. My name is ___________ and I am part of a team doing research on how we can help people quit smoking, especially when they encounter challenges when trying to do so.

Our conversation today will be quite informal, so please feel free to share anything you think would be important or helpful for us to know about your experiences with smoking and trying to quit.

I have a list of prepared questions that I will ask you. Sometimes I may ask you to expand or clarify your responses, I do this to make sure I fully understand. Other times I may ask that we move onto the next topic so that I can respect your time and ensure we can cover everything we need to get through.

You may or may not remember that I will be audio recording this interview. The reason for that is so that I can focus on what you are saying without trying to write it all down. No one will hear the recording except for people working on the project and we will remove any identifying information from the transcript of the interview. In reports from this project, we will never identify you as a participant or provide any information that allows other people to figure out you took part.

As a reminder, your participation in this interview is completely voluntary. If there are any questions you'd rather not answer, that is fine – just let me know. And, if at any point, you need a break, you can let me know that too. We are interested in your honest thoughts and experiences so there are no "right" or "wrong" answers to my questions.

Do you have any questions before we begin the interview?

[after responding to any questions or concerns] **START RECORDING**

Let's get started!

SECTION B: SMOKING EXPERIENCES

First, I want to ask you a little about your history with smoking tobacco. Please, can you take a moment to think back to when you began smoking.

8. **How old were you when you started smoking regularly?**

   Prompt (if needed):

   If participant cannot give an exact age, a ballpark number or some other indicator (e.g., a year in school; before/after a significant life event) works as well.

9. **What influenced you to begin smoking?**

   Prompt (if needed):

   - *What led you to start smoking?*
   - *Did you feel pressured by anyone to smoke?*

Now thinking about over the last week

10. **Tell me about when and how much you used to smoke before you last stopped.**

    Prompt (if needed):

    - *What time of day?*
    - *How many times on a typical day?*

11. **Does having HIV impact your smoking behaviors?**

    Prompt (if needed):

    - *Do you smoke more, less, or the same amount?*
    - *Why do you think that is?*

12. **What makes you smoke lately?**

    Prompt (if needed):

- *Pleasure, alleviate boredom*
- *Stress, manage depression or anxiety, manage anger*
- *Avoid withdrawal symptoms*
- *Manage pain or medications (e.g., make them work better, remove bad taste)*
- *Part of a social activity*
- *Weight management*
- *Need to (addicted to it); too hard to quit*
- *Avoid use of harder drugs or alcohol*
- *Helps with energy levels or concentration*
- *Not worried about health*

13. **Are these things that make you smoke now, different from the things that made you smoke when you first started?**

    Prompt (if needed):

    - *Why do you think that is?*

Now, when you think about these reasons for why you smoke [*note a few or all of them*]

14. **Which of these reasons for smoking made it most difficult for you to stay abstinent?**

SECTION C: SMOKING CESSATION

Thank you for everything you have shared so far. I want to ask you a bit more about your thoughts and experiences with quitting smoking.

5. **What reasons, recently, made you want to quit smoking?**

6. **Had you tried to quit smoking in the past?**

    IF YES

    a. What prompted you to quit smoking then?
    b. What helped you the most with this?

Now, thinking about your most recent attempt to quit smoking…

7. **What was different about your recent circumstances that made you try to quit recently?**

8. **Why do you think you weren't able to stay abstinent this time?**

9. **How ready did you feel to quit?**

10. **When you decided to stop smoking again this most recent time, how confident were you that you could succeed?**

11. **What characteristics or resources could help you stay abstinent next time?**

    Prompt (if needed):

    - *Aspects of your personality (e.g., determined, resilient)?*
    - *Aspects of your situation (e.g., family or friends who motivate you)?*
    - *How do these characteristics help you?*

12. **Are there other things, beside your personal characteristics, that helped you resist smoking as long as you did?**

    Prompt (if needed):

    - Support from others (e.g., care team, family, friends); Medications
    - Why do you think that was helpful?

13. **On the other hand, what challenges did you experience with staying abstinent?**

    9a. What do you think would help you to overcome these challenges in the future?

14. **Is there anything else you think, had you had access to, would have helped you stay abstinent for longer?**

SECTION D: EXPERIENCES WITH SBIRT

I really appreciate you sharing all of that with me. Now, we are in the last section of my prepared questions, and they focus on your experiences with the support you received related to smoking cessation and varenicline.

If you could please take a moment to think back to when you referred to SBIRT. You were screened to participate and then worked with a health worker who referred you to receive the varenicline. Specifically, your health worker discussed quitting smoking with you.

8. **How did that conversation go?**
    a. Did you feel the health worker understood your situation and perspectives well?
    b. Did the health worker listen to and address your concerns and questions?
    c. Did you feel comfortable saying "no" to them?
    d. Did you feel like you had a good understanding of what you were agreeing to do?

9. **Have you had additional check-ins or conversations with your health worker about managing your smoking?**
    a. What aspects of these interactions were helpful?
10. **Is there anything that your health worker could have done differently to make this experience better?**

Great, this has been really helpful. Now, I want you to think about your experiences with varenicline.

11. **Were you prescribed a drug called Varenicline to take?**

    IF NO, Go to the closing section. IF YES,

12. **What was it like for you taking varenicline?**

    Prompts (if needed):

    - *Did you experience side effects?*
    - *What were they like?*

13. **Is there anything that would have helped you better prepare for integrating varenicline into your routine?**

14. **Do you have any other recommendations for improving this intervention to better support smoking cessation?**

Prompts (if needed):

- *Anything your health worker could have done differently?*
- *Anything the prescribing nurse could have done differently?*
- *Anything other members of your support community could have done?*

SECTION E: CLOSING

I really appreciate all of your answers and everything you have shared with me today.

3. **Is there anything else that you feel we should have talked about regarding your experiences with trying to quit smoking?**

4. **Is there anything else you'd like to share about your experiences with the SBIRT intervention or varenicline?**

Those are all the prepared questions I have. Thank you for taking the time to talk with me today. As a reminder, all of your responses will always be kept confidential and will never be linked with your name or other identifying information.

Do you have any questions for me at this time? If anything comes up in the future, your consent form has contact information listed on it. Please feel free to contact us.

[after responding to any questions or concerns] **STOP RECORDING**

Appendix E3: SSIs for participants who have quit smoking

BSMART Participant Interview Guide

Participants Who are Currently not Smoking

Note to Interviewer:<br>**Text in red refers to important directions for you.**

SECTION A: INTRODUCTION

Thank you for taking the time to talk with me today. My name is ___________ and I am part of a team doing research on how we can help people quit smoking, especially when they encounter challenges when trying to do so.

Our conversation today will be quite informal, so please feel free to share anything you think would be important or helpful for us to know about your experiences with smoking and trying to quit. I have a list of prepared questions that I will ask you. Sometimes I may ask you to expand or clarify your responses, I do this to make sure I fully understand. Other times I may ask that we move onto the next topic so that I can respect your time and ensure we can cover everything we need to get through.

You may or may not remember that I will be audio recording this interview. The reason for that is so that I can focus on what you are saying without trying to write it all down. No one will hear the recording except for people working on the project and we will remove any identifying information from the transcript of the interview. In reports from this project, we will never identify you as a participant or provide any information that allows other people to figure out you took part.

As a reminder, your participation in this interview is completely voluntary. If there are any questions you'd rather not answer, that is fine – just let me know. And, if at any point, you need a break, you can let me know that too. We are interested in your honest thoughts and experiences so there are no "right" or "wrong" answers to my questions.

Do you have any questions before we begin the interview?

[after responding to any questions or concerns] **START RECORDING**

Let's get started!

SECTION B: SMOKING EXPERIENCES

First, I want to ask you a little about your history with smoking tobacco. Please, can you take a moment to think back to when you began smoking.

15. How old were you when you started smoking regularly?

Prompt (if needed):

If participant cannot give an exact age, a ballpark number or some other indicator (e.g., a year in school; before/after a significant life event) works as well

16. What influenced you to begin smoking?

Prompt (if needed):

- *What led you to start smoking?*
- *Did you feel pressured by anyone to smoke?*

Now thinking about over the last week

17. Tell me about when and how much you used to smoke before you last stopped?

Prompt (if needed):

- *What time of day?*
- *How many times on a typical day?*

18. When you smoked, did having HIV impact your smoking behaviors?

Prompt (if needed):

- *Do you smoke more, less, or the same amount?*
- *Why do you think that is?*

19. **What made you smoke (before you stopped)?**

Prompt (if needed):

- *Pleasure, alleviate boredom*
- *Stress, manage depression or anxiety, manage anger*
- *Avoid withdrawal symptoms*
- *Manage pain or medications (e.g., make them work better, remove bad taste)*
- *Part of a social activity*
- *Weight management*
- *Need to (addicted to it); too hard to quit*
- *Avoid use of harder drugs or alcohol*
- *Helps with energy levels or concentration*
- *Not worried about health*

SECTION C: SMOKING CESSATION

Thank you for everything you have shared so far. I want to ask you a bit more about your thoughts and experiences with quitting smoking.

15. **What reasons, recently, made you want to quit smoking?**
16. **What helped you the most with this?**

Now, thinking about your most recent attempt to quit smoking…

17. **How ready did you feel to quit?**
18. **When you decided to stop smoking again this most recent time, how confident were you that you could succeed?**
    a. How capable did you feel?
    b. How capable do you feel now?
19. **What characteristics or resources do you have that you think helped you with quitting?**

Prompt (if needed):

- *Aspects of your personality (e.g., determined, resilient)?*
- *Aspects of your situation (e.g., family or friends who motivate you)?*

- *How do these characteristics help you?*

20. **Are there other things, beside your personal characteristics, that helped you quit and stay off smoking as long as you have?**

    Prompt (if needed):

    - *Support from others (e.g., care team, family, friends); Medications*
    - *Why do you think that was helpful?*

21. **On the other hand, what challenges did you experience with quitting?**
    a. How did you overcome these challenges?

22. **Is there anything else you think, had you had access to, would have helped you stop smoking sooner? Or would have made it easier for you to quit?**

SECTION D: EXPERIENCES WITH SBIRT

I really appreciate you sharing all of that with me. Now, we are in the last section of my prepared questions, and they focus on your experiences with the support you received related to smoking cessation and varenicline.

If you could please take a moment to think back to when you referred to SBIRT. You were screened to participate and then worked with a health worker who referred you to receive the varenicline. Specifically, your health worker discussed quitting smoking with you.

15. **How did that conversation go?**
    a. Did you feel the health worker understood your situation and perspectives well?
    b. Did the health worker listen to and address your concerns and questions?
    c. Did you feel comfortable saying “no” to them?
    d. Did you feel like you had a good understanding of what you were agreeing to do?

16. **Have you had additional check-ins or conversations with your health worker about managing your smoking?**
    a. What aspects of these interactions were helpful?

17. **Is there anything that your health worker could have done differently to make this experience better?**

Great, this has been really helpful. Now, I want you to think about your experiences with varenicline.

18. **Were you prescribed a drug called Varenicline to take?**

    IF NO, Go to the closing section. IF YES,

19. **What was it like for you taking varenicline?**

    Prompts (if needed):

    - Did you experience side effects?
    - What were they like?

20. **Is there anything that would have helped you better prepare for integrating varenicline into your routine?**

21. **Do you have any other recommendations for improving this intervention to better support smoking cessation?**

    Prompts (if needed):

    - Anything your health worker could have done differently?
    - Anything the prescribing nurse could have done differently?
    - Anything other members of your support community could have done?

SECTION E: CLOSING

I really appreciate all of your answers and everything you have shared with me today.

5. **Is there anything else that you feel we should have talked about regarding your experiences with quitting smoking?**

6. **Is there anything else you'd like to share about your experiences with the SBIRT intervention or varenicline?**

Those are all the prepared questions I have. Thank you for taking the time to talk with me today. As a reminder, all of your responses will always be kept confidential and will never be linked with your name or other identifying information.

Do you have any questions for me at this time? If anything comes up in the future, your consent form has contact information listed on it. Please feel free to contact us.

[after responding to any questions or concerns] **STOP RECORDING**

Appendix F: Activity Log:

Date: ____________________ Lay case manager ID: ____________________

Number of patients invited to enroll ____________ Number enrolled ____________

	**Duration of counselling**	**Outcome of counselling (agreed to participate; declined)**	**Referred to NPT (N/A; YES; NO)**	**Outcome of referral**	**Challenges**
Participant 1					
Participant 2					
Participant 3					
Participant 4					

The logs will collect information about implementation activities, intent, duration, and individuals involved.

Appendix G: Program Sustainability Tool

A. Prepare and Assess Your Program

1. Define the program being assessed and identify participants.
2. Complete the Program Sustainability Assessment Tool.

↓

B. Develop an Action Plan

1. Assemble the planning team.
2. Review your program's mission and purpose.
3. Review Program Sustainability Assessment Tool results.
4. Determine which program elements need to be maintained, eliminated, or adapted.
5. Prioritize the areas of sustainability capacity to address first.
6. Write an action plan with specific action steps to strengthen and build your program's sustainability capacity.

↓

C. Take Action

1. Implement the action plan.
2. Reassess sustainability capacity annually.

Appendix H: Tools for Collecting Fixed and Variable Costs

Fixed Costs	Variable costs
Training	Staff time to deliver SOC
Lay Health Workers	Staff time to deliver BSMART
Research Assistants	
Nurse prescribers	
Cost of SOC brochure	
Cost of data collection tools	

Training Costs	Cost of training materials
	Cost of training venue
Cost of trainer(s)	Cost of trainers' time
	Transportation
	Per diem+
Trainee costs	Transport
	feeding

Cost of SOC	
Staff Time	Hourly wage
	Time spent
Cost of brochure	

Cost of BSMART	
Staff cost for counseling	Hourly wage
	Time spent
Cost of CO monitoring	coVita CO Smokerlyzer
	Staff time
	Hourly wage
Cost of varenicline use	Varenicline dose (for 12 weeks)
	Staff time for participant assessment for eligibility for varenicline use
	Patient transportation for drug pick ups
	Cost of managing adverse events
	Staff time for adverse event management
Cost of follow-up visits	Participant transport weekly for the first four weeks and monthly for two months then at week 24
	Staff costs for follow-up visits

Appendix I: Standard of Care Brochure

You can quit smoking

If you have tried to quit before and were not successful **don't give up!** Research has shown that it takes an average of 6-8 quit attempts for a smoker to quit for good. The idea is to **work through it!**

Craving tobacco?

D - delay
for 10-15 minutes and keep increasing the delay time as days go by

E - escape
remove yourself from the trigger/stressful situation

A - avoid
being in tempting areas

D - distract
get busy

S - substitute
chew on something
(straw, toothpick, sugar free gum)

CONTACT US

Anti-Tobacco Network in partnership with University of Botswana

(+267) 3354134

M: 75563646

WHATSAPP: 75563646

Do you want to **quit smoking**?

Tobacco is the only legal consumer product that **kills** up to half of its users when used exactly as intended by the manufacturer.

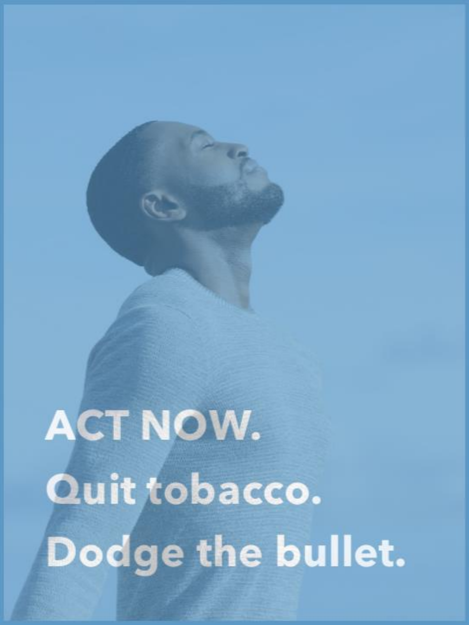

Did you know that when you smoke...?

- People with HIV are **more likely** to develop **harmful consequences of smoking** than those without HIV
- You are **more likely** to get pneumonia and suffer from asthma attacks
- You are **more likely** to suffer from cancer
- You are **more likely** to develop severe disease from COVID-19
- You **spend money** that you could use for other things
- You **harm** the health of your children and those around you

The good news is that when you quit...

- You will be **less likely** to get pneumonia, asthma attacks, and cancer
- You will **save** a lot of money
- You will **protect** the health of your children and those around you

Things you may feel when you quit & what to do

Symptoms	What To Do
Irritability	Avoid stress, practice relaxation techniques, exercise for 30 minutes
Depressed mood	Do something fun, talk to someone (friend, family, therapist)
Dizziness	Get up slowly from sitting positions
Difficulty in concentrating	Avoid stress, use a day planner, break your work into small portions
Chest tightness	Practice relaxation techniques
Fatigue	Get more sleep, take naps, don't push yourself, rest
Hunger	Drink lots of water, eat low-calorie snacks (fruit)
Stomach pain, constipation, gas	Drink fluids, eat fruits and vegetables
Cough, dry throat, runny nose	Use cough drops/syrup, eat sugar free candy, drink fluids
Difficulty sleeping	Reduce caffeine consumption and intake of energy drinks or coffee

What is the best way to quit tobacco use?

1. Ask yourself why you want to quit

2. Figure out why you use tobacco, because it is a learned behavior

3. Know your triggers or the things that drive you to use tobacco

4. Address your triggers and try not to use tobacco during one of your triggers by doing something else
    - Going for a walk
    - Drinking water

5. Change where you normally keep your tobacco products

6. Keep track of every time you use tobacco, along with the triggers, moods, and the level of need for tobacco use.

Appendix J: BoMRA Waiver
