# Supplementary material for: Design and rationale of the Botswana Smoking Abstinence Reinforcement Trial: a protocol for a stepped-wedge cluster randomized trial
Source: Implement Sci Commun. 2024 May 8;5:53. doi: 10.1186/s43058-024-00588-7 (PMC11077839; doi:10.1186/s43058-024-00588-7)
Supplement: Supplementary file 1 — Supplementary Material 1. [file 43058_2024_588_MOESM1_ESM.zip › BSMART UMB Approval of continuing applicationR0.pdf]

University of Maryland, Baltimore
Institutional Review Board (IRB)
Phone: (410) 706-5037
Fax: (410) 706-4189
Email: hrpo@umaryland.edu

APPROVAL OF RESEARCH NOTIFICATION

The IRB and the HRPO, as part of the Office of Accountability and Compliance is committed to excellence and customer service. Please take a moment to tell us how we are doing: HRPO/IRB/OAC Customer Feedback Survey

---

Date: November 20, 2023

To: Manhattan Charurat
RE: HCR-HP-00102995-1
Type of Submission: Continuing Review
Type of IRB Review: Full Board

Approval for this project is valid from 11/14/2023 to 11/13/2024

This is to certify that the University of Maryland, Baltimore (UMB) Institutional Review Board (IRB) approved the continuing review report for the above referenced protocol entitled, "*Botswana Smoking and Abstinence Reinforcement Trial*".

The IRB made the following determinations regarding this submission:
- Written informed consent is required. Only the valid IRB-approved informed consent form(s) in CICERO can be used.

This study is approved to enroll 0 local participants.

This study is approved to enroll 750 worldwide participants.

Below is a list of the documents attached to your application that have been approved:
BSMART DSMB Letter Response
Eligibility Checklist for HP-00102995_1 v2-23-2023-1677166316696
Botswana Smoking Abstinence Reinforcement Trial: A Stepped Wedge Cluster Randomized Trial
BSMART Protocol v4_Clean version
BSMART Protocol_v5_Tracked
BSMART Protocol_v5
BSMART Protocol_v5.1_tracked
BSMART Protocol_v5.1
NOA_BSMART study
102995 IAA-UMB-Charurat_-_UMBC-Diclemente_BSMART_PE_1.24.2023.pdf
Determination - 8483_Charurat_ BSMART.pdf
Screen shot of data fields from Botswana EMR
Varenicline Package Insert

BSMART_SSI for participants who did not quit smoking.pdf
BSMART_SSI for participants who have quit smoking.pdf
BSMART_SSI for participants who quit then resumed smoking.pdf
Focus Group Guide for Lay Health Workers.pdf
Focus Group Guide for Nurse Prescribers.pdf
Follow-uo Smoking Questionnaire_June06_2023_clean
Follow-uo Smoking Questionnaire_June06_2023_tracked
Intake Smoking History Questionnaire_June6_tracked
Intake smoking History Questionnaire_June6_2023_tracked
Intake smoking History Questionnaire
Follow-up Smoking Questionnaire
DSMB Charter_BSMART
BSMART Setswana translation certificates
English ICF_Control Phase
English ICF_Intervention Phase
English ICF_FGD with LHW
English ICF_FGD with NPD
BoMRA Apo-varenicline 1mg waiver
English ICF for cost data from LHWs and NPDs
Setswana Translations
BoMRA Apo-varenicline 0.5mg waiver
Setswana ICF_Control Phase
English ICF_Control Phase
Setswana Intake Smoking History Questionnaire_June062023_tracked
Setswana ICF_Intervention phase
English ICF_Intervention Phase
Setswana ICF_FGD with LHW
English ICF_FGD with LHW
Setswana Intake Smoking HistoryQuestionnaire_June062023_clean
Setswana ICF_FGD with NPD
Setswana ICF_Collection of costs from LHWs and NPD
English ICF_FGD with NPD
English ICF_Collection of costs from LHWs and NPD
English ICF_Control Phase_tracked
BSMART translation certificate for modified forms
English ICF_Intervention Phase_tracked
English ICF_FGD with LHW_tracked
Setswana Follow-up Smoking Questionnaire_tracked
English ICF_FGD with NPD_tracked
English ICF_Collection of costs from LHWs and NPD_tracked
Setswana Follow-up Smoking Questionnaire_June062023_clean

In conducting this research you are required to follow the requirements listed in the INVESTIGATOR MANUAL. Investigators are reminded that the IRB must be notified of any changes in the study. In addition, the PI is responsible for ensuring prompt reporting to the IRB of proposed changes in a research activity, and for ensuring that such changes in approved research, during the period for which IRB approval has already been given, may not be initiated without IRB review and approval except when necessary to eliminate apparent immediate hazards to the subject (45 CFR 46.103(4)(iii)). The PI must also inform the IRB of any new and significant information that may impact a research participant's safety or willingness to continue in the study and any unanticipated problems involving risks to participants or others.

DHHS regulations at 45 CFR 46.109 (e) require that **continuing review** of research be conducted by the IRB at intervals appropriate to the degree of risk and **not less than once per year.** The regulations make **no provision for any grace period extending the conduct of the research beyond 11/13**/2024. You will receive continuing review email reminder notices prior to this date; however, it is your responsibility to submit your continuing review report in a timely manner to allow adequate time for substantive and meaningful IRB review and assure that this study is not conducted beyond **11/13/2024**. Investigators should submit continuing review reports in the electronic system at least <u>six weeks prior</u> to this date.

Research activity in which the VA Maryland Healthcare System (VAMHCS) is a recruitment site or in which VA resources (i.e., space, equipment, personnel, funding, data) are otherwise involved, must also be approved by the VAMHCS Research and Development Committee prior to initiation at the VAMHCS. Contact the VA Research Office at 410-605-7000 ext. 6568 for assistance.

The UMB IRB is organized and operated according to guidelines of the International Council on Harmonization, the United States Office for Human Research Protections and the United States Code of Federal Regulations and operates under Federal Wide Assurance No. FWA00007145.

If you have any questions about this review or questions, concerns, and/or suggestions regarding the Human Research Protection Program (HRPP), please do not hesitate to contact the Human Research Protections Office (HRPO) at (410) 706-5037 or HRPO@umaryland.edu.
