# Supplementary material for: Design and rationale of the Botswana Smoking Abstinence Reinforcement Trial: a protocol for a stepped-wedge cluster randomized trial
Source: Implement Sci Commun. 2024 May 8;5:53. doi: 10.1186/s43058-024-00588-7 (PMC11077839; doi:10.1186/s43058-024-00588-7)
Supplement: Supplementary file 1 — Supplementary Material 1. [file 43058_2024_588_MOESM1_ESM.zip › BSMART_NOA_1U01CA275048-01R0.pdf]

Department of Health and Human Services
National Institutes of Health
NATIONAL CANCER INSTITUTE

Notice of Award

FAIN# U01CA275048
Federal Award Date
09-15-2022

Recipient Information

1. Recipient Name
UNIVERSITY OF MARYLAND
220 ARCH ST RM 02148

BALTIMORE, 21201

2. Congressional District of Recipient
07

3. Payment System Identifier (ID)
1526002036A1

4. Employer Identification Number (EIN)
526002036

5. Data Universal Numbering System (DUNS)
188435911

6. Recipient's Unique Entity Identifier
Z9CRZKD42ZT1

7. Project Director or Principal Investigator
Manhattan E Charurat, PHD (Contact)
Professor And Director
mcharurat@ihv.umaryland.edu
410-706-1948

8. Authorized Official
Christine R. Toalepai

Federal Agency Information

9. Awarding Agency Contact Information
Ashley Michelle Utter

NATIONAL CANCER INSTITUTE
ashley.utter@nih.gov
240-276-5635

10. Program Official Contact Information
Vidya Vedham

NATIONAL CANCER INSTITUTE
vidya.vedham@nih.gov
240-276-7272

Federal Award Information

11. Award Number
1U01CA275048-01

12. Unique Federal Award Identification Number (FAIN)
U01CA275048

13. Statutory Authority
42 USC 241 31 USC 6305 42 CFR 52

14. Federal Award Project Title
Botswana Smoking Abstinence Reinforcement Trial (BSMART)

15. Assistance Listing Number
93.393

16. Assistance Listing Program Title
Cancer Cause and Prevention Research

17. Award Action Type
New Competing

18. Is the Award R&D?
Yes

Summary Federal Award Financial Information

**19. Budget Period Start Date** 09-15-2022 **– End Date** 08-31-2023	
**20. Total Amount of Federal Funds Obligated by this Action**	$754,687
20 a. Direct Cost Amount	$544,926
20 b. Indirect Cost Amount	$209,761
**21.** Authorized Carryover	
**22.** Offset	
**23.** Total Amount of Federal Funds Obligated this budget period	$754,687
**24. Total Approved Cost Sharing or Matching, where applicable**	$0
**25. Total Federal and Non-Federal Approved this Budget Period**	$754,687
**26. Project Period Start Date** 09-15-2022 **– End Date** 08-31-2027	
**27.** Total Amount of the Federal Award including Approved Cost Sharing or Matching this Project Period	$754,687

28. Authorized Treatment of Program Income
Additional Costs

29. Grants Management Officer - Signature
Ashley Michelle Utter

30. Remarks
Acceptance of this award, including the "Terms and Conditions," is acknowledged by the recipient when funds are drawn down or otherwise requested from the grant payment system.

SECTION I – AWARD DATA – 1U01CA275048-01

Principal Investigator(s):
Manhattan E Charurat (contact), PHD
SETH S HIMELHOCH, MD
BONTLE MBONGWE

Award e-mailed to: nga@umaryland.edu

Dear Authorized Official:

The National Institutes of Health hereby awards a grant in the amount of $754,687 (see "Award Calculation" in Section I and "Terms and Conditions" in Section III) to UNIVERSITY OF MARYLAND BALTIMORE in support of the above referenced project. This award is pursuant to the authority of 42 USC 241 31 USC 6305 42 CFR 52 and is subject to the requirements of this statute and regulation and of other referenced, incorporated or attached terms and conditions.

Acceptance of this award, including the "Terms and Conditions," is acknowledged by the recipient when funds are drawn down or otherwise requested from the grant payment system.

Each publication, press release, or other document about research supported by an NIH award must include an acknowledgment of NIH award support and a disclaimer such as "Research reported in this publication was supported by the National Cancer Institute of the National Institutes of Health under Award Number U01CA275048. The content is solely the responsibility of the authors and does not necessarily represent the official views of the National Institutes of Health." Prior to issuing a press release concerning the outcome of this research, please notify the NIH awarding IC in advance to allow for coordination.

Award recipients must promote objectivity in research by establishing standards that provide a reasonable expectation that the design, conduct and reporting of research funded under NIH awards will be free from bias resulting from an Investigator's Financial Conflict of Interest (FCOI), in accordance with the 2011 revised regulation at 42 CFR Part 50 Subpart F. The Institution shall submit all FCOI reports to the NIH through the eRA Commons FCOI Module. The regulation does not apply to Phase I Small Business Innovative Research (SBIR) and Small Business Technology Transfer (STTR) awards. Consult the NIH website http://grants.nih.gov/grants/policy/coi/ for a link to the regulation and additional important information.

If you have any questions about this award, please direct questions to the Federal Agency contacts.

Sincerely yours,

Ashley Michelle Utter
Grants Management Officer
NATIONAL CANCER INSTITUTE

Additional information follows

Cumulative Award Calculations for this Budget Period (U.S. Dollars)

**Salaries and Wages**	$177,154
**Fringe Benefits**	$69,119
**Personnel Costs (Subtotal)**	$246,273
**Consultant Services**	$8,000
**Equipment**	$12,000
**Materials & Supplies**	$10,969
**Travel**	$11,130
**Other**	$8,510
**Subawards/Consortium/Contractual Costs**	$248,044

**Federal Direct Costs**	$544,926
**Federal F&A Costs**	$209,761
**Approved Budget**	$754,687
**Total Amount of Federal Funds Authorized (Federal Share)**	$754,687
**TOTAL FEDERAL AWARD AMOUNT**	$754,687

**AMOUNT OF THIS ACTION (FEDERAL SHARE)**	$754,687

SUMMARY TOTALS FOR ALL YEARS (for this Document Number)

YR	THIS AWARD	CUMULATIVE TOTALS
1	$754,687	$754,687
2	$701,239	$701,239
3	$699,789	$699,789
4	$710,257	$710,257
5	$713,194	$713,194

Recommended future year total cost support, subject to the availability of funds and satisfactory progress of the project

Fiscal Information:

Payment System Identifier: 1526002036A1
Document Number: UCA275048A
PMS Account Type: P (Subaccount)
Fiscal Year: 2022

IC	CAN	2022	2023	2024	2025	2026
CA	8479565	$754,687	$701,239	$699,789	$710,257	$713,194

Recommended future year total cost support, subject to the availability of funds and satisfactory progress of the project

NIH Administrative Data:
PCC: AEGH / **OC**: 41026 / **Released**: Utter, Ashley 09-08-2022
Award Processed: 09/15/2022 12:21:18 AM

SECTION II – PAYMENT/HOTLINE INFORMATION – 1U01CA275048-01

For payment and HHS Office of Inspector General Hotline information, see the NIH Home Page at http://grants.nih.gov/grants/policy/awardconditions.htm

SECTION III – STANDARD TERMS AND CONDITIONS – 1U01CA275048-01

This award is based on the application submitted to, and as approved by, NIH on the above-titled project and is subject to the terms and conditions incorporated either directly or by reference in the following:

a. The grant program legislation and program regulation cited in this Notice of Award.
b. Conditions on activities and expenditure of funds in other statutory requirements, such as those included in appropriations acts.
c. 45 CFR Part 75.
d. National Policy Requirements and all other requirements described in the NIH Grants Policy Statement, including addenda in effect as of the beginning date of the budget period.
e. Federal Award Performance Goals: As required by the periodic report in the RPPR or in the final progress report when applicable.
f. This award notice, INCLUDING THE TERMS AND CONDITIONS CITED BELOW.

(See NIH Home Page at http://grants.nih.gov/grants/policy/awardconditions.htm for certain references cited above.)

Research and Development (R&D): All awards issued by the National Institutes of Health (NIH) meet the definition of "Research and Development" at 45 CFR Part§ 75.2**.** As such, auditees should identify NIH awards as part of the R&D cluster on the Schedule of Expenditures of Federal Awards (SEFA). The auditor should test NIH awards for compliance as instructed in Part V, Clusters of Programs. NIH recognizes that some awards may have another classification for purposes of indirect costs. The auditor is not required to report the disconnect (i.e., the award is classified as R&D for Federal Audit Requirement purposes but non-research for indirect cost rate purposes), unless the auditee is charging indirect costs at a rate other than the rate(s) specified in the award document(s).

Carry over of an unobligated balance into the next budget period requires Grants Management Officer prior approval.

This award is subject to the requirements of 2 CFR Part 25 for institutions to obtain a unique entity identifier (UEI) and maintain an active registration in the System for Award Management (SAM). Should a consortium/subaward be issued under this award, a UEI requirement must be included. See http://grants.nih.gov/grants/policy/awardconditions.htm for the full NIH award term implementing this requirement and other additional information.

This award has been assigned the Federal Award Identification Number (FAIN) U01CA275048. Recipients must document the assigned FAIN on each consortium/subaward issued under this award.

Based on the project period start date of this project, this award is likely subject to the Transparency Act subaward and executive compensation reporting requirement of 2 CFR Part 170. There are conditions that may exclude this award; see http://grants.nih.gov/grants/policy/awardconditions.htm for additional award applicability information.

In accordance with P.L. 110-161, compliance with the NIH Public Access Policy is now mandatory. For more information, see NOT-OD-08-033 and the Public Access website: http://publicaccess.nih.gov/.

This award provides support for one or more clinical trials. By law (Title VIII, Section 801 of Public Law 110-85), the "responsible party" must register "applicable clinical trials" on the ClinicalTrials.gov Protocol Registration System Information Website. NIH encourages registration of all trials whether required under the law or not. For more information, see http://grants.nih.gov/ClinicalTrials_fdaaa/

In accordance with the regulatory requirements provided at 45 CFR 75.113 and Appendix XII to 45 CFR Part 75, recipients that have currently active Federal grants, cooperative agreements, and procurement contracts with cumulative total value greater than $10,000,000 must report and maintain information in the System for Award Management (SAM) about civil, criminal, and administrative proceedings in connection with the award or performance of a Federal award that reached final disposition within the most recent five-year period. The recipient must also make semiannual disclosures regarding such proceedings. Proceedings information will be made publicly available in the designated integrity and performance system (currently the Federal Awardee Performance and Integrity Information System (FAPIIS)). Full reporting requirements and procedures are found in Appendix XII to 45 CFR Part 75. This term does not apply to NIH fellowships.

Treatment of Program Income:

Additional Costs

SECTION IV – CA SPECIFIC AWARD CONDITIONS – 1U01CA275048-01

Clinical Trial Indicator: Yes
This award supports one or more NIH-defined Clinical Trials. See the NIH Grants Policy Statement Section 1.2 for NIH definition of Clinical Trial.

RESTRICTION: This provisional award is issued subject to the following special condition(s):

The present award is being made without currently valid verification of IRB approval for this project with the following restriction: Only activities that are clearly severable and independent from activities that involve human subjects may be conducted pending the National Cancer Institute (NCI) acceptance of the certification of IRB review and approval. The verification of IRB approval must be submitted no later than November 30th of this year, to the specialist named in this award via email ashley.utter@nih.gov with a copy to NCIOGAProgressReports@mail.nih.gov.

No funds may be drawn down from the payment system and no obligations may be made against Federal funds for any research involving human subjects prior to the NCI's notification to the recipient that the identified issues have been resolved and this restriction removed.

The recipient institution may conduct only activities that are clearly severable and independent from the activities that involve human subjects until OHRP has approved an assurance and the NCI has received and accepted the recipient institution’s certification of IRB approval.

Failure to submit the IRB approval to NCI within the required timeframe or to otherwise comply with the above requirements can result in suspension and/or termination of this award, withholding of support, audit disallowances, and/or other appropriate action.

REQUIREMENT: The clinical trial(s) supported by this award is subject to the plan dated 12/14/2021 submitted to NIH and the NIH policy on Dissemination of NIH-Funded Clinical Trial Information. The plan states that the clinical trial(s) funded by this award will be registered in ClinicalTrials.gov not later than 21 calendar days after enrollment of the first participant and primary summary results reported in ClinicalTrials.gov, not later

than one year after the completion date. The reporting of summary results is required by this term of award even if the primary completion date occurs after the period of performance.

REQUIREMENT: This award is subject to additional certification requirements with each submission of the Annual, Interim, and Final Research Performance Progress Report (RPPR). The recipient must agree to the following annual certification when submitting each RPPR. By submitting the RPPR, the AOR signifies compliance, as follows:
In submitting this RPPR, the SO (or PD/PI with delegated authority), certifies to the best of his/her knowledge that, for all clinical trials funded under this NIH award, the recipient and all investigators conducting NIH-funded clinical trials are in compliance with the recipient's plan addressing compliance with the NIH Policy on Dissemination of NIH-Funded Clinical Trial Information. Any clinical trial funded in whole or in part under this award has been registered in ClinicalTrials.gov or will be registered not later than 21 calendar days after enrollment of the first participant. Summary results have been submitted to ClinicalTrials.gov or will be submitted not later than one year after the completion date, even if the completion date occurs after the period of performance.

REQUIREMENT: The awardee is required to follow the data and safety monitoring plan included in the application and may not implement any changes in the plan without the written prior approval of the National Cancer Institute.

REQUIREMENT: The awardee is required to follow the Data Sharing plan included in the competing application and may not implement any changes in the plan without the written prior approval of the National Cancer Institute.

REQUIREMENT: This award is issued as a cooperative agreement, a financial assistance mechanism in which substantial NIH scientific and/or programmatic involvement is anticipated in the performance of the activity. This award is subject to the Terms and Conditions of Award as set forth in RFA CA21-056, "Implementation Science for Cancer Control in People Living with HIV in Low- and Middle-Income Countries (U01 Clinical Trial Optional)," NIH Guide to Grants and Contracts, 08/25/2021, which are hereby incorporated by reference as special terms and conditions of this award.
Copies of this RFA may be accessed at the following internet address:
http://www.nih.gov/grants/guide/index.html

Copies may also be obtained from the Grants Management Contact indicated in the terms of award.

These special Terms and Conditions of Award are in addition to and not in lieu of otherwise applicable OMB administrative guidelines, Federal Regulations, including HHS Grant Administration Regulations at 42 CFR Part 52, 45 CFR Parts 75 and 92, and other HHS, PHS, and NIH grants policy statements.

The following administrative terms also apply:

INFORMATION: This award involves Human Subjects Research. See "Assurance Requirements and Institutional Review Boards" under Part II, Subpart A, Human Subjects, in the NIH Grants Policy Statement, for specific requirements and recipient responsibilities related to the protection of human subjects, which are applicable to and are a term and condition of this award.

This award reflects the National Cancer Institute's acceptance of the certification that all key personnel have completed education on the protection of human subjects, in accordance with the NIH Grants Policy Statement, "Education in the Protection of Human Research Subjects."

Any individual involved in the design and conduct of the study that is not included in the certification must satisfy this requirement prior to participating in the project. Failure to comply can result in the suspension and/or termination of this award, withholding of support of the continuation award, audit disallowances, and/or other appropriate action.

INFORMATION: Although the budget period start date for this award is 09/15 this award includes funds for twelve months of support. Future year budget periods will cycle on 09/01. Allowable pre-award costs may be charged to this award, in accordance with the conditions in the NIH Grants Policy Statement, and with institutional requirements for prior approval.

INFORMATION: This award, including the budget and the budget period, has been discussed between Ashley Utter of the National Cancer Institute and Christine To'alepai

SPREADSHEET SUMMARY
AWARD NUMBER: 1U01CA275048-01

INSTITUTION: UNIVERSITY OF MARYLAND BALTIMORE

Budget	Year 1	Year 2	Year 3	Year 4	Year 5
Salaries and Wages	$177,154	$177,154	$177,154	$196,583	$197,369
Fringe Benefits	$69,119	$69,119	$69,119	$69,457	$68,513
Personnel Costs (Subtotal)	$246,273	$246,273	$246,273	$266,040	$265,882
Consultant Services	$8,000	$8,000	$6,400	$7,200	$7,200
Equipment	$12,000				
Materials & Supplies	$10,969	$10,084	$10,112	$10,401	$1,032
Travel	$11,130	$12,090	$12,090	$12,090	$16,178
Other	$8,510	$8,190	$6,190	$7,386	$6,536
Subawards/Consortium/Contractual Costs	$248,044	$261,475	$265,544	$241,941	$229,875
Publication Costs					$16,000
TOTAL FEDERAL DC	$544,926	$546,112	$546,609	$545,058	$542,703
TOTAL FEDERAL F&A	$209,761	$155,127	$153,180	$165,199	$170,491
TOTAL COST	$754,687	$701,239	$699,789	$710,257	$713,194

Facilities and Administrative	Year 1	Year 2	Year 3	Year 4	Year 5

Costs					
F&A Cost Rate 1	54.5%	54.5%	54.5%	54.5%	54.5%
F&A Cost Base 1	$384,882	$284,637	$281,065	$303,117	$312,828
F&A Costs 1	$209,761	$155,127	$153,180	$165,199	$170,491
