# Supplementary material for: Design and rationale of the Botswana Smoking Abstinence Reinforcement Trial: a protocol for a stepped-wedge cluster randomized trial
Source: Implement Sci Commun. 2024 May 8;5:53. doi: 10.1186/s43058-024-00588-7 (PMC11077839; doi:10.1186/s43058-024-00588-7)
Supplement: Supplementary file 1 — Supplementary Material 1. [file 43058_2024_588_MOESM1_ESM.zip › Spirit Checklist for the BSMART Study_4.8.2024R0.pdf]

Spirit Checklist for the BSMART Study

Section/Item	Item No	Description
**Administrative Information**		
**Title**	1	Design and rationale of the Botswana smoking abstinence reinforcement trial (BSMART): a protocol for a stepped-wedge cluster randomized trial
**Trial Registration**	2a	**Clinical Trial Registration:** NCT05694637<br>Registered on 7 December 2022 on clinicaltrials.gov, https://clinicaltrials.gov/search?locStr=Botswana&country=Botswana&cond=Smoking%20Cessation&intr=SBIRT
	2b	**All items from the World Health Organization Trial Registration Data**<br>The study is registered on ClinicalTrials.gov.
**Protocol version**	3	Protocol version 5.1 June 6, 2023
**Funding**	4	This study has been supported by funding from the National Cancer Institute at the National Institutes of Health (U01CA275048).
**Roles and Responsibilities**	5a	***Names and Affiliations of protocol contributors***<br><br>Florence Bada, PhD, MB/BS[1], Megan E. Mansfield[1], Lillian Okui, MBChB, MPH[2], Milton Montebatsi, MD[2], Carlo DiClemente, PhD[3], Roy Tapera, PhD, MPH[4,5], Kaizer Ikgopoleng[2], Selebaleng Mokonopi[2] Jessica F. Magidson, PhD[6], Eberechukwu Onukwugha, PhD[7], Ndwapi Ndwapi, MD[2], Seth Himelhoch, MD, MPH[8], Bontle Mbongwe, PhD, MSc[4,5], Man Charurat, PhD, MHS[1,9]<br><br>1. Division of Epidemiology and Prevention, Institute of Human Virology, University of Maryland School of Medicine, Baltimore, Maryland, U.S.A<br>2. Botswana University of Maryland Medicine Health Initiative, Gaborone, Botswana<br>3. Department of Psychology, University of Maryland Baltimore County, Baltimore, Maryland, U.S.A<br>4. University of Botswana School of Public Health, Gaborone, Botswana<br>5. Anti-tobacco Network, University of Botswana, Gaborone, Botswana<br>6. Department of Psychology and the Center for Substance Use, Addiction & Health Research (CESAR), University of Maryland, College Park, Maryland, U.S.A<br>7. Department of Practice, Sciences, and Health Outcomes Research, University of Maryland School of Pharmacy, Baltimore, Maryland, U.S.A<br>8. Department of Psychiatry, University of Kentucky School of Medicine, Lexington, Kentucky, U.S.A<br>**9.** Department of Epidemiology and Public Health, University of Maryland School of Medicine, Baltimore, Maryland, U.S.A
	5b	***Names and contact information of trial sponsors.***<br>Ashley Michelle Utter<br>National Cancer Institute<br>Ashley.utter@nih.gov<br>240-276-5635
	5c	***Role of study sponsors and Funders, if any, in study design, collection, management, analysis and interpretation of data; writing the report, and the decision to submit the report for publication, including whether they will have ultimate authority over any of these activities.***

		The study sponsors and funders have no role in the above-listed activities and will not have ultimate authority over these activities.
	5d	***Composition, roles, and responsibilities of data monitoring committee***<br>Pg 53 of protocol and attached DSMB charter
**Introduction**		
**Background and rationale**	6a	**Pgs 9 – 11 of study protocol**<br>Expanded and sustained access to HIV treatment, resulting in substantial improvements in life-expectancy (1), has led to the need to address modifiable risk factors associated with leading causes of death among people living with HIV (PLWH), including cardiovascular disease and cancer (2–4). PLWH smoke at higher rates than the general population (5–10), and among PLWH, tobacco use has been consistently shown to impact both HIV-related (11,12) and non-HIV related co-morbidities (13–18). Tobacco smoking has also been identified as the leading cause of premature mortality (19–21). In addition, many PLWH who smoke would like to quit (7,22,23).<br>Although many interventions are effective in helping people who smoke to quit (24), research examining outcomes of smoking treatments among PLWH is concentrated in high-income countries. There is a critical need to assess the effectiveness of smoking cessation interventions among PLWH in lower- and middle-income countries and assess the delivery of these interventions within the clinical infrastructure available in these settings.<br>One evidence-based approach endorsed by the US Preventive services taskforce (24), and successful at supporting smoking cessation in a variety of settings, is the Screening, Brief Intervention, and Referral to Treatment (SBIRT) approach (25–29). SBIRT not only increases the likelihood of making a tobacco quit attempt among patients who receive a brief intervention, but it is also strongly and consistently associated with increased satisfaction with care provided (27). Even low-intensity SBIRT may prompt quit attempts, decrease cigarette use, and support quitting, if offered routinely (30). In addition, the reach of SBIRT interventions can be increased by delivering SBIRT using cadres of staff other than medical providers (31).<br>The likelihood of a successful quit attempt is increased if counseling is provided along with pharmacologic therapy (32), especially for individuals who smoke and are willing to quit. Several pharmacotherapies are available to assist with smoking cessation(24). However, while the use of nicotine replacement therapy (NRT) has mixed results (33,34), varenicline, a high-affinity partial agonist for the nicotinic acetylcholine receptor subtype, has been shown to be more efficacious than NRT or bupropion in a large pharmacotherapy smoking cessation trial (35) and when used among PLWH (36).<br>Botswana, an upper middle-income country with a high prevalence of HIV of 20.8 % among adults (15-64 years), has achieved epidemic control of HIV with 98 % of PLWH in Botswana on ARVs and 98 % with viral load suppression (37). In addition, Botswana has a high prevalence of cigarette smoking of 14.2% among persons aged 15 years and above (38). Botswana is particularly vulnerable to the sequelae of tobacco smoking because it is in a region expected to face the largest growth in tobacco consumption in the world (39). Though a high proportion of Batswana contemplated quitting in 2017, only 7% of persons who attempted to quit in 2016 were able to successfully quit (38).
	6b	***Explanation for choice comparators - Pg 17-18 of study protocol***<br><br>**Standard of care:** The stepped-wedge design allows each site to serve as its own control. Based on our preliminary data, it appears that some but not all clinicians screen for smoking behaviors. Few clinicians provide brief interventions or referral to treatment. To ensure each site has a similar baseline at the beginning of the study, each site will receive a short-quit smoking brochure that each clinic can provide to patients who are identified as smokers. This approach is often used as a control condition for smoking cessation efficacy trials, and we believe it will be useful in our planned effectiveness trial as well.

		**BSMART Intervention – SBIRT with varenicline**: SBIRT is a comprehensive, integrated, public health approach to the delivery of early intervention and treatment services for persons with substance use disorders that has been endorsed by the U.S. Preventive Services Taskforce. Varenicline has been shown to be more efficacious that nicotine replacement therapy and has been used successfully in PLWH.
**Objectives**	7	**Protocol pages 12 – 13**<br>The study objectives are as follows:<br>1. To assess the *Reach* and *Effectiveness* of BSMART.<br>i. Reach is defined as the proportion of PLWH who agree to participate and screen in the intervention program,<br>ii. Effectiveness is defined as a 7-day biochemically verified point prevalence abstinence from combustible tobacco products at 6 months using CO Check+. Our sample size (n=750 participants) has sufficient power to show at least 8% increase in a clinical effectiveness endpoint in the implementation group relative to standard of care (SOC) in the control group.<br><br>2. To assess the *Adoption* and *Implementation* of BSMART indexed by quality and consistency of intervention delivery.<br>i. identify how BSMART can be tailored to the network of HIV clinics in Botswana for adoption and implementation fidelity,<br>ii. foster comparison across clinical sites, and<br>iii. inform the development of activity logs to track implementation strategies.<br><br>3. To assess whether the intervention becomes *Maintained* as part of routine practices over time across HIV care facilities.<br>i. determine the extent to which the core components of BSMART and implementation strategies continue to be delivered and are maintained over time with fidelity and<br>ii. using the Program Sustainability Assessment Tool (PSAT), determine how organizational capacity and infrastructure to deliver BSMART are maintained, including partnerships, networks, and coalitions.<br>**4.** To determine the preliminary cost-effectiveness of BSMART - the incremental net monetary benefit at 6 months of follow-up in order to compare the value of BSMART to standard of care (SOC).<br>**Hypothesis:**<br>The BSMART intervention (SBIRT and varenicline) will<br>i. Increase the proportion of smokers able to quit smoking.<br>ii. Increase the number of attempts to quit smoking.<br>iii. Increase the length of abstinence in failed quit attempts.<br>iv. Decrease the number of cigarettes smoked every day.<br>v. Decrease the number of days of using combustible products within the past month.<br>vi. Decrease the number of days of using combustible products within the past three months in people exposed to the intervention as compared to people in the control phase.
**Trial design**	8	**P9 15 of study protocol**<br>BSMART is a stepped-wedge cluster randomized trial. This design includes an initial period when no clusters are exposed to the intervention then at regular steps, five clusters are randomized to cross from the control to the intervention (41). We will sequentially roll out the BSMART Intervention to 15 HIV care and treatment facilities. These facilities will be assigned in three steps, each providing data for a 12- month control/pre-intervention, a 12-month intervention, and a 12-month maintenance period (see Figure 2). We will stratify Botswana's

		HIV treatment and care facilities into three levels – district hospitals, primary hospitals, and primary clinics and randomly assign each of the three levels of facilities to one of three study steps. Each step will have five sites with representation from three levels of facilities: one district hospital, two primary hospitals and two primary clinics.<br>Our study employs a hybrid type 2 effectiveness-implementation design, which has equal attention to both effectiveness and implementation outcomes (42), to examine the effect of the BSMART Intervention (SBIRT and varenicline) on achieving abstinence from combustible tobacco products as compared to an enhanced standard of care among PLWH in Botswana who smoke and the effectiveness of our implementation.
**Study Setting**	9	We will sequentially roll out the BSMART Intervention to 15 HIV care and treatment facilities that are part of the ABLE (Accelerating Botswana through the Last Mile to Epidemic Control) project which is a five-year CDC-funded HIV care and treatment project. ABLE operates in 12 different PEPFAR health districts, 53 health facilities (13 hospitals and 40 clinics) and provides laboratory services that support all health districts. These 15 selected facilities are high volume facilities with over 1000 PLWH currently active and on antiretroviral therapy. LHWs and expert clients are in place in these facilities to ensure retention in treatment. Nurse prescribers are leveraged to manage the high volume of clients and to prescribe ARVs to stable clients on HIV treatment.
**Eligibility Criteria**	10	**Pg 16 of study protocol**<br>We will apply the following inclusion criteria for participants:<br>(1) Living with HIV,<br>(2) self-reported current daily smoker,<br>(3) age 18 years and older,<br>(4) engaged in HIV care as defined by being on ART for at least 6 months at one of 15 selected health facilities (or four reserve facilities), and<br>(5) willing/able to provide informed consent in English or Setswana.<br><br>We will apply the following exclusion criteria for participants:<br>(1) pregnancy or nursing
**Interventions**	11a	***Interventions for each group with sufficient detail to allow replication, including how and when they will be administered*.**<br>**Pgs 17 and 18 of study protocol**<br><br>**Enhanced Standard of care**<br>During the control phase, LHWs will provide an enhanced standard of care. This consists of providing participants with a brochure (Appendix I of study protocol) and a two- minute counseling session on the hazards of smoking and the benefits of quitting.<br><br>**BSMART Intervention – SBIRT and Varenicline**<br>SBIRT is a comprehensive, integrated, public health approach to the delivery of early intervention and treatment services to persons with substance use disorders endorsed by the US Preventive Services Taskforce (24). Trained LHWs will oversee the screening and brief intervention procedures using the 5As of SBIRT. The first "A" begins the intervention with LHWs "*Asking*" eligible clinic clients about smoking. Participants who report being daily smokers will be linked to a research assistant who will obtain informed consent and enroll the PLWH who smoke in the trial. The next 3 "A" s (*Advise, Assess, Assist*) constitute the brief intervention which will be delivered by LHWs using motivational enhancing conversations. Their efforts will focus the conversation on increasing insight and awareness regarding smoking, offering information and *Advice*, and *Assessing* motivation toward behavioral change. For participants who are motivated for treatment, a referral (*Assist*) will be made to a clinic nurse prescriber for

		evaluation for treatment with varenicline. For those not ready to make a quit attempt, the LHW will encourage consideration of quitting and *Arrange* for a follow-up conversation that will also encourage, advise, and assist in obtaining varenicline use.<br>Treatment with varenicline will be offered and provided to those motivated to quit. Smokers will initiate medication treatment with varenicline with a quit date scheduled for day 8 following the first study dose of the medication. They will meet with the nurse prescriber at baseline who will provide medical clearance and sign off on prescription orders. All medication will be provided to participants by the care team. Participants will receive a supply of medication for the first four weeks with subsequent weekly calls to ensure proper dosing and monitoring for adverse events. Participants will receive medication for the next eight weeks, at their Week four visit to the health facility. The dosage of varenicline will be in accordance with package labeling though dosage adjustments will be permitted to control adverse effects throughout the trial. This will allow us to balance internal validity with good clinical practice
	11b	***Criteria to discontinue or modifying allocated interventions for a given trial participant.***<br>**Pg 18 of study protocol.**<br>Criteria for Stopping<br>Withdrawal will take place in the case of significant side effects or at a participant's request.<br>Psychiatric emergencies: If suicidal ideation or intent is observed, the study suicide prevention plan (SPP) will be implemented, and the participant will be withdrawn. The SPP consists of immediate psychiatric evaluation, use of a suicide prevention contract, provision of 24-hour access to a physician, and referral to emergency services.
	11c.	***Strategies to improve adherence to intervention protocols and any procedures for monitoring adherence.***<br>Participants will receive a supply of medication at regular intervals supplemented by phone calls to ensure proper dosing and monitoring for adverse events. Adherence will be monitored during each of those study visits or phone calls and recorded on the medication adherence form (Appendix C4).
	11d	**Relevant concomitant care and interventions that are permitted or prohibited during the trial.**<br>HIV care and treatment will continue to be provided for study participants based on Botswana National Guidelines.
**Outcomes**	12	**Primary, secondary, and other outcomes including the specific measurement variables**<br>**Pgs 31 – 33 of study protocol**<br>1. Clinical Outcomes<br>Primary Outcome: The clinical effectiveness endpoint at six months is the 7-day point prevalence abstinence from combustible tobacco products validated primarily by breath CO < 6 ppm. The failure for this measure is any smoking (even a puff) during a 7-day window.<br>Secondary Outcomes:<br>• Quit attempts.<br>• Length of unsuccessful quitting during each attempt<br>• Number of cigarettes currently smoked, and<br>• Number of days of using combustible tobacco products for at least 24 hours within the past month and the last 3 months.
**Participant timeline**		**Time schedule of enrollment, interventions, assessments, and visits for participants**<br>**See Tables 2 and 3 on Pgs. 25 and 28 of study protocol**
**Sample Size**	14	**Estimated number of participants needed to achieve study objectives and how it was determined, including clinical and statistical assumptions supporting any sample size calculations.**<br>**Pgs 15 and 36 of the study protocol**<br>The study will screen approximately 6,900 HIV-infected patients and we expect to enroll 750 eligible participants, 375 of whom will participate in the pre-implementation phase and 375 of

		whom will participate in the implementation phase across three waves of implementation at five facilities each.<br>Our sample size is based on the hypothesis that our intervention will have a substantial impact on primary endpoints compared to the control phase (Figure 6).<br>**Main effect:** Based on a meta-analysis of 24 randomized controlled trials in LMIC (50), smoking abstinence at 6 months follow-up ($P_{control}$) for usual care averages 10% (range, 8% to 14%). Assuming withdrawal from trial follow-up or loss to follow-up of 5%, our sample of 25 PLWH per cluster per period has power of 87% to detect at least 10% increase (main effect, $\Delta = (P_{intervention} - P_{control})$ in the primary endpoint at 24 weeks with the assumption that the event rate for those in the control phase is 10% and two-sided alpha at intra-cluster correlation of 0.02.
**Recruitment**	15	**Strategies for achieving adequate participant enrollment to reach target sample size.**<br>Recruitment activities will begin at the HIV care services through the introduction of BSMART by LHWs. The LHWs will provide tobacco use screening within the context of HIV outpatient care. They will identify individuals who initially screen positive for tobacco use and link them with the research assistant stationed in the HIV clinic. Research assistants will obtain informed consent in the participant's choice of English or Setswana language from eligible individuals.<br>We plan to screen approximately 6,900 persons living with HIV (230 PLWH per facility per year) for tobacco smoking and subsequently identify 750 patients who are interested in and agree to participate in a smoking cessation trial; a total of 375 in the control period and 375 in the intervention period across three waves of implementation at five facilities each.<br>All sites to participate in the study have over 1000 patients actively engaged in receiving care at treatment.
**Methods: Assignment of Interventions (for controlled trials)**		
**Allocation: Sequence generation. Allocation concealment Implementation Blinding**	16a<br>16b<br>16c<br>17a<br>17b	Not applicable – BSMART is a cluster randomized study with a stepped wedge design. All sites will start with the control – standard of care and be moved in steps to the intervention.
**Methods: Data collection, management, and analysis**		
**Data collection methods**	18a	**Plans for assessment and collection of outcome, baseline, and other trial data, including any related processes to promote data quality and a description of study instruments.**<br>**Pg 34 and 54 of study protocol**<br>Data will be collected directly using tablets with REDCap Mobile Device Applications. The study team will work with the ABLE program staff to electronically extract all clinical and laboratory measurements longitudinally from the Integrated Patient Management System (IPMS) and the Patient Information Management System (PIMS); the two main EMR systems used in Botswana and supported by BUMMHI, UMB's affiliate organization and BSMART study partner. Based on our experience, we anticipate high data quality as part of the ABLE program. All study sites will be connected by telephone and e-mail. Data will be extracted directly into a REDCap database (hosted by BUMMHI) by study personnel and immediately available for viewing by the study coordinator and data manager. Data checks will be put in place to verify completeness for each site (against IPMS, PIMS) and to check for data errors, using modifications of automated checks. Computer programmers in Botswana and Baltimore will evaluate the database and check for inconsistencies and missing data on an ongoing basis, and queries will be directed back to each site to maintain data quality.
	18b	**Plans to promote participant retention and complete follow-up.**<br>**Pg 30 of study protocol**

		We will leverage the current approach which utilizes lay health workers and expert clients to ensure retention in the study and include additional strategies such as information leaflets about the importance of retention, appointment cards, motivational text messages, and phone calls (and home visits) to remind participants of appointments. All participants who miss study visits will be contacted.
**Data Management**	19	**Plans for data entry, coding, security, and storage, including any related processes to promote data quality**<br>**Pgs 34, 51 - 54 of study protocol.**<br>## Data Collection and Storage<br>All study data will be collected electronically by trained research assistants during study activities. Data entry to the tablet-based electronic database (REDCap) will be performed by trained study staff.<br>Research Electronic Data Capture- REDCap: REDCap is an application for building and managing online databases primarily created for electronic data collection, storage and transmission. UMB has developed and currently supports secure, local installation of REDCap for ongoing studies in Botswana. REDCap provides a web-based interface for collecting data with data validation and includes the ability for automated export to statistical packages. The software also includes data logging for HIPAA compliance and the ability for administrators to define access rights on a per-user basis.<br>## Data Safety and Security<br>Access credentials: Only authorized users with usernames and passwords will be given access to the REDCap database by the administrators. Users will be made aware that providing user IDs or sharing passwords with unauthorized individuals is a BREACH OF CONFIDENTIALITY and is grounds for appropriate disciplinary action. Access will be granted to only IRB approved study team members who are trained in protecting personal health information (PHI), have completed the CITI certification and who will access REDCap on secured networks and devices.<br>## Ensuring Data Quality<br>The approach to data quality is based on the following key points:<br>Components of data quality — Approach<br>1. Data Completeness — Minimum missing values<br>2. Data Accuracy — Matching values in the database with original observation<br>3. Data Precision — Defining units and measurability<br>4. Timely collection — Minimal time loss between observation and recording<br>5. Data Verification — Independent assessment or monitoring<br>6. Data Tracing — Logging actions taken while handling the data<br>Above points will be ensured as follows:<br>a) Data Validation and Queries<br>The data manager will assess data quality using REDCap's Data Quality module. Any identified issues will either be corrected or documented in the study data handling manual. Electronic data collection forms will be programmed with online validation checks. These checks will:<br>• Alert data entry users to missing data.<br>• Check that numeric variables and dates are within reasonable ranges.<br>• Check for consistency within the data.<br>b) Data Quality Audit<br>At intervals throughout the study a subset of entered data will be compared with the original data collection forms. The number of compared forms and error counts will be recorded in order to measure the percentage data entry error rate.<br>These audits will be conducted using random samples as follows:<br>• 10 of the first 100 subjects

		• 20 of the first 200 subjects<br>• 75 of the total 750 subjects<br>The error rate for study data will be maintained below 0.1%. If error rates remain high for three consecutive samples the team will need to consider alternative remedies in order to bring the error rate within acceptable limits.<br>**1. Data Extraction and Transfer**<br>Data can be exported by authorized users only, using REDCap's data export module. De-identified study data will be exported into an Excel sheet and transmitted via Dropbox to co-investigators on a weekly basis.<br>**2. Archiving and Destruction**<br>After study completion, all study materials will be stored in a locked shelf, inside a locked room at the PI's office at UMB. Subsequently, the study database on the REDCap system will be archived at UMB.
**Statistical Methods**	20a	**Statistical methods for analyzing primary and secondary outcomes. Pgs 37 - 40**<br>We will compare *Reach* - the percentage of PLWH who were screened for tobacco use for each facility between the control and implementation group using chi-square test.<br>*Effectiveness* – We will compare the clinical effectiveness endpoint between the control group and implementation group at the individual level with a generalized linear mixed model with a binary distribution using the jack-knife method to estimate standard errors to account for grouping within clusters and by incorporating a log-link function to estimate the relative risk as a measure of effect (41,44). We will include random effects to account for the clustering within facilities and periods and fixed effects for types of facilities. The model with unique covariance structure that produces the lowest Bayesian Information Criterion (BIC) value will be selected as the best model. The covariance structures that will be considered in the model are the first order of autocorrelation covariance structure, unstructured covariance structure, and Toeplitz covariance structure. We will use the Satterthwaite method to adjust for denominator degree of freedom for the test for fixed effects. The random coefficients will be modeled using G-side random effects, and we will obtain the subject-specific estimates by defining the appropriate variance-covariance structures.<br>We will perform sex-stratified and age-stratified analyses and models as secondary analyses. Given the high quality of data and experience in conducting studies in Botswana, we do not expect to have missing observations in the variables required for the primary analysis.<br>As analysis of secondary outcomes at 24 weeks, we will compare 30-day point prevalence abstinence between the implementation and the control groups in a similar model to the one used in the analysis of the primary outcome. We will compare longitudinal differences in the Stages of Change Algorithm between the implementation and the control groups using random-mixed effects regression. We will use generalized estimating equation models for binary repeated measures to assess factors associated with completion of the varenicline medication (45).<br>Implementation – We will assess implementation monthly for the intervention period. We will use Pearson's correlation to assess the strength of correlation between qualitatively derived construct ratings from qualitative interviews and implementation effectiveness across facilities.<br>*Maintenance* – We will reassess RE-AIM measures 12 - 24 months after BSMART implementation to provide a standardized evaluation approach to foster understanding of whether the impact and implementation delivery are maintained and to highlight where sustainability issues arise.<br>*Preliminary cost-effectiveness* - The incremental cost-effectiveness ratio (ICER) is calculated as the ratio of the difference in mean costs and the difference in mean 7-day point prevalence abstinence at 6 months. The ICER (Figure 4) quantifies the additional cost associated with a unit change in the 7-day point prevalence abstinence at six months, comparing between the implementation and control groups. Net benefit (NB) regression (46,47) provides an appropriate

		approach to quantify the ICER using the incremental net monetary benefit (INMB) and produces a confidence interval around the ICER.<br>**Figure 4: Incremental cost-effectiveness ratio**<br><br>In equation 1 of Figure 5 we define NB as the difference between the monetized total effect measure (the product of lambda, the willingness to pay per unit of effect, and the total effect units available, e) and the associated costs. The NB regression model is specified in equation 2 of Figure 5 as the difference between effects and costs, estimated via a parametric regression framework where errors are represented by the final term in equation 2, epsilon. We will identify the initial value of lambda following a targeted literature review and consultations with onsite study collaborators. In equation 2 of Figure 5, the NMB, represented by the β coefficient on the indicator variable for receipt of intervention, will be estimated using the NB regression model.<br>**Figure 5: Net Benefit**
	20b	**Methods for any additional analysis pg 40**<br><u>Sensitivity Analysis:</u> We will represent uncertainty in the estimated incremental cost-effectiveness ratios (ICERs) using the cost-effectiveness acceptability curve (46,48). In addition, we will represent parameter uncertainty in the choice of the economically preferred intervention (between control and intervention periods) by the conditional net benefit (cNB) curve. The cNB curve plots the cNB (i.e., the net benefit value given a particular value of the parameter of interest) across centiles of the distribution of the parameter of interest.<br>Sensitivity analyses will include probabilistic one-way sensitivity analyses (POSA)(49) of cost input parameters (such as drug costs). Uncertainty in cost inputs will be investigated via designed simulations that use alternative values of cost inputs as determined by draws from assigned distributions (for example gamma, and lognormal), with distributional parameters estimated from data collected from ongoing studies in Botswana. The POSA varies a specific input parameter value across its full distribution while accounting for concurrent variation in all other parameter values using Monte Carlo simulation. The cNB curve developed from the planned POSA will allow decision makers to identify the impact of the value taken by a specific cost parameter on the value of the BSMAART intervention.
	20c	**Definition of analysis population relating to protocol non-adherence, and any statistical methods to handle**.<br>Intention to treat analyses based on the services provided at the facility during the time-period the participant was enrolled will be employed.<br>Duration of use of varenicline, number of visits completed, and timeliness of study visits will be included as variables in the analysis.
**Methods: Monitoring**		
**Data Monitoring**	21a	**Composition of Data monitoring Committee**<br>See attached Data monitoring committee charter
	21b	**Description of any interim analyses and stopping guidelines**<br>**Pg 53 of the study protocol**<br>A formal interim analysis is not anticipated, as it is important to measure the effect of the intervention over the full proposed period of time. However, data from the study communities on operational performance including uptake and adverse events will be reported to the DSMB and reviewed on a monthly basis via electronic data capture, facilitating timely analysis. We will monitor adverse outcomes and other items above during the intervention on a monthly basis. We will convene the DSMB every six months during the intervention period in order to review progress and safety issues.
**Harms**	22	**Plans for collecting, assessing, reporting, and managing solicited and spontaneously reported adverse events and other unintended effects.**<br>**Pgs. 51 - 53 of study protocol**<br><u>Serious Adverse Event (SAE) Reporting:</u> Safety monitoring for this study will focus on reportable new information of events that include unanticipated problems involving risks to participants, including unanticipated problems that meet the definition of a serious adverse event.

		Unanticipated Problems: The Office for Human Research Protections (OHRP) considers unanticipated problems involving risks to subjects or others to include, in general, any incident, experience, or outcome that meets all of the following criteria:<br>• Unexpected in terms of nature, severity, or frequency given (a) the research procedures that are described in the protocol-related documents, such as the IRB-approved research protocol and informed consent document; and (b) the characteristics of the subject population being studied;<br>• Related or possibly related to participation in the research (possibly related means there is a reasonable possibility that the incident, experience, or outcome may have been caused by the procedures involved in the research); and<br>• Suggests that the research places subjects or others at a greater risk of harm (including physical, psychological, economic, or social harm) than was previously known or recognized.<br>Serious Adverse Events: A serious adverse event (SAE) is one that meets one or more of the following criteria:<br>• Results in death<br>• Is life-threatening (places the subject at immediate risk of death from the event as it occurred)<br>• Results in inpatient hospitalization or prolongation of existing hospitalization<br>• Results in a persistent or significant disability or incapacity<br>• Results in a congenital anomaly or birth defect<br>An important medical event that may not result in death, be life threatening, or require hospitalization may be considered an SAE when, based upon appropriate medical judgment, the event may jeopardize the subject and may require medical or surgical intervention to prevent one of the outcomes listed in this definition.
**Auditing**	23	**Frequency and procedures for auditing trial conduct, if any, and whether the process will be independent from investigators and the sponsor.**<br>There will be no trial audit in addition to the monitoring provided by the DSMB.
**Ethics and Dissemination**		
**Research Ethics and Approval**	24	**Plans for seeking research ethics committee/ institutional review board approval.**<br>The Botswana Smoking Abstinence Reinforcement Trial study received approvals from the Human Resource Development Council of Botswana and the University of Maryland Baltimore Institutional Review Boards. Informed consent will be obtained from all participants.
**Protocol amendments**	25	**Plans for communicating important protocol modifications to relevant parties.**<br>Participants will be notified of changes that affect participation or study activities via consent and study staff are provided with current approved documents and discussion of those changes occur during standing meetings.
**Consent or assent**	26a	**Who will obtain informed consent/ assent from potential trial participants or authorized surrogates?**<br>**Pg. 26 and 27 of the study protocol**<br>Research assistants will obtain informed consent from eligible participants in their choice of English or Setswana.
	26b	**Additional consent provisions for collection and use of participant data and biological specimens in ancillary studies**<br>Appendix A1 and A2 consent forms for the control and intervention phases in the protocol appendix have been modified to include consent for contact for future studies. The modification is yet to be submitted for IRB approval.
**Confidentiality**	27	**How personal information about potential and enrolled participants will be collected, shared, and maintained in order to protect confidentiality.**<br>**Pg 51 of study protocol**

		Study participants will be assigned a coded identification number at the time of study enrollment. In order to maintain patient confidentiality, all laboratory specimens, study case report forms, and reports will be identified using that coded number. Only research staff will have access to the unique coded number. Key study personnel will store research data (including medical and laboratory records) in locked cabinets, and all e-files will be password protected. Data will be stored in password-protected files. Access to all data will be ID- and password protected, including data warehouse software and computers managing and analyzing survey data. No data will be released with any information that may directly or indirectly identify participants, their clinical information, or laboratory results to outside agencies.
**Declaration of interests**	28	**Financial and other competing interests for the principal investigators for the overall trial and each study site**<br>The PI and other investigators declare that they have no competing interests.
**Access to data**	29	**Statement of who will have access to the final trial dataset and disclosure of contractual agreements that limit such access.**<br>There are no contractual agreements to limit access to the dataset by study investigators and such access will be under the direction of the study PI/PD. All data published will have the associated dataset included in supplemental information.
**Ancillary and post-trial care**	30	**Provisions, if any, for ancillary and post-trial care, and for compensation for those who suffer harm from trial participation.**<br>Participants will continue to receive care for their HIV infection at the clinic in which they are recruited for the study.<br>Participants who have an emergency are advised to go to the nearest health center and for other injuries, to report to their healthcare provider.
**Dissemination policy**	31a	**Plans for investigators and sponsors to communicate trial results to participants, healthcare professionals, the public, and other relevant groups.**<br>**Pgs 56 – 57 of study protocol**<br><br>**Dissemination Plan**<br>We have plans to disseminate findings from the proposed study at various levels, in compliance with NIH policy:<br>1. ClinicalTrials.gov: We will register BSMART within the first 6 months of the funding award.<br>• All submitted information will be updated semi-annually or when modifications are required.<br>• Any apparent errors, deficiencies, and/or inconsistencies identified by the NIH as part of the quality control review process will be addressed by Dr. Charurat, MPI (Contact).<br>• Corrections to submitted information will be made within 15 days for registration information and 25 days for results information.<br>• Trial results will be submitted no later than one year after the primary completion date.<br>• Informed consent documents for the trial enrollment will include specific statement relating to posting of the clinical trial and results at ClinicalTrials.gov.<br>• The University of Maryland Baltimore has an internal policy in place to ensure that clinical trial registration and result reporting occur in compliance with the NIH policy requirements.<br>2. Local Dissemination of study findings: We have engaged the community in the study design. In addition, our experiences with previous studies in Botswana suggest that sharing aggregate data from the trial with stakeholders, including NAHPA, MOHW, the Anti-tobacco Network and the community members, builds significant trust. We will provide each relevant stakeholder with aggregate information derived from study data in meaningful and appropriate ways to improve health access, outcomes, and equity.

		The Stakeholder Advisory Group and Practitioner Engagement Group will provide ongoing input into dissemination efforts.<br>3. <u>National Dissemination:</u> To ensure successful integration of our proposed research into broader practice and policy, we will continue to leverage our strong partnerships with the MOHW, NAHPA, UB, civil societies, and PEPFAR/CDC in Botswana. Training of LHW Case Managers is an essential part of a cost-effective, evidence-based strategy on smoking cessation and treatment of tobacco dependence because of their interaction with smokers and tobacco consumers as care providers and their role as health communicators. We will also leverage our relationship with the Anti-tobacco Network as a technical partner of the study (Dr. Mbongwe, BSMART MPI) to re-invigorate the national smoking cessation campaign that is underpinned by interventions that have been developed and tested locally.<br>4. <u>Regional Dissemination:</u> We will also focus on sharing findings at regional meetings in sub-Saharan Africa, such as the African Organization for Research and Training in Cancer (AORTIC) annual conference. We will submit abstracts for presentation at this conference to share our study findings.<br>5. <u>International Dissemination:</u> We will submit abstracts to international conferences such as the Consortium of Universities for Global Health (CUGH) and the International AIDS Society conference.<br>**6.** <u>Publications:</u> We expect to have a series of peer-reviewed articles generated from our study throughout the project period and have allotted funds in our budget to make these publications open access for ease of reading, especially for those in other low- and middle-income countries (LMICs).
	31b	**Authorship eligibility guidelines and any intended use of professional writers**<br>Standardized guidelines for authorship will be adhered to. All authors to be included are required to make substantial contributions to the conception or design of the work; or the acquisition, analysis, or interpretation of the work, drafting the work or reviewing it critically, approving the final version of any manuscript for publication, and responsibility for the accuracy and integrity of all aspects of research (International Committee of Medical Journal Editors).<br>Professional writers will not be used.
	31c	**Plans, if any, for granting the public full access to the study protocol, participant-level dataset, and statistical code.**<br>A description of this study is available at ClinicalTrials.gov. There are no other plans in place to grant access to the full study protocol. Statistical code will only be provided as supplemental information for published manuscripts.
Informed consent materials	32	**Model consent form and other related documents.**<br>Appendix A1 – A5 on pages 62 – 91 of the study protocol contain five approved consent forms.
Biological specimens	33	**Plans for the collection, laboratory evaluation, and storage of biological specimens for genetic or molecular analysis in the current trial and for future use in ancillary studies**.<br>Not applicable – biological specimens will not be collected in this trial.
