# Supplementary material for: Design and rationale of the Botswana Smoking Abstinence Reinforcement Trial: a protocol for a stepped-wedge cluster randomized trial
Source: Implement Sci Commun. 2024 May 8;5:53. doi: 10.1186/s43058-024-00588-7 (PMC11077839; doi:10.1186/s43058-024-00588-7)
Supplement: Supplementary file 1 — Supplementary Material 1. [file BSMART Local IRB ApprovalR0.pdf › BSMART Local IRB ApprovalR0.pdf]

TELEPHONE: 363 2500
FAX: 317 0155
TELEGRAMS: RABONGAKA
TELEX: 2818 CARE BD

Republic of Botswana

MINISTRY OF HEALTH
PRIVATE BAG 0038
GABORONE

REFERENCE NO: HPRD: 6/14/1 **08 May 2023**

Health Research and Development Division

Notification of IRB Review: **New application**

Dr. Man Charurat,
Institute of Human Virology,
University of Maryland,
Baltimore Baltimore, MD 21201-1508

Dear Dr. Man Charurat

PROTOCOL TITLE: BOTSWANA SMOKING ABSTINENCE REINFORCEMENT TRIAL

Review Type: Expedited
Review Date: 27th April 2023
Approval Date: 08th May 2023
Effective Date: 08th May 2023
Expiration Date: 07th May 2024
Risk Determination: Minimal risk

Thank you for submitting new application for the above referenced protocol. The permission is granted to conduct the study.

This permit does not however give you authority to collect data from the selected sites without prior approval from the management. Consent from the identified individuals should be obtained where applicable.

The research should be conducted as outlined in the approved proposal. Any changes to the approved proposal must be submitted to the Health Research and Development Division in the Ministry of Health for consideration and approval.

Furthermore, you are requested to submit at least one hardcopy and an electronic copy of the report to the Health Research, Ministry of Health and Wellness within 3 months of completion of the study.Copies should also be submitted to all other relevant authorities.

Continuing Review

In order to continue work on this study (including data analysis) beyond the expiry date, submit a Continuing Review Form for Approval at least three (3) months prior to the protocol's expiration date. The Continuing Review Form can be obtained from the Health Research Division Office (HRDD), Office No. 7A.7 or Ministry of Health website: www.moh.gov.bw or can be requested via e-mail from HRDD office, e-mail address: hhealthresearch@govbots.onmicrosoft.com. As a courtesy, the HRDD will send you a reminder email about eight (8) weeks before the lapse date, but failure to receive it does not affect your responsibility to submit a timely Continuing Report form.

Amendments

During the approval period, if you propose any change to the protocol such as its funding source, recruiting materials, or consent documents, you must seek HRDC approval before implementing it. Please summarize the proposed change and the rationale for it in the amendment form available from the Health Research Division Office (HRDD), Office No. 7A 7 or Ministry of Health website: www.moh.gov.bw or can be requested via e- mail from HRDD Office, e-mail address: hhealthresearch@govbots.onmicrosoft.com. In addition submit a copy of an updated version of your original protocol application showing all proposed changes in bold or "track changes".

Reporting

Other events which must be reported promptly in writing to the HRDC include:

- *Suspension or termination of the protocol by you or the grantor*
- *Unexpected problems involving risk to subjects or others*
- Adverse events, including unanticipated or anticipated but severe physical harm to subjects.

If you have any questions please do not hesitate to contact, Mr Abia Sebaka, asebaka@gov.bw , Tel +267-3632754 or Mr. Kgomotso Motlhanka at kgmmotlhanka@gov.bw, Tel +267-3632751.

Thank you for your cooperation and your commitment to the protection of human participants in research.

Yours sincerely

Mr Kgomotso Motlhanka
for /PERMANENT SECRETARY

MINISTRY OF HEALTH
RESEARCH DIVISION
08 MAY 2023
P/BAG 0038, GABORONE
HEALTH POLICY RESEARCH & DEVELOPMENT
